# Design of a Potent, Selective, and Brain-Penetrant Inhibitor of Wnt-Deactivating Enzyme Notum by Optimization of a Crystallographic Fragment Hit

**DOI:** 10.1021/acs.jmedchem.2c00162

**Published:** 2022-05-10

**Authors:** Nicky J. Willis, William Mahy, James Sipthorp, Yuguang Zhao, Hannah L. Woodward, Benjamin N. Atkinson, Elliott D. Bayle, Fredrik Svensson, Sarah Frew, Fiona Jeganathan, Amy Monaghan, Stefano Benvegnù, Sarah Jolly, Luca Vecchia, Reinis R. Ruza, Svend Kjær, Steven Howell, Ambrosius P. Snijders, Magda Bictash, Patricia C. Salinas, Jean-Paul Vincent, E. Yvonne Jones, Paul Whiting, Paul V. Fish

**Affiliations:** † Alzheimer’s Research UK UCL Drug Discovery Institute, University College London, Cruciform Building, Gower Street, London WC1E 6BT, U.K.; ‡ The Francis Crick Institute, 1 Midland Road, Kings Cross, London NW1 1AT, U.K.; § Division of Structural Biology, Wellcome Centre for Human Genetics, University of Oxford, The Henry Wellcome Building for Genomic Medicine, Roosevelt Drive, Oxford OX3 7BN, U.K.; ∥ Department of Cell and Developmental Biology, Laboratory for Molecular and Cellular Biology, University College London, London WC1E 6BT, U.K.

## Abstract

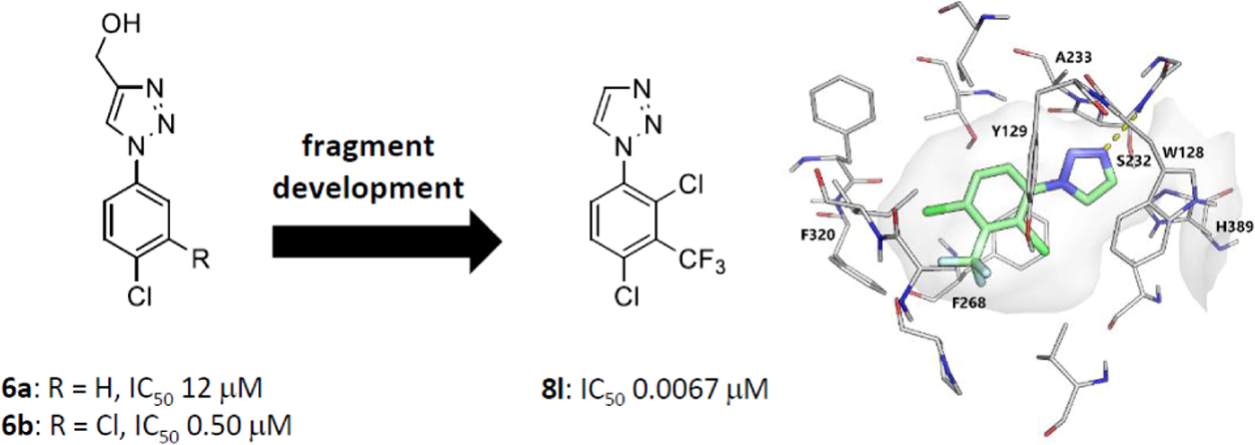

Notum is a carboxylesterase that suppresses Wnt signaling through deacylation of an essential palmitoleate group on Wnt proteins. There is a growing understanding of the role Notum plays in human diseases such as colorectal cancer and Alzheimer’s disease, supporting the need to discover improved inhibitors, especially for use in models of neurodegeneration. Here, we have described the discovery and profile of **8l** (ARUK3001185) as a potent, selective, and brain-penetrant inhibitor of Notum activity suitable for oral dosing in rodent models of disease. Crystallographic fragment screening of the Diamond-SGC Poised Library for binding to Notum, supported by a biochemical enzyme assay to rank inhibition activity, identified **6a** and **6b** as a pair of outstanding hits. Fragment development of **6** delivered **8l** that restored Wnt signaling in the presence of Notum in a cell-based reporter assay. Assessment in pharmacology screens showed **8l** to be selective against serine hydrolases, kinases, and drug targets.

## Introduction

The Wnt signaling pathway is a complex, evolutionarily conserved system from *Drosophila* and *Caenorhabditis elegans* through to mammals, which plays key roles in both embryonic development and the adult animal. These roles include cell fate determination and maintenance, and cell division in many different tissues.^1^ The “canonical Wnt signaling pathway” is characterized by the secreted activator (or agonist) Wnt proteins, which initiate an intracellular signaling cascade by binding to the cell surface receptor (a member of the Frizzled family of cell surface proteins, complexed with the coreceptor LDL-receptor-related protein family 5/6).^1^ The intracellular signaling cascade utilizes β-catenin and GSK3β to activate gene transcription via T-cell factor (TCF)/lymphoid enhancing factor (LEF) transcription factors. This signaling system is tightly regulated by a complex network of modulators and feedback inhibitory proteins, including the family of dickkopf (Dkk) proteins^2^ and the carboxylesterase Notum.^3,4^ Given the pleiotropic and essential function of Wnt signaling, it is unsurprising that aberrant Wnt signaling can contribute to disease in humans, including certain cancers, diabetes, and osteoporosis.^1,5^ In the mammalian brain, Wnt signaling performs several important roles, including the maintenance of synapse function,^6^ modulating microglial signaling and survival,^7^ neurogenesis,^8^ and maintaining the integrity and function of the blood–brain barrier (BBB).^9,10^ In Alzheimer’s disease (AD), when the Wnt signaling tone is disrupted by an increase in the inhibitory Dkk proteins, this leads to synapse loss, which may contribute to cognitive impairment.^11^ In stroke, loss of Wnt signaling leads to BBB breakdown, contributing to the pathology.^12,13^ In a model of multiple sclerosis, reactivation of Wnt signaling at the BBB partially restores functional BBB integrity and limits immune cell infiltration into the CNS.^14^ Therefore, augmenting Wnt signaling in these disorders of the central nervous system (CNS) has therapeutic potential.

Notum is a negative regulator of the Wnt signaling pathway. It is a secreted protein that functions as a carboxylesterase, removing a palmitoleate ester from a conserved serine residue in Wnt proteins that is essential for its binding and activation of the cell surface coreceptors.^3^ While first identified in *Drosophila*, its function in mammals, in both healthy and diseased, is being increasingly explored. The key tools to enable this progress are fit-for-purpose small-molecule inhibitors of Notum carboxylesterase activity.^15,16^

**Figure 1:**
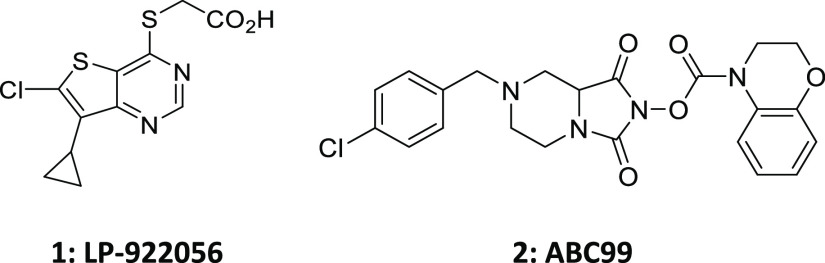
First-generation Notum inhibitors used in disease association studies.

First-generation inhibitors of Notum activity were LP-922056 (**1**)^17^ and the irreversible inhibitor ABC99 (**2**)^18^ that have proved to be valuable research tools in exploring the link between pharmacological inhibition of Notum activity and disease association in rodent models (Figure 1). LP-922056 is a potent inhibitor of Notum suitable for chronic, oral dosing in rodents discovered through the optimization of a high-throughput screening (HTS) hit.^17^ LP-922056 was used to show that inhibition of Notum activity is a potential novel anabolic therapy for strengthening the cortical bone and preventing non-vertebral fractures.^19^ Notum has recently been identified as a key mediator for people at high risk of developing colorectal cancer.^20,21^ Pharmacological inhibition of Notum with LP-922056 abolished the ability of *Apc*-mutant cells to expand and form intestinal adenomas.^21^ ABC99 is a potent and selective covalent inhibitor of Notum activity developed by competitive activity-based protein profiling (ABPP) from a library of activated carbamates.^18^ Inhibition of Notum produced by Paneth cells in mice with ABC99 restored the regeneration of aged intestinal epithelium.^22^ In the brain, Notum regulates neurogenesis in the subventricular zone (SVZ) by modulating Wnt signaling, and inhibition of Notum with ABC99 leads to an activation of Wnt signaling and increased proliferation in the SVZ.^8^

**Figure 2:**
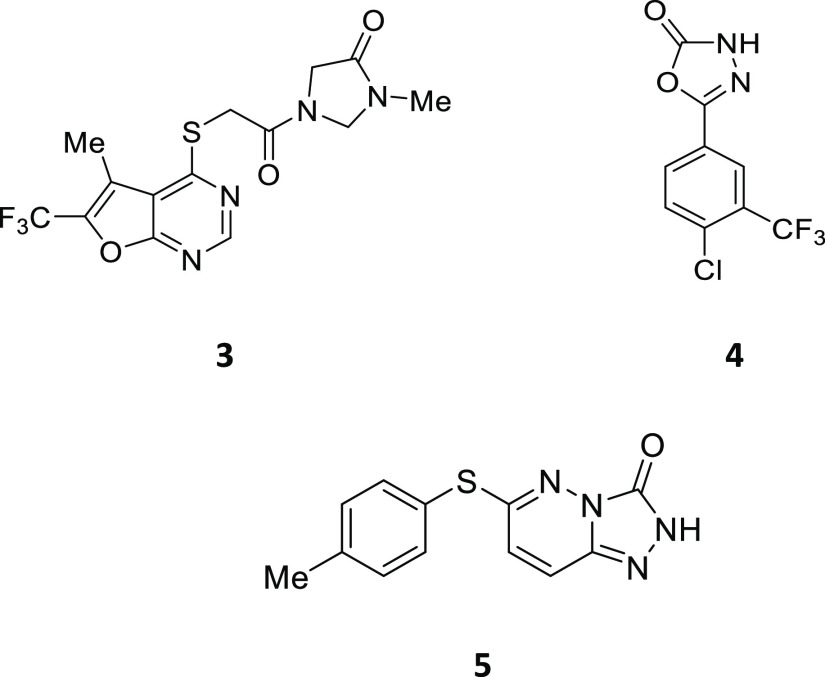
Representative chemical structures of Notum inhibitors.

A number of structurally distinct Notum inhibitors have been reported from hit-to-lead programs (Figure 2).^23−27^ However, most of these inhibitors have limited brain penetration as directly measured in mouse pharmacokinetic (PK) studies;^24,26−28^ this restricts their use to peripheral (non-CNS) indications. Only ABC99 has been reported to reach the brain and activate the Wnt pathway as assessed by qRT-PCR of *Axin2* in the ventricular-SVZ tissue following an intraperitoneal (i.p.) injection to *Nestin-CFP* mice.^8^

Our objective was to identify a screening hit that could be optimized to a potent, selective, and brain-penetrant inhibitor of Notum activity suitable for chronic, oral delivery. Such an inhibitor would complement LP-922056 and ABC99 as it could be administered in rodent models of neurodegenerative disease.

Fragment-based drug discovery (FBDD) is an established method for hit finding of biologically active compounds and is now supported by multiple complementary screening technologies.^29,30^ The concept of a “fragment” has essentially remained consistent with an emphasis on small, low-molecular-weight organic molecules with high aqueous solubility as described by Congreve et al.^31^ In contrast, there is an increase in the application of FBDD to more challenging, non-traditional targets such as GPCRs and ion channels, and to phenotypical screens including parasites.^29^ The creation of fragment libraries with a clearly defined purpose beyond chemical diversity has also received considerable attention. Examples of such libraries include reactive covalent fragments,^32^ 3D fragments,^33^ natural product-like compounds,^34^ very small fragments such as FragLites and Minifrags,^35^ halogen-enriched fragments,^36^ and synthetically poised fragment libraries.^37^

The original Diamond-SGC Poised Library (DSPL) was created by Cox et al. as a 768-membered fragment library that was designed to allow rapid follow-up synthesis to provide quick structure–activity relationships (SARs) around fragment screening hits. Each member of the collection contained at least one functional group that could be created with reliable chemical reactions, that is, the fragment library was created to be structurally diverse and synthetically enabled.^37,38^

Herein, we describe the discovery of crystallographic fragment screening hits **6a** and **6b** from the DSPL and their optimization to identify 1-phenyl-1,2,3-triazoles as a new Notum inhibitor chemotype. We present the profile of lead compound **8l** that supports it to be a potent, selective, and brain-penetrant inhibitor of Notum activity.

## Results and Discussion

In order to find hit compounds for the development of new Notum inhibitors, we chose to conduct a crystallographic fragment screen. The structure of Notum has a clearly defined lipophilic pocket adjacent to the catalytic triad that accommodates a C16-lipid palmitoleate group of Wnt.^3,39^ The size and topography of this pocket gave encouragement that suitable fragments could be found to bind as the pocket has been assessed to be classically druggable.^40^

An X-ray crystallography-based fragment screen was performed at the XChem platform of Diamond Light Source (Oxford, U.K.). The highly automated XChem platform, in combination with synchrotron beamline I04-1, enabled us to efficiently screen 768 compounds from the DSPL fragment library for binding to Notum. Compounds were individually soaked into crystals of Notum and high-resolution structures were determined for each of these putative Notum–fragment complexes. The electron density maps provided evidence of compound binding associated with the active site of Notum for 59 of the crystal structures (hit rate: 7.7%).^23,26,41^ The complete output of the hits from this crystallographic screen with the DPSL will be published separately.^42,43^ Hit validation was then performed by demonstration that these binders were able to inhibit Notum carboxylesterase activity in a biochemical assay (up to 1 mM).

**Figure 3:**
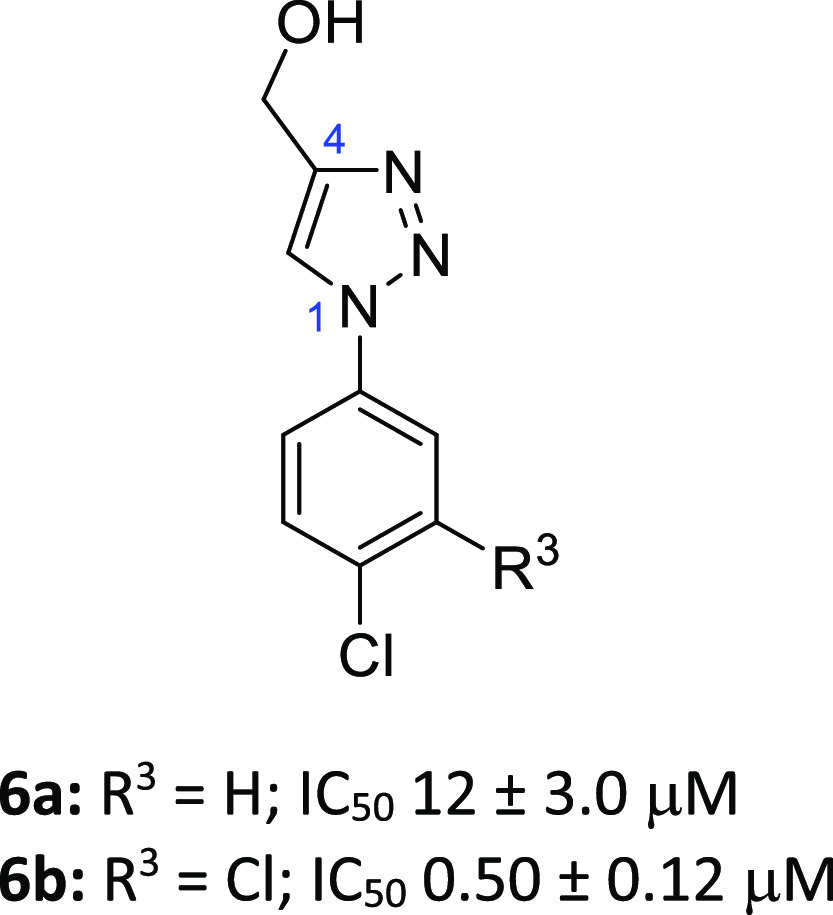
Preferred hits **6a** and **6b** from the crystallographic screen of the DSPL fragment library.

**Figure 4:**
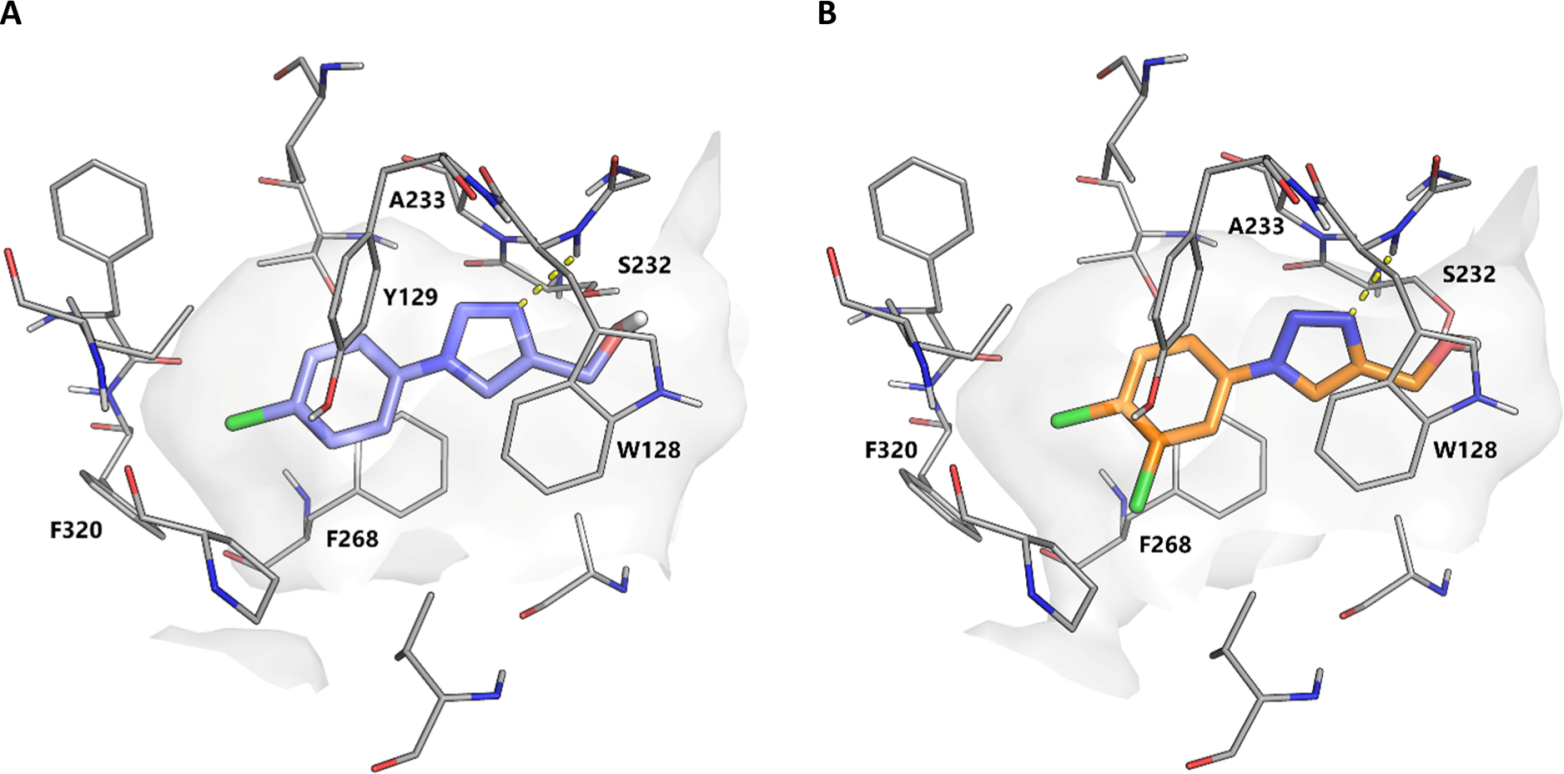
(A) Structure of **6a** (PDB: 6ZUV). N3 of the triazole forms a hydrogen bond with the backbone of Trp128 (dashed line, 2.36Å) (ref (26)). (B) Structure of **6b** (PDB: 7B89). The compound forms a hydrogen bond with the backbone of Trp128 (dashed line, 2.21 Å). Select binding site residues are shown. Water molecules have been removed for clarity. The surface of the Notum binding pocket is outlined (gray).

A pair of outstanding hits were 4-(hydroxymethyl)triazoles **6a** (IC_50_ 12 μM)^26^ and **6b** (IC_50_ 0.5 μM), which also highlighted some early beneficial SAR (Figure 3). A 3,4-Cl_2_ disubstituted phenyl ring conferred a substantial improvement in Notum inhibition when compared to a single 4-Cl (23-fold). Both the Notum-**6a** and -**6b** X-ray structures showed that the ligand formed a H-bond between N3 of the triazole with the backbone of Trp128. The increase in potency of **6b** over **6a** can be attributed to the improved occupancy of the palmitoleate pocket by the 3,4-Cl_2_ disubstituted phenyl ring (Figures 4 and S1). Specific interactions in the pocket include the potential stacking of the ligand with Trp128, Tyr129, and Phe268 as well as interactions by the phenyl ring substituents with Phe320.

Triazole **6b** has molecular and physicochemical properties consistent with lead-like chemical space^44^ (MW 2444; *c*logP, 2.2; LogD_7.4_, 2.4; TPSA 51 Å^2^) and scored high when assessed by design metrics (LE 0.59; LLE 4.1),^45,46^ including a favorable prediction of brain penetration (CNS MPO 5.6/6.0; BBB Score 4.9/6.0).^47,48^ Triazole **6b** was further evaluated in standard in vitro ADME assays and showed good aqueous solubility, moderate stability in liver microsomes, and excellent cell permeability (Table 1).

**Table 1:**
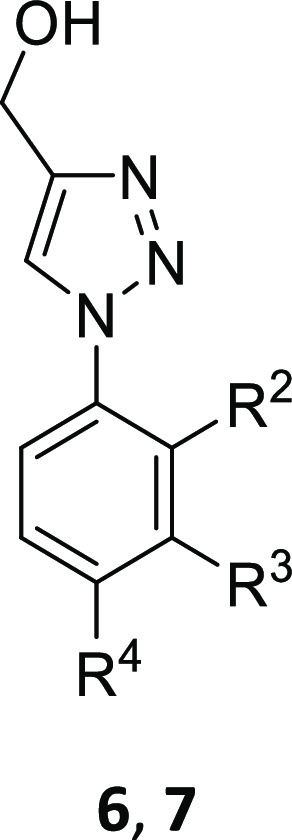

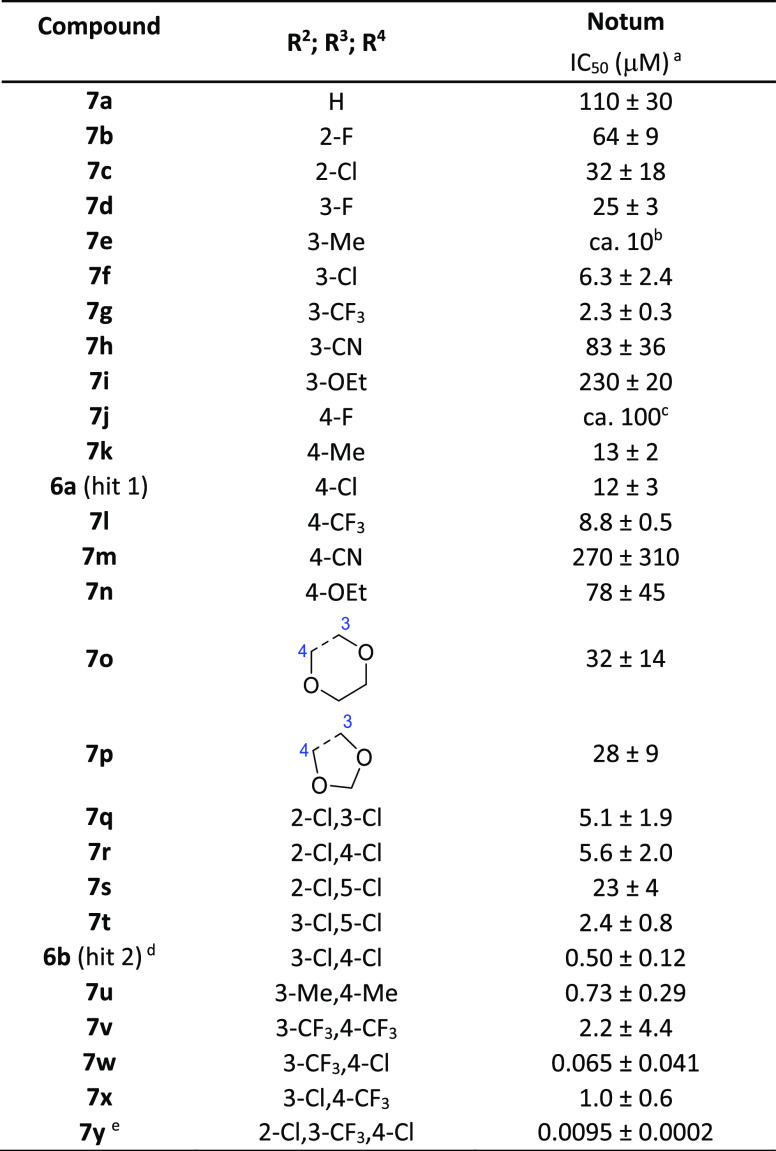
Notum Inhibition SAR of Substituted (1-Phenyl-1*H*-1,2,3-triazol-4-yl)methanols **6** and **7**

a All values are mean ± s.d. of *n* = 2–13 experiments quoted to 2 s.f.

b 40–50% I@10 μM.

c 40–50% I@100 μM.

d In vitro ADME data for **6b**: LogD_7.4_ 2.4; aqueous solubility, 100 μg/mL; mouse liver microsomes (MLM), Cl_i_ 88 μL/min/mg protein; human liver microsomes (HLM), Cl_i_ 12 μL/min/mg protein; MDCK-MDR1, AB/BA *P*_app_ 57/59 × 10^–6^ cm s^–1^; efflux ratio (ER), 1.0.

e In vitro ADME data for **7y**: aqueous solubility, 210 μg/mL; MLM, Cl_i_ 6.7 μL/min/mg protein; mouse hepatocytes, Cl_i_ 48 μL/min/10^6^ cells; MDCK-MDR1, AB/BA *P*_app_ 46/28 × 10^–6^ cm s^–1^; ER, 0.61.

Hits **6** were an attractive starting point to improve Notum activity and develop PK properties for oral dosing to achieve good plasma exposure and brain penetration.

The optimization of **6** to deliver this profile was directed at three principle areas of these hit structures: (1) aryl ring **7** (Table 1); (2) the C4 substituent on triazole **8** (Table 2); and (3) further fine-tuning of the triazole **9** (Table 3). Inhibition of Notum activity was routinely measured in a biochemical assay with human Notum (81–451 Cys330Ser) as the enzyme and trisodium 8-octanoyloxypyrene-1,3,6-trisulfonate (OPTS) as the substrate.^23−26^ This assay was used to establish SAR and to identify preferred leads for additional studies. As Notum activity was developed, representative examples were screened in standard in vitro ADME assays to assess their suitability for progression to mouse PK studies.

**Table 2:**
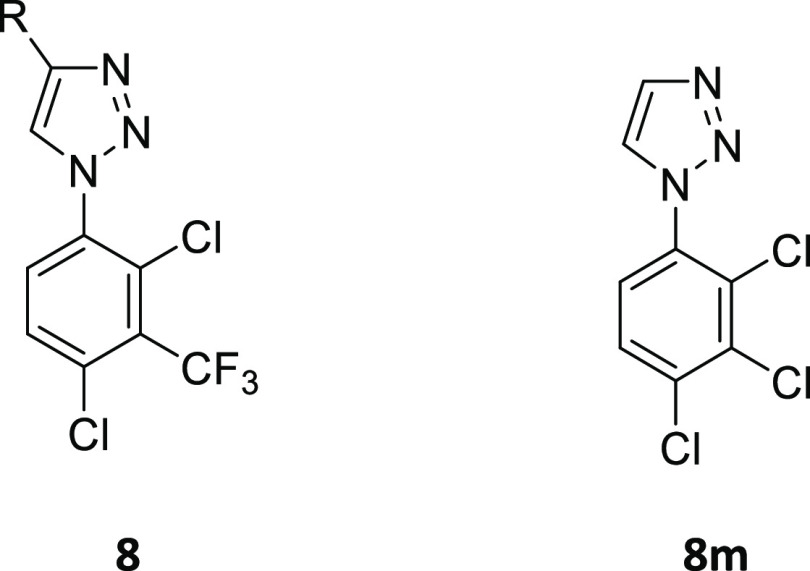

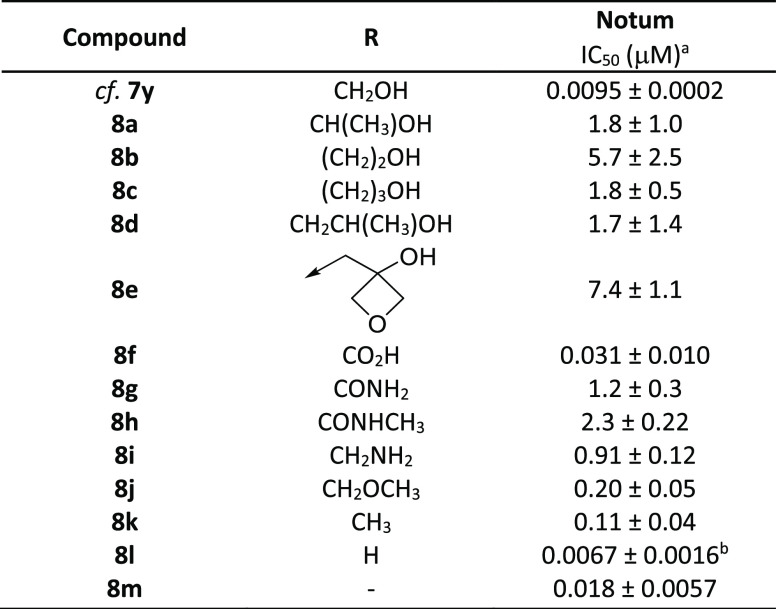
Notum Inhibition SAR of Substituted 1-(2,4-Dichloro-3-(trifluoromethyl)phenyl)-1*H*-1,2,3-triazoles **8**

a All values are mean ± s.d. of *n* = 2–6 experiments quoted to 2 s.f.

b *n* > 20.

**Table 3:**
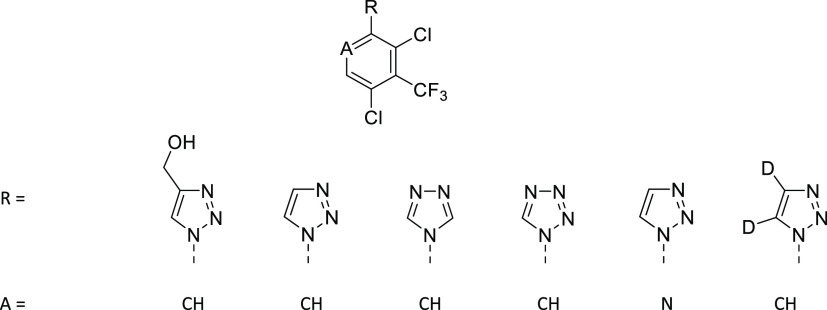
Notum Inhibition, Aqueous Solubility, Microsomal Stability, and Cell Permeability of **7y**, **8l**, and **9**

compound	**7y**	**8l**	**9a**	**9b**	**9c**	*d*_2_-**8l**
Notum IC_50_ (nM)^a^	9.5 ± 0.2	6.7 ± 1.6	1300 ± 210	140 ± 20	7.5 ± 1.6	4.6 ± 1.4
aq. solubility (μg/mL)/(μM)	210/680	68/240	ND^b^	ND	400/1400	44/155
MLM/HLM Cl_i_ (μL/min/mg protein)	6.7/1.7	3.2/1.5	ND	ND	4.9/ND	5.4/ND
MDCK-MDR1						
AB/BA *P*_app_ (×10^–6^ cm s^–1^)	46/28	31/39	ND	ND	2/5	48/35
ER	0.61	1.25			2.5	0.73

a All values are mean ± s.d. of *n* = 3–6 experiments quoted to 2 s.f.

b ND, not determined.

As before, trusted design metrics were calculated to aid compound design and assess progress, supported by structure-based drug design (SBDD) through inhibitor docking and X-ray structures. However, a significant change in our strategy was to discount any weakly acidic functional groups or heterocycles as these had failed to give sufficient brain penetration in mouse PK experiments (e.g., **4** and **5**).^26,27^ Instead, lead compounds would need to be non-ionized, or weakly basic, at physiologically relevant pH to be advanced through the screening sequence.

**Scheme 1:**
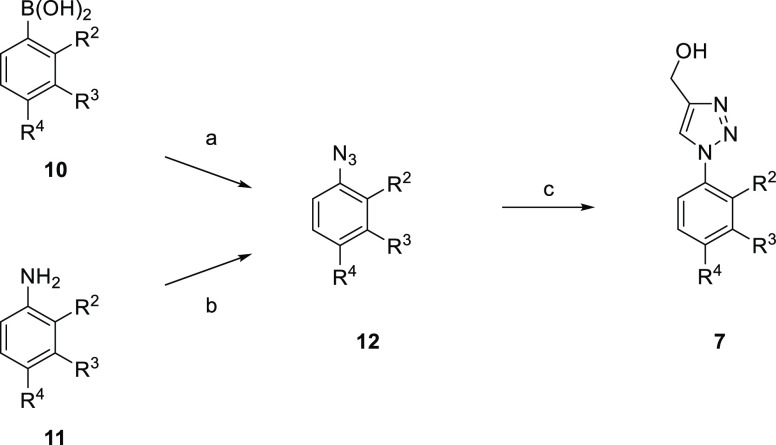
Preparation of (1-Phenyl-1*H*-1,2,3-triazol-4-yl)methanols **7** Representative reagents and conditions: (a) NaN_3_ (2.0 equiv), CuSO_4_·5H_2_O (0.2 equiv), MeOH, 40 °C, 5 h; (b) (i) NaNO_2_ (1.2 equiv), CF_3_CO_2_H, 0 °C → RT, 1.5 h; (ii) H_2_O, RT → 0 °C, (iii) NaN_3_ (1.1 equiv), 0 °C → RT, 1 h; (c) HC≡CCH_2_OH (1.0 equiv), sodium l-ascorbate (0.4 equiv), CuSO_4_·5H_2_O (0.2 equiv), ^*t*^BuOH-H_2_O, 50 °C, 2 h.

**Scheme 2:**
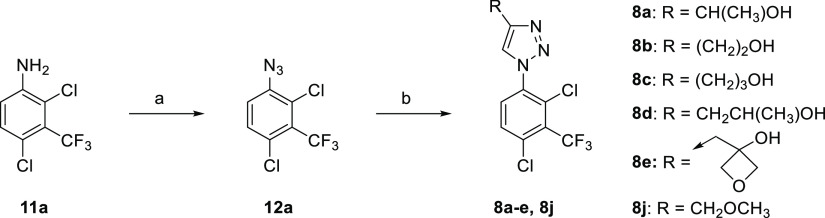
Preparation of 1,2,3-Triazoles **8a–e**, **8j** Representative reagents and conditions: (a) (i) NaNO_2_ (2.0 equiv), aq. HCl, MeCN, RT, 1 h; (ii) RT → 0 °C, NaN_3_ (2.0 equiv), 0 °C, 1 h; or (i) NaNO_2_ (1.2 equiv), CF_3_CO_2_H, 0 °C → RT, 1.5 h; (ii) RT → 0 °C, H_2_O, NaN_3_ (1.1 equiv), 0 °C → RT, 1 h; (b) RC≡CH (**13**) (1.0 equiv), sodium l-ascorbate (0.4 equiv), CuSO_4_·5H_2_O (0.2 equiv), MeOH-^*t*^BuOH-H_2_O, 40 °C, 16 h.

**Scheme 3:**
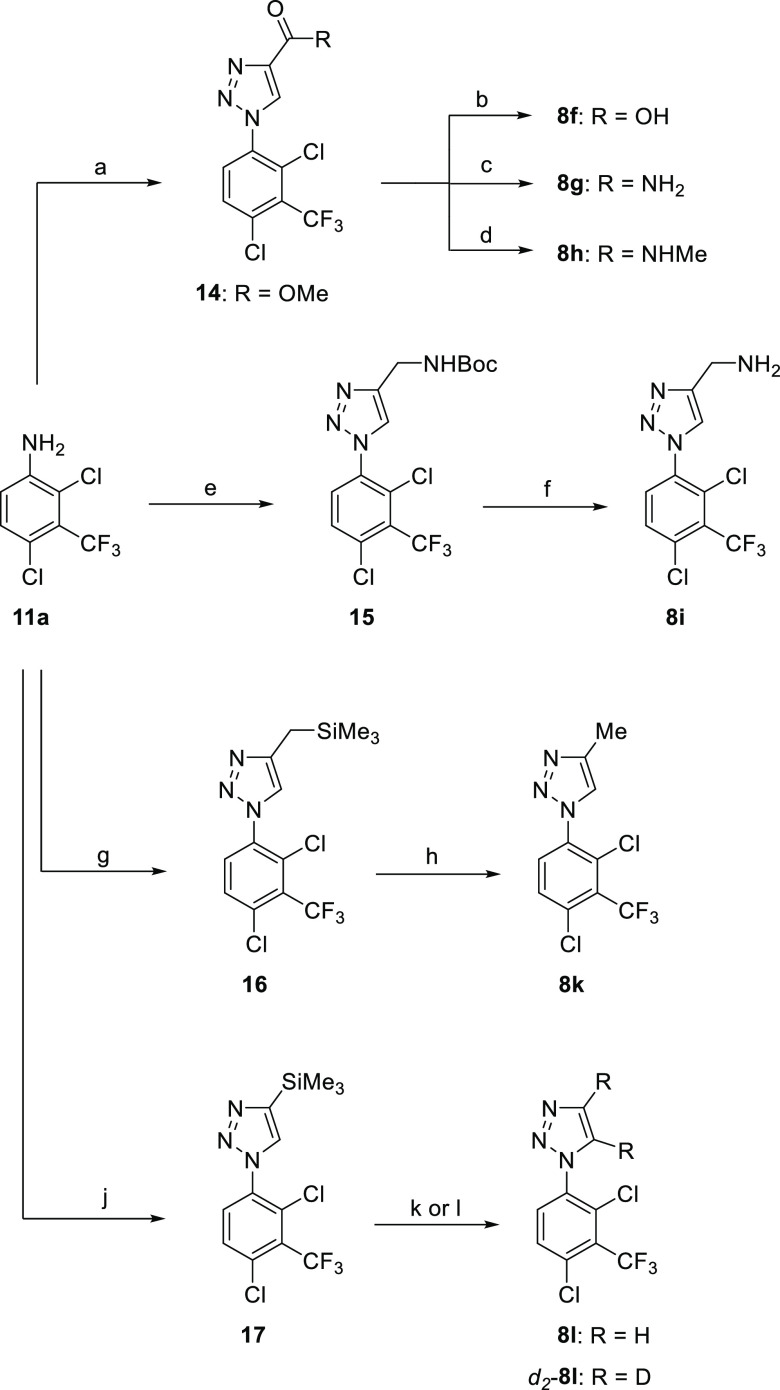
Preparation of 1,2,3-Triazoles **8f–i**, **8k–l** Reagents and conditions: (a) (i) NaNO_2_ (2.0 equiv), aq. HCl, MeCN, RT, 1 h; (ii) RT → 0 °C, NaN_3_ (2.0 equiv), 0 °C, 1 h; (iii) HC≡CCO_2_Me (1.0 equiv), sodium l-ascorbate (0.4 equiv), CuSO_4_·5H_2_O (0.2 equiv), MeOH-^*t*^BuOH-H_2_O, 40 °C, 16 h, 71%; (b) (i) aq. NaOH (2.0 equiv), MeOH, RT, 1 h; (ii) aq HCl, 95%; (c) NH_3_, MeOH, 70 °C, 3 h, 46%; (d) MeNH_2_, MeOH, 70 °C, 3 h, 64%; (e) NaNO_2_ (1.2 equiv), CF_3_CO_2_H, 0 °C → RT, 1.5 h; (ii) RT → 0 °C, aq. NaN_3_ (1.1 equiv), 0 °C → RT, 1 h; (iii) aq. NaOH; (iv) HC≡C-CH_2_NHBoc (1.0 equiv), sodium l-ascorbate (0.4 equiv), CuSO_4_·5H_2_O (0.2 equiv), ^*t*^BuOH-H_2_O, 50 °C, 16 h, 8%; (f) HCl (excess), dioxane, 50 °C, 16 h, quant.; (g) (i) NaNO_2_ (1.2 equiv), CF_3_CO_2_H, 0 °C → RT, 1.5 h; (ii) RT → 0 °C, H_2_O, NaN_3_ (1.1 equiv), 0 °C → RT, 1 h; (iii) sodium l-ascorbate (0.4 equiv), CuSO_4_·5H_2_O (0.2 equiv), HC≡CCH_2_SiMe_3_ (1.0 equiv), ^*t*^BuOH-H_2_O, 50 °C, 16 h; (h) (i) MeOH, K_2_CO_3_ (10 equiv), reflux, 2%; (ii) TBAF (2.0 equiv), THF, RT, 16 h; (j) (i) NaNO_2_ (4.7 equiv), CF_3_CO_2_H, 0 °C → RT, 1 h; (ii) RT → 0 °C, NaN_3_ (4.7 equiv), 0 °C → RT, 16 h; (iii) RT → 0 °C, aq. NaOH; (iv) HC≡CSiMe_3_ (2.0 equiv), sodium l-ascorbate (0.9 equiv), CuSO_4_·5H_2_O (0.5 equiv), MeOH-^*t*^BuOH-H_2_O, RT, 16 h; (k) MeOH, K_2_CO_3_ (10 equiv), RT, 24 h, 67%; (l) MeOH-*d*_4_, D_2_0, K_2_CO_3_ (3.0 equiv), RT, 3 days, 67%.

**Scheme 4:**
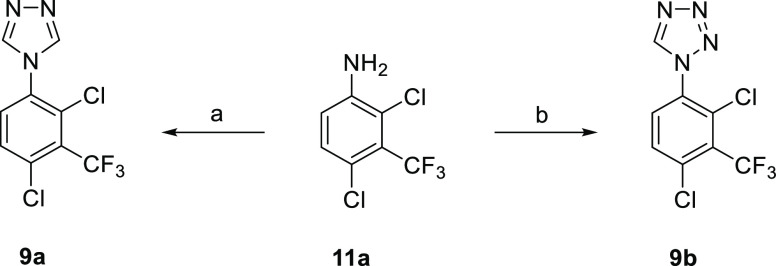
Preparation of 1,2,4-Triazole **9a** and 1,2,3,4-Tetrazole **9b** Reagents and conditions: (a) ClSiMe_3_ (15 equiv), 1,2-diformylhydrazine (3.0 equiv), Et_3_N, pyridine, 0 °C → 130 °C, 20 h, 50%; (b) HC(OMe)_3_ (3.0 equiv), NaN_3_ (3.1 equiv), AcOH, 100 °C, 1.5 h, 10%.

**Scheme 5:**
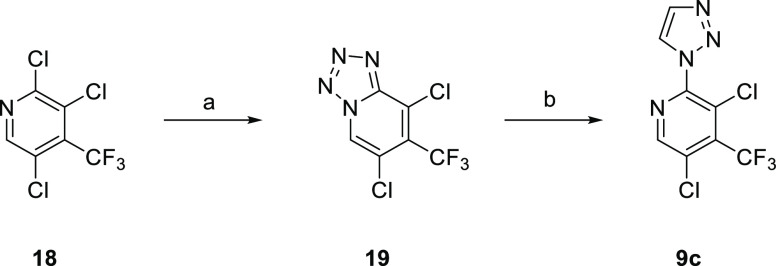
Preparation of (1,2,3-Triazol-1-yl)pyridine **9c** Reagents and conditions: (a) (i) NH_2_NH_2_·H_2_O (1.5 equiv), EtOH, 85 °C, 4 h; (ii) HCl, RT → 0 °C, NaNO_2_ (2.0 equiv), 30 min; (iii) NaOH, 0 °C → RT; (b) sodium l-ascorbate (1.0 equiv), CuSO_4_·5H_2_O (0.1 equiv), HC≡CSiMe_3_ (1.0 equiv), ^*t*^BuOH-H_2_O, 40 °C, 16 h, 2%.

General synthetic methods to prepare inhibitors **7–9** (Tables 1–3) are shown in Schemes 1–5. Our initial efforts focused on exploration of the aryl ring SAR, thus a reliable synthesis of substituted (1-aryl-1*H*-1,2,3-triazol-4-yl)methanols **7** was required (Scheme 1). Target compounds **7** were readily prepared in two steps from commercially available starting materials. Either the prerequisite boronic acid **10** or aniline **11** was converted to the required aryl azide **12** and then subsequent copper(I)-catalyzed azide–alkyne cycloaddition (CuAAC) with propargyl alcohol afforded the desired triazoles **7**.

This approach (**11** → **12** → **7**) proved to be versatile and reliable and was applied to the synthesis of a number of inhibitors. 2,4-Dichloro-3-(trifluoromethyl)aniline (**11a**) was converted to the corresponding azide **12a**, and reaction with the appropriate 1-alkyne **13** under CuAAC conditions gave a variety of 4-substituted 1,2,3-triazoles (**8**, **14–17**) (Schemes 2 and 3). A number of inhibitors were prepared by functional group conversion and/or protecting group strategies. Methyl ester **14** was converted to the acid **8f** by base hydrolysis, and to amides **8g** and **8h** by amination with NH_3_ and MeNH_2_, respectively. Acid deprotection of the Boc group from **15** gave methyl amine **8i**, and K_2_CO_3_/TBAF deprotection deprotection of **16** furnished 4-methyl triazole **8k**. CuAAC-promoted [2 + 3] cyclization of **12a** with Me_3_SiC≡CH gave the corresponding 4-TMS triazole **17**, and desilylation with K_2_CO_3_/MeOH afforded **8l**. Desilylation of **17** with CD_3_OD and D_2_O as the solvents promoted deuteration at the C4 and C5 positions of the triazole and afforded *d*_2_-**8l** with high-isotope incorporation.

Alternative heterocycles were prepared by standard methods (Scheme 4). Condensation of aniline **11a** with 1,2-diformylhydrazine gave 1,2,4-triazole **9a**, and the reaction of **11a** with trimethyl orthoformate and NaN_3_ gave 1,2,3,4-tetrazole **9b**. 2-Chloropyridine **18** was sequentially treated with hydrazine and NaNO_2_/HCl to give **19**, and then CuAAC with HC≡CSiMe_3_ afforded 2-triazolepyridine **9c** (Scheme 5). Tetrazolo[1,5-*a*]pyridine **19** acts as a synthon for the 2-azidopyridine in this reaction.

We first explored the SAR of the aryl ring with a variety of single substituents at the ortho-, meta-, and para-positions. The unsubstituted phenyl **7a** (IC_50_ 110 μM) was prepared and screened as a benchmark to assess these changes, along with the original hits **6a** and **6b**. Substitutions were biased toward small, lipophilic groups, based upon the available structural information, and building upon previous Notum SAR from phenyl-azole scaffolds.^25,26^ The tactic of walking single Cl, Me, and CF_3_ groups around the phenyl ring proved to be fruitful, with the 3-Me (**7e**), 3-Cl (**7f**), 3-CF_3_ (**7g**), 4-Me (**7k**), and 4-CF_3_ (**7l**) analogues affording the largest improvements in potency over **7a** (ca. 10 to 48-fold). A 2-Cl group (**7c**) afforded a modest increase in potency showing the potential benefit of substitution at this position. Inhibitors **7f**, **7g**, and **7l** were superior to hit **6a**.

Additive effects from multiple substituents were then investigated by preparing a set of dichlorophenyl triazole-matched molecular pairs (**7q–t**). This set of inhibitors was prioritized due to their synthetic accessibility from available commercial reagents and the precedent from **6b**. The original hit **6b** was preferred over all other isomers with activity following the order: 3,4-Cl_2_ (**6b**, IC_50_ 0.5 μM) > 3,5-Cl_2_ (**7t**, IC_50_ 2.4 μM) > 2,3-Cl_2_ (**7q**, IC_50_ 5.1 μM) ≈ 2,4-Cl_2_ (**7r**, IC_50_ 5.6 μM) > 2,5-Cl_2_ (**7s**, IC_50_ 23 μM). Improvements could be achieved with alternative 3,4-disubstitutions when combinations of preferred substituents (Me, Cl, and CF_3_) were used with a clear preference for 3-CF_3_-4-Cl (**7w**, IC_50_ 0.065 μM). Addition of a 2-Cl as a third substituent onto **7w** as 2-Cl-3-CF_3_-4-Cl (**7y**) gave a further boost in potency with **7y** (IC_50_ 0.0095 μM) being 50-fold more active than hit **6b** and 10,000-fold more active than the unsubstituted phenyl ring **7a**.

At this point in the program, mouse PK data for **7y** was generated in vivo to determine the extent of plasma and brain exposure and to verify the correlation of the in vitro ADME data with in vivo outcomes in this series. Triazole **7y** was selected as it combined excellent Notum activity (IC_50_ 9.5 nM) with an attractive ADME profile as it possessed good aqueous solubility, microsomal stability, and cell permeability (Table 1 and Supporting Information). Following a single oral dose of 10 mg/kg, plasma exposure for **7y** was low (*C*_max_ 300 ng/mL; AUC_(0→inf)_ 150 ng h/mL), which we attributed to higher clearance than predicted by MLM and highlighted the need to further improve metabolic stability (Figure S2). Our hypothesis for this disconnection between MLM stability and plasma clearance of **7y** was due to phase 2 metabolism/conjugation of the OH group, and this was subsequently supported by the higher clearance of **7y** in mouse hepatocytes (Cl_i_ 48 μL/min/10^6^ cells). Hence, we designed inhibitors that explored direct modification and/or replacement of the −CH_2_OH (Table 2). Despite the poor plasma exposures, **7y** displayed the highest brain-to-plasma ratio [*K*_p_ 1.2, based on AUC_0–inf_ (total drug)] observed to date and was far superior to previous leads **1** (*K*_p_ ca. 0),^28^**3** (*K*_p_ 0.29),^24^**4** (*K*_p_ 0.16),^26^ and **5** (*K*_p_ ca. 0)^27^ where comparable studies were performed. This was viewed to be a significant advantage for this series.

SAR of the C4-substituent of the triazole was studied with retention of the preferred aryl ring of **7y** as this group conferred significant activity to the series and was superior to all other substitution patterns explored so far (Table 2). Substitution at the C5 position was not investigated as previous studies with related 4-Cl-phenyl azoles showed this to be unfavorable.^26^

Modification of the −CH_2_OH through addition of the α-Me group (**8a**), elongation of the carbon chain (**8b–8c**), or a combination of both these approaches (**8d–8e**) leads to a dramatic reduction in potency (IC_50_ > 1 μM). Oxidation to the carboxylic acid **8f** was tolerated with a modest reduction in potency (**7y** vs **8f**), whereas activity was significantly diminished upon conversion to the corresponding primary **8g** and secondary amides **8h**. Primary amine **8i** also had a significant reduction in potency as did “capping” the −CH_2_OH as the −CH_2_OCH_3_ ether **8j**. Deletion of the −OH from **7y** to give the 4-Me triazole **8k** had a more modest effect upon the reduction in potency (12-fold) that was consistent with the loss of one H-bonding group from the ligand. So far, all changes to the C4 substituent had been unfavorable, then, somewhat unexpected, potent inhibition of Notum activity was entirely recovered by removal of the substituent as unsubstituted triazole **8l** (IC_50_ 6.7 nM) was the most potent inhibitor prepared in this series. Finally, revisiting the aryl ring substitution with 2,3,4-Cl_3_**8m**, as a simpler and direct analogue of **8l**, was slightly inferior.

**Figure 5:**
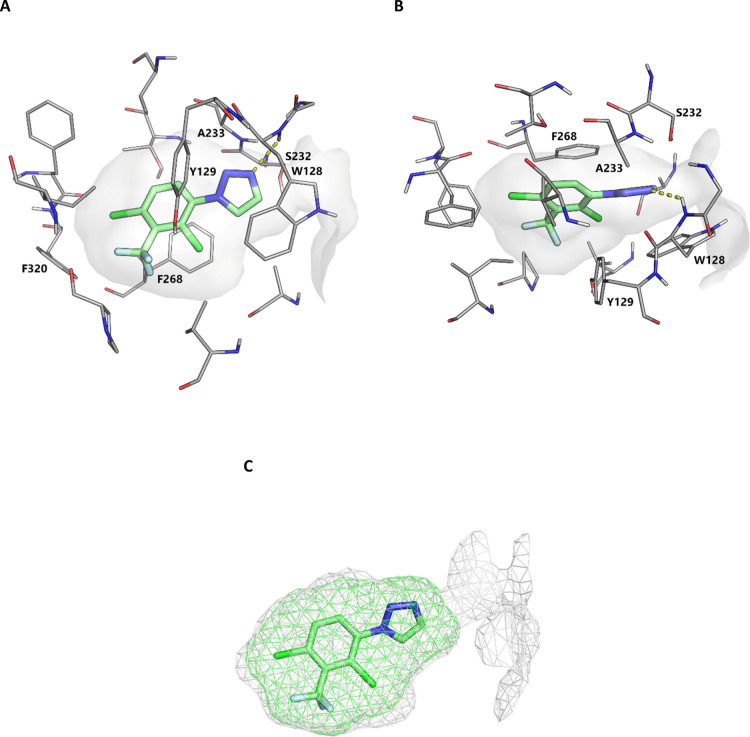
(A) Side view of structure of **8l** (PDB: 7PK3). (B) Top view of the structure of **8l** (PDB: 7PK3). The compound forms a hydrogen bond with the backbone of Trp128 (dashed line, 2.47 Å) and displays excellent occupancy of the lipophilic pocket. Select binding site residues shown within 4 Å of the compound. Water molecules have been removed for clarity. The surface of the Notum binding pocket outlined (gray). (C) Illustration of space occupied by **8l** (green mesh, PDB: 7PK3) and the outline of the pocket (gray mesh, PDB: 7PK3).

The Notum-**8l** X-ray structure is consistent with the ligand retaining the H-bond between N3 of the triazole and the backbone of Trp128. In addition, **8l** has near complete occupancy of the palmitoleate pocket through the 2-Cl-3-CF_3_-4-Cl substituents on the aryl ring. The near optimal filling of the pocket is achieved while still positioning the triazole in a favorable position for a hydrogen bond with the backbone of Trp128 (Figure 5). This combination of features is likely why such high potency is observed.

1,2,3-Triazole **8l** was superior in potency to the isomeric 1,3,4-triazole **9a** or the 1,2,3,4-tetrazole **9b** (Table 3). These effects seem to be quite subtle as most key inhibitor–Notum interactions are available with these alternative 1-aryl azoles and may be due to a more optimal electronic interaction of **8l** with Trp128 or simply that an N4 atom is detrimental.^49^ Modification of the phenyl ring of **8l** to the corresponding pyridine **9c** was equipotent and demonstrated that some modest polarity could be accommodated in the palmitoleate pocket. Dual deuteration of **8l** at the C4 and C5 positions of the triazole gave *d*_2_-**8l** as a tool to investigate the metabolic fate of the parent molecule.

Triazole **8l** has physicochemical and molecular properties consistent with drug-like space.^50^ It was noteworthy that **8l** had a much lower measured distribution coefficient (LogD_7.4_ 1.3 ± 0.05) than had been predicted by the calculated partition coefficient (*c*log*P* 3.9) despite being non-ionized at physiologically relevant pH. This disconnection could be attributed to the close proximity of the 2-Cl, 3-CF_3_, and 4-Cl substituents on the aromatic ring that results in a reduction in overall hydrophobic surface area when compared to summing their individual lipophilicity fragmental constants (π). These results highlight the importance of generating empirical data to correlate with calculated values. Evaluation of **8l** against standard design metrics using Notum inhibition from the biochemical assay (IC_50_ 6.7 nM, pIC_50_ 8.2) and measured lipophilicity (LogD_7.4_ 1.3) calculates **8l** to have high LE (0.67), LLE (6.9), and a favorable prediction of brain penetration (CNS MPO 5.4/6.0; BBB Score 5.6/6.0).

Triazole **8l** displayed satisfactory aqueous solubility in phosphate-buffered saline (PBS), good metabolic stability in both MLM and HLM, and high permeability in the MDCK-MDR1 cell transit assay with minimal evidence for recognition and efflux by the P-gp transporter (Table 3 and Supporting Information). In contrast, pyridine **9c** performed poorly in the permeability assay although these results were confounded by low compound recovery. Comparison of MLM stability of **8l** with *d*_2_-**8l** (Cl_i_ 3.2 vs 5.4 μL/min/mg protein) showed deuteration of the triazole afforded no improvement suggesting that CYP-mediated metabolism of the triazole C–H bonds was not a major route of clearance in this system.

Based on these data, **8l** was selected as our nominated lead from this series for further profiling as it had the best combination of Notum activity and in vitro ADME properties including lipophilicity.

Lead **8l** was then evaluated in additional in vitro pharmacology and ADME screens (Table 5). Triazole **8l** restored Wnt signaling in the presence of Notum (EC_50_ 110 nM; *n* = 4) in a in a cell-based TCF/LEF (Luciferase) reporter assay^23,26,27^ and gave standard S-shaped concentration–response curves up to 10 μM (Figure S9). Performing these experiments in the absence of Notum showed a maximal Wnt response at all concentrations tested (up to 10 μM; *n* = 4). The activation of Wnt signaling was due to direct on-target inhibition of Notum by **8l** and not by assay interference or cell toxicity (up to 10 μM).

**Table 4:** Rodent Plasma and Brain PK Parameters of 8**l**^a^

	mouse^b^	rat^c^
Intravenous (i.v.)		
dose (mg/kg)	1.0^d^	1.0^d^
CL (mL/min/kg)	10.4	7.5
Vd_ss_ (L/kg)	2.0	2.0
*t*_1/2_ (h)	2.4	3.3

	mouse^b^		rat^c^	
	plasma	brain	plasma	brain
Oral (p.o.)				
dose (mg/kg)	10^e^		10^d^	
*t*_1/2_ (h)	2.4	2.2	4.7	8.9
*T*_max_ (h)	0.5	1.0	4.0	0.5
*C*_max_ (ng/mL or ng/g)	2310	2900	3260	3650
AUC_0–inf_ (ng·h/mL or ng·h/g)	10 800	11 600	32 000	46 100
*F*_o_ (%)	66		140	
brain-to-plasma ratio (*K*_p_)^f^	1.1		1.4	
free partition coefficient of brain (*K*_p,uu_)^f^	0.77		0.75	

a Values are mean; *n* = 3 per time point; terminal blood and brain levels were measured up to 24 h. All animals were normal throughout the study period.

b Male C57BL6 mice.

c Sprague–Dawley male rats.

d Formulation A: dimethylsulfoxide (DMSO) (2% v/v) + 10% v/v Solutol in 1× PBS (98% v/v).

e Formulation B: 0.1% Tween80 in water.

f *K*_p_ was calculated by the ratio of brain concentration–time profile (AUC_(0–inf),brain_) to that of plasma concentration–time profile (AUC_(0–inf),plasma_). *K*_p,uu_ was calculated by multiplying *K*_p_ with the ratio of free fraction in brain homogenate to plasma (*f*_u,brain_/*f*_u,plasma_).

**Table 5:**
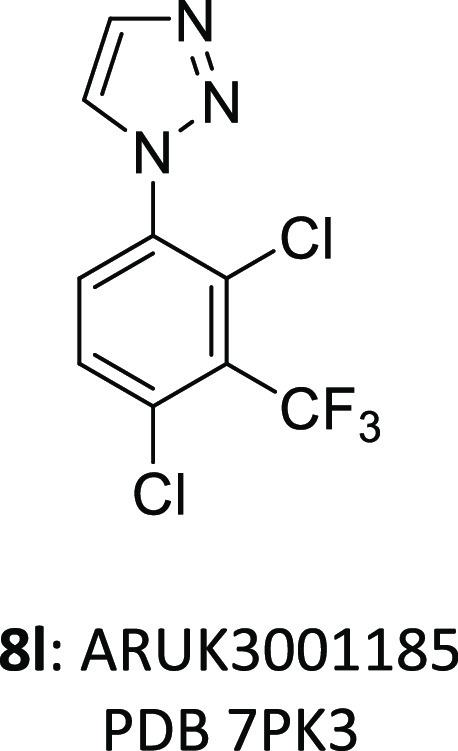

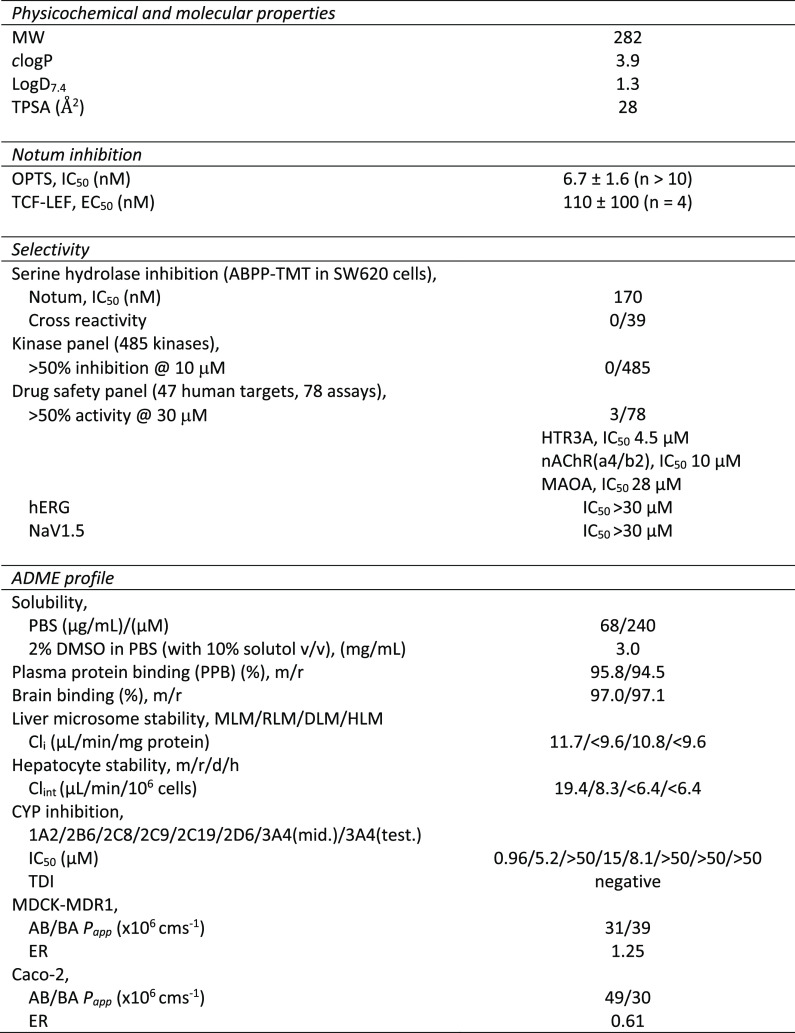
Extended Profile of **8l**^a^

a Non-standard abbreviations: m, mouse; r, rat; d, dog; h, human; mid., midazolam; and test., testosterone.

The aqueous solubility of **8l** could be significantly enhanced to 1 mg/mL with cosolvents bringing added flexibility to study design. Triazole **8l** has good stability in liver microsomes and hepatocytes across mouse, rat, dog, and human, which predicts for low clearance across these species. Screening in HLM including eight standard substrates of cytochrome P450 (CYP450) enzymes showed moderate/weak inhibition of 1A2 (IC_50_ 0.96 μM), 2B6 (IC_50_ 5.2 μM), 2C9 (IC_50_ 15 μM), and 2C19 (IC_50_ 8.1 μM) but not 2C8, 2D6, or 3A4 (IC_50_ > 50 μM). Furthermore, there was no evidence for CYP time-dependent inactivation (TDI) as assessed by the preincubation of HLM with **8l** in the presence and absence of NADPH, followed by the incubation with the discrete marker substrates (IC_50_ shift). There was good cell permeability across a Caco-2 monolayer consistent with good absorption from the gut. The hepatocyte data for **8l** was encouraging as this deletion of the −CH_2_OH group from **7y** seems to have removed the liability of this metabolic soft spot. Hence, **8l** was advanced to rodent PK studies.

PK data for **8l** was generated in vivo in mouse, and subsequently in rat, to evaluate plasma and brain exposure (Table 4 and Supporting Information). Following single intravenous administration of **8l** to mouse, plasma clearance was low compared to liver blood flow and volume of distribution was moderate resulting in an elimination half-life of 2.4 h (Figure S3, Table S11). Following a single oral dose to mouse, **8l** was rapidly absorbed and showed good oral bioavailability (66%) (Figure S4, Table S12). Good brain penetration was confirmed with drug levels in the brain similar to drug levels in the plasma (*K*_p_ 1.1). The brain-to-plasma ratio was 0.77 (*K*_p,uu_) when free drug concentrations were calculated. This oral dose of 10 mg/kg achieved a concentration in the brain of *C*_max_ ≈ 300 nM (free drug) that exceeded the Notum EC_50_ from the cell-based TCF/LEF assay, and brain levels were maintained above 100 nM for ∼4 h. Although encouraging, this dose, and the resulting level of brain exposure, may be insufficient to promote a sustained pharmacodynamic (PD) response, where Notum activity was reduced for an extended period of time. This will need to be determined empirically. Hence, we subsequently explored alternative dosing regimes to provide some flexibility in the determination of the required efficacious concentrations (*C*_eff_) in rodent models of disease, and its relationship to Notum pharmacology (EC_50_), that is, establish the PK–PD relationship (vide infra).

PK evaluation of **8l** in rat also showed low plasma clearance and moderate volume of distribution with a half-life of 3.3 h (Table 4). Oral administration to rat showed good absorption, drug levels, and brain penetration (*K*_p_ 1.4). The oral bioavailability was supramaximal with *F*_o_ 140%. The plasma (and brain) concentration–time plots clearly show double *C*_max_ peaks (Figure S7), which could indicate some contribution from excreted drug being reabsorbed from the gastrointestinal tract and/or an effect on gastric emptying times. On balance, these results established that **8l** has PK properties compatible with evaluation in rodent models of disease.

Triazole **8l** emerged as a promising lead with potent inhibition of Notum enzymic activity in both biochemical and cellular assays, along with excellent brain penetration in rodent. The wider pharmacology activity of **8l** was then assessed against serine hydrolases, kinases, and representative drug safety targets to identify any off-target activity and to establish its selectivity.

**Figure 6:**
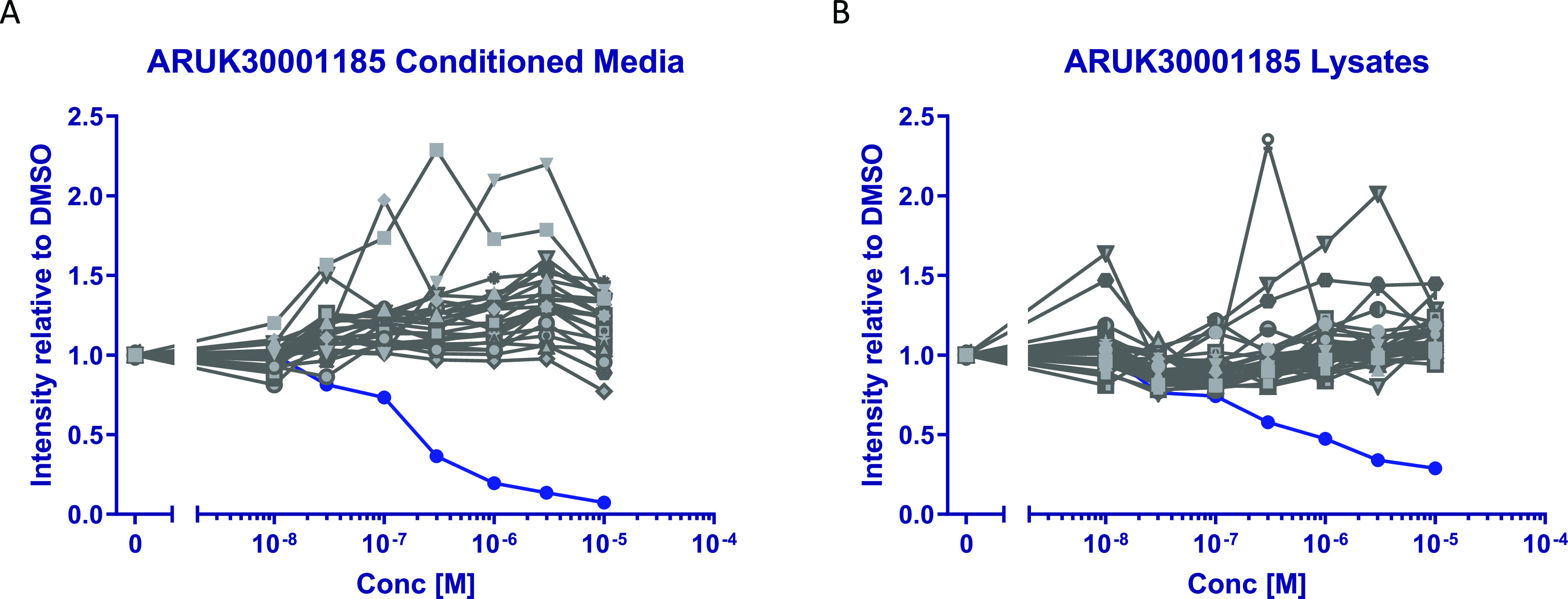
Concentration–response curves from proteomic analysis of **8l** in SW620 cells. The response for Notum is shown in dark blue circles (•) and all other serine hydrolases in gray. (A) Concentrated conditioned media, Notum IC_50_ 170 nM. (B) Cell lysates. Each sample set was preincubated with an eight-point concentration–response curve of **8l** or DMSO, then labeled for 20 min with FP-Biotin (2 μM) to capture active serine hydrolases. Labeled proteins were enriched on NeutrAvidin agarose and then digested using LysC and trypsin. The subsequent peptides were isobarically labeled with eight channels of a TMT10plex and combined for final LC–MS/MS analysis. The MS proteomics data have been deposited to the ProteomeXchange Consortium via the PRIDE partner repository with the dataset identifier PXD031338.

Selectivity against serine hydrolases was assessed by unbiased proteomic analysis in a human colorectal cancer cell line SW620 that has been characterized to have high Notum expression.^51^ Quantitative ABPP was performed using FP-biotin to label active serine hydrolases in concentrated conditioned media and cell lysates, combined with tandem mass tagging technology (TMT) for relative quantification.^52^ Triazole **8l** showed concentration-dependent inhibition of Notum activity (IC_50_ 170 nM in conditioned media) and demonstrated high selectivity in this system as there was no cross reactivity against any of the other 39 serine hydrolases detected (Figures 6, S11 and S12).^18,52b^

Compound **8l** showed excellent selectivity for Notum over a panel of 485 distinct kinases with <50% inhibition at 10 μM for all these kinases including casein kinase 1 (CK1) and GSK3 (Table 5). This was of critical importance to studies wishing to assess the function of Notum with **8l**, as CK1 and GSK3 phosphorylate β-catenin leading to its degradation and thus reducing Wnt signaling.^53^ For some context, **8l** showed the highest activity in this kinase panel at CDK14/cyclin Y with 43% inhibition at 10 μM (*n* = 2) (Table S20).

Compound **8l** was screened for off-target pharmacology across multiple drug target classes in the DiscoverX Safety47 panel. This suite of 78 functional assays assesses pharmacological modulation of 47 human off-target liabilities that have been highlighted for the initial safety assessment of lead compounds.^54^ There was minimal activity at 75/78 targets having IC_50_/EC_50_ > 30 μM with only modest activity at HTR3A (IC_50_ 4.5 μM), nAChR(a4/b2) (IC_50_ 10 μM), and MAOA (IC_50_ 28 μM) (Table 5). Liability through direct interaction with cardiac ion channels was low as **8l** had no significant effect on either the hERG or the cardiac sodium channels (IC_50_ > 30 μM) (Table S21).

Having established **8l** as a potent and selective inhibitor of Notum carboxylesterase activity, our efforts shifted to developing a multigram synthesis of **8l** and identification of a suitable dosing regimen to investigate the role of Notum in mouse models of neurodegenerative disease.

**Scheme 6:**
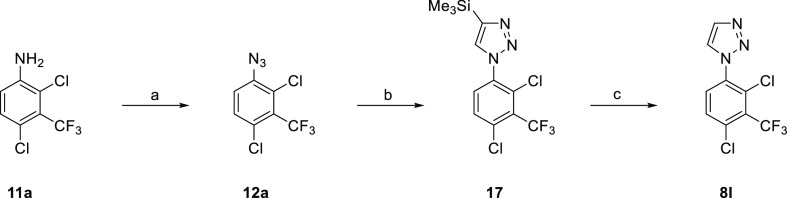
Multigram Synthesis of **8l** Reagents and conditions: (a) (i) NaNO_2_ (4.7 equiv), CF_3_CO_2_H, 0 °C, 0.5 h → RT, 1.5 h; (ii) NaN_3_ (4.7 equiv), H_2_O, 0 °C → RT, 16 h; (iii) NaOH, 0 °C; (b) HC≡CSiMe_3_ (2.0 equiv), sodium l-ascorbate (0.9 equiv), CuSO_4_·5H_2_O (0.5 equiv), MeOH-^*t*^BuOH-H_2_O, RT, 16 h; (c) K_2_CO_3_ (10 equiv), MeOH, RT, 24 h; overall yield, 67%.

The multigram synthesis of **8l** essentially followed the discovery route but with additional precautions to minimize the risk of high energy intermediates on scale such as the azide **12a** (Scheme 6). Differential scanning calorimetry (DSC) analysis of azide **12a** determined an exotherm onset temperature at 125.8 and so 42 °C was established as the maximum safe handling temperature (Figure S15). Reactions were performed in smaller batches and then pooled for purification to give a single, homogenous product.

Increasing the oral dose of **8l** to mouse from 10 mg/kg to twice daily dosing of 30 mg/kg (2 × 30 mg/kg, p.o. at *T* = 0 and 9 h, formulation A) achieved higher and prolonged drug concentrations in the plasma and brain (Figure S5, Table S13). The sustained free drug levels of **8l** in the brain now exceeded the Notum EC_50_ for ca. 20 h. This twice daily oral dosing of **8l** was well tolerated in both wild-type and aged APP/PS1 mice for 7 days and was therefore deemed suitable for the in vivo pathfinder experiments. These studies are ongoing and will be reported in due course.

## Conclusions

Crystallographic fragment screening of the DSPL for binding to Notum, supported by a biochemical enzyme assay to rank inhibition activity, proved to be successful in identifying chemically enabled hits. This screening strategy identified 58 hits that were shown to bind to the active site of Notum with 20 of these having IC_50_ < 100 μM. A pair of outstanding hits were 4-(hydroxymethyl)triazoles **6a** (IC_50_ 12 μM) and **6b** (IC_50_ 0.5 μM), and these were selected for fragment development to improve Notum activity and develop PK properties for oral dosing to achieve good plasma exposure and brain penetration.

Exploration of the substituents on the aryl ring that binds in the palmitoleate pocket of Notum gave potent early lead **7y** (IC_50_ 9.5 nM) and further optimization of the triazole group delivered **8l** (IC_50_ 6.7 nM). It is noteworthy that advanced lead **8l** can be characterized as a fragment-sized molecule (MW 282; HAC 17) with high LE and LLE, and that inhibition of Notum activity was increased by over 15,000-fold by optimization of the substituents on the aryl ring (**8l** vs **7a**). These findings highlight the benefits of FBDD when aligned with a suitable drug target.

The Notum-**8l** X-ray structure clearly showed that **8l** has near complete occupancy of the palmitoleate pocket through the 2-Cl-3-CF_3_-4-Cl substituents on the aryl ring. Triazole **8l** restored Wnt signaling in the presence of Notum (EC_50_ 110 nM) in a cell-based TCF/LEF reporter assay confirming functional activity. Assessment in broader off-target and safety pharmacology screens showed **8l** to be selective against serine hydrolases, kinases, and representative drug targets. PK studies with **8l** in mouse and rat showed good plasma exposure and brain penetration, and was well tolerated.

In summary, triazole **8l** (ARUK3001185) is a potent and selective inhibitor of Notum activity with good brain penetration suitable for oral dosing in rodent models of disease.

## Experimental Section

### Notum Protein Expression and Purification

Methods have been described in detail elsewhere.^25^

### Notum OPTS Biochemical Assay

Methods have been described in detail elsewhere.^23−26^ Representative concentration–response curves are provided in the Supporting Information.

### TCF/LEF Reporter (Luciferase) Cell-Based Assay

Methods have been described in detail elsewhere.^23,26,27^ Concentration–response curves are provided in the Supporting Information.

### Proteomic Analysis in SW620 Cells

#### Cell Culture

SW620 cells were grown in Leibovitz’s L-15 medium supplemented with 10% FBS, Pen/Strep 10 U/mL, and 0.075% sodium bicarbonate.

#### Conditioned Medium Generation

SW620 cells were grown to around 80% confluence, washed three times with PBS, and then placed in serum-free media for 48 h. The medium was then removed, spun at 300G to remove any solids, and then concentrated in 10k MWCO protein concentrators (Pierce) by centrifugation.

#### Cell Lysate Generation

SW620 cells were grown to around 80% confluence, the medium was removed and cells were washed three times with PBS, and then lysed in the ice cold IP lysis buffer (Pierce). Lysates were clarified by centrifugation at 15k G for 10 min at 4 °C, then protein quantification was performed by the BCA assay (Pierce) and lysates were equalized to 1.5 mg/mL.

#### Proteomics Protocol

Either lysates or conditioned medium was treated with the appropriate concentration of each inhibitor or DMSO across a range of concentrations and then equilibrated at RT for 45 min. Following this, 2 μM FP-Biotin (Santa Cruz) was added to each sample for 20 min with agitation. Lysates were then precipitated using chloroform–methanol, and the protein pellet was washed two times with MeOH. Protein pellets were dissolved in 50 mM HEPES pH 8.0 with 2% SDS and 10 mM DTT, then the SDS concentration was adjusted to 0.2% with further 50 mM HEPES pH 8.0. Each solution was then added to an aliquot of equilibrated NeutrAvidin beads,^55^ which had been washed two times with 50 mM HEPES pH 8.0 with 0.2% SDS, for 16 h at RT with agitation. Beads were then pelleted by centrifugation, the supernatant was removed and then washed three times with 50 mM HEPES pH 8.0 with 0.2% SDS, followed by three washes with 50 mM HEPES pH 8.0. The beads were then incubated with 10 mM DTT in 50 mM HEPES pH 8.0 for 15 min at RT, followed by 20 mM iodoacetamide, which was incubated for 30 min in the dark. A further aliquot of 10 mM DTT was added to the beads, then they were pelleted by centrifugation, the supernatant was removed, the beads were washed with 50 mM HEPES pH 8.0, and all the supernatants were removed. LysC (Wako) was dissolved in 50 mM HEPES pH 8.0 to give a 1 μg/mL solution, of which 50 μL (50 ng of LysC) was added to the beads and incubated overnight at 37 °C. The beads were pelleted, the supernatant was removed, and then washed with additional 50 mM HEPES pH 8.0 to extract as many peptides as possible. 75 ng of trypsin (Pierce) was added from a 20 ng/μL solution in 50 mM HEPES pH 8.0 and incubated at 37 °C for 7 h. Each set of conditions were then labeled using eight channels of a TMT10plex (Thermo) following the manufacturer’s instructions, then combined. TMT-labeled tryptic peptides were filtered using 1 mm Empore C18 solid-phase extraction discs aka “stage tips” (3M) prior to vacuum centrifugation and resuspension in 0.1% trifluoroacetic acid.

On an Ultimate 3000 nanoRSLC HPLC (Thermo Scientific), 1–10 μL of sample was loaded onto a 20 mm × 75 μm Pepmap C18 trap column (Thermo Scientific) prior to elution via a 50 cm × 75 μm EasySpray C18 column into a Lumos Tribrid Orbitrap mass spectrometer (Thermo Scientific). A 70′ gradient of 6–40% B was used, followed by washing and re-equilibration (A = 2% MeCN, 0.1% formic acid; B = 80% MeCN, 0.1% formic acid).

The Orbitrap was operated in “Data Dependent Acquisition” mode, followed by MS/MS in “TopS” mode, with Orbitrap accumulation at *R* = 50,000 of HCD fragmented parent ions.

Raw files were processed using MaxQuant (maxquant.org) and Perseus (maxquant.net/perseus) with a recent download of the uniprot homo sapiens reference proteome database and a common contaminants database. A decoy database of reversed sequences was used to filter false positives, at a peptide false detection rate of 1%.

Analysis was performed using Perseus and filtered against contaminants, reversed, and proteins identified by the site. Protein groups were then filtered by those which contained valid values in at least six of the eight conditions. Serine hydrolases were identified by filtering for those proteins identified as such in these two reference publications^18,52b^ with intensity values normalized relative to the DMSO control, and concentration–response curves and IC_50_ values were determined using GraphPad Prism 9.1.0.

The identity and quantification of each serine hydrolase identified in SW620 cells by FP-biotin ABPP for **8l** (and **1**) is provided in the Supporting Information. The MS proteomics data have been deposited to the ProteomeXchange Consortium via the PRIDE partner repository^56^ with the dataset identifier PXD031338.

### Structural Biology

The methods of protein production, crystallization, data collection, and structure determination have been described in detail.^26^ Data collection and refinement statistics are presented in the Supporting Information.

To prepare the figures of the crystal structures, the PDB entry was processed using the Protein Preparation Wizard in Schrödinger Maestro 12.4 using the default settings and optimizing the hydrogen bond assignment. No changes were made to the position of heavy atoms. Final images were prepared using PyMol version 2.4.

### ADME Assays

Molecular properties (MW, *c*log*P*, and TPSA) were calculated with ChemDraw Professional v16.0.1.4(77). Distribution coefficients (LogD_7.4_) were measured using the shake flask method.

Selected compounds were routinely screened for aqueous solubility in PBS (pH 7.4), transit performance in MDCK-MDR1 cell lines for permeability, and metabolic stability in MLM and HLM as a measure of clearance. Lead **8l** was also screened for inhibition of representative CYP450 enzymes, permeability across the Caco-2 cell monolayer, and metabolic stability in liver microsomes and hepatocytes. Assay protocols and additional data are presented in the Supporting Information. ADME studies reported in this work were independently performed by Cyprotex (Macclesfield, U.K), GVK Biosciences (Hyderabad, India), and WuXi AppTec (Shanghai, China).

### PK Studies

In vivo PK data in mouse and rat was independently generated at Charles River Laboratories (Groningen, Netherlands), GVK Biosciences (Hyderabad, India), and WuXi AppTec (Shanghai, China). PK was supported by the development of suitable formulations for the route of administration, and the measurement of plasma protein binding (PPB) and brain tissue binding for the calculation of free drug levels in these compartments. Study protocols and additional data are presented in the Supporting Information.

### Kinase Selectivity Panel

Kinase selectivity screening was performed by Thermo Fisher Scientific (Paisley, U.K.) in their SelectScreen Biochemical Kinase Profiling Service. Assay information and data are presented in the Supporting Information.

### Safety Pharmacology Studies

Screening for off-target pharmacology across multiple drug target classes was performed in the DiscoverX Safety47 panel as the SAFETYscan E/IC50 ELECT service by Eurofins (San Diego CA, U.S.A.). Assay information and data are presented in the Supporting Information.

### Chemistry

#### General Information

Methods have been described in detail elsewhere.^27a^ The purity of compounds **6–9** was evaluated by NMR spectroscopy and LCMS analysis. All compounds had purity ≥95% unless otherwise stated.

#### General Methods

*Caution* Azides are potentially explosive reagents and therefore all solutions containing azides were concentrated under reduced pressure behind a blast shield at RT. Furthermore, sodium azide reacts with acids to generate gaseous HN_3_, which is a toxic gas. All reactions were conducted behind a blast shield under a flow of nitrogen, and exit gases were passed through an aqueous base prior to their release into a ventilated fume hood.

#### Preparation of 1-Phenyl-1*H*-1,2,3-triazoles

##### General Method 1.1

*Caution* Step 1: Sodium azide (260 mg, 4.0 mmol, 2.0 equiv) and copper(II) sulfate pentahydrate (100 mg, 0.4 mmol, 0.2 equiv) were added to a solution of phenylboronic acid **10** (2.0 mmol, 1.0 equiv) in MeOH (10 mL). The reaction was stirred at 40 °C for 5 h, concentrated to ∼50% volume under reduced pressure, and then diluted with H_2_O (ca. 10 mL) and EtOAc (ca. 50 mL). The organic phase was separated, diluted with *t*-BuOH (1.7 mL), and then cautiously concentrated under reduced pressure at 25 °C to provide a solution of crude azide **12** in *t*-BuOH, which was used without further purification.

Step 2: Copper(II) sulfate pentahydrate (100 mg, 0.4 mmol, 0.22 equiv), sodium l-ascorbate (56 mg, 0.28 mmol, 0.15 equiv), H_2_O (1.7 mL), and propargyl alcohol (0.1 mL, 1.8 mmol, 1.0 equiv) were added to the solution of crude azide **12** from step 1. The reaction was stirred at 40 °C for 16 h, cooled to RT, diluted with H_2_O (ca. 10 mL), extracted with EtOAc (ca. 50 mL), and the organic phase was washed with H_2_O (ca. 10 mL) and brine (ca. 10 mL). The organic phase was dried, filtered, and concentrated under reduced pressure. The crude product was purified by column chromatography (0–5% MeOH in CH_2_Cl_2_) to give the triazoles **6b** and **7**.

##### General Method 1.2

*Caution* Step 1: Sodium nitrite (155 mg, 2.25 mmol, 1.2 equiv) was added portionwise to a solution of aniline **11** (1.87 mmol, 1.0 equiv) in TFA (2.0 mL) at 0 °C over 30 min. The reaction mixture was then allowed to warm to RT for 1.5 h before recooling to 0 °C. A solution of sodium azide (133 mg, 2.05 mmol, 1.1 equiv) in H_2_O (1.5 mL) was added dropwise over 5 min, stirred for 30 min, and then warmed to RT over 60 min. The mixture was basified to pH 8–9 by dropwise addition of aqueous NaOH (5 M), then diluted with *t*-BuOH (2.0 mL) to provide a solution of crude azide **12**, which was used without further purification.

Step 2: Copper(II)sulfate pentahydrate (93 mg, 0.37 mmol, 0.2 equiv), sodium l-ascorbate (148 mg, 0.75 mmol, 0.4 equiv), and mono-substituted alkyne **13** (1.87 mmol, 1.0 equiv) were added to the solution from step 1. The reaction mixture was stirred at 50 °C for 16 h, cooled to RT, diluted with brine (ca. 10 mL), and then extracted with EtOAc (ca. 50 mL). The organic phase was dried, filtered, and concentrated under reduced pressure. The crude product was purified by column chromatography (0–5% MeOH in CH_2_Cl_2_) to give triazoles **7** and **8**.

##### General Method 1.3

*Caution* Step 1: Aniline **11** (0.65 mmol, 1.0 equiv) was dissolved in H_2_O (0.5 mL) and MeCN (3.0 mL), then aqueous HCl (37%, 7.3 mL) was added at RT and stirred vigorously for 10 min. Sodium nitrite (90 mg, 1.3 mmol, 2.0 equiv) was added portionwise and the solution was stirred for 1 h, cooled to 0 °C, and then sodium azide (85 mg, 1.3 mmol, 2.0 equiv) was added portionwise. After 1 h, the solution was diluted with H_2_O (ca. 10 mL) and Et_2_O (ca. 50 mL). The organic phase was separated, diluted with *t*-BuOH (2.0 mL), and then cautiously concentrated under reduced pressure at 25 °C to provide a solution of crude azide **12** in *t*-BuOH, which was used without further purification.

Step 2: Sodium l-ascorbate (52 mg, 0.26 mmol, 0.4 equiv), mono-substituted alkyne **13** (0.65 mmol, 1.0 equiv), copper(II) sulfate pentahydrate (33 mg, 0.13 mmol, 0.2 equiv), MeOH (1.0 mL), and H_2_O (2.0 mL) were added to the solution of crude azide **12** from step 1, and the reaction was stirred at 40 °C for 16 h. The reaction was cooled to RT, diluted with H_2_O (ca. 10 mL), extracted with EtOAc (ca. 50 mL), and the organic phase was washed with H_2_O (ca. 10 mL) and brine (ca. 10 mL). The organic phase was dried, filtered, and concentrated under reduced pressure. The crude product was purified by column chromatography (0–5% MeOH in CH_2_Cl_2_) to give triazoles **7** and **8**.

##### General Method 1.4

*Caution* Step 1: Sodium nitrite (72 mg, 1.0 mmol, 1.2 equiv) was added portionwise to a solution of aniline **11** (0.9 mmol, 1.0 equiv) in TFA (0.5 mL) at 0 °C over 30 min. The reaction mixture was then allowed to warm to RT for 1.5 h before recooling to 0 °C. H_2_O (0.1 mL), sodium azide (62 mg, 1.0 mmol, 1.1 equiv) was added portionwise over 30 min, and then warmed to RT over 1 h. The mixture was basified to pH 8–9 by dropwise addition of saturated aqueous NaHCO_3_, then diluted with CH_2_Cl_2_ (ca. 50 mL). The organic phase was separated, diluted with *t*-BuOH (0.5 mL), and then cautiously concentrated under reduced pressure at 25 °C to provide a solution of crude azide **12** in *t*-BuOH, which was used without further purification.

Step 2: Copper(II) sulfate pentahydrate (43 mg, 0.2 mmol, 0.2 equiv), sodium l-ascorbate (69 mg, 0.4 mmol, 0.4 equiv), H_2_O (0.5 mL), and mono-substituted alkyne **13** (0.9 mmol, 1.0 equiv) were added to the solution of crude azide **12** from step 1, and the reaction mixture was stirred at 50 °C for 2–16 h. The reaction was cooled to RT, diluted with H_2_O (ca. 10 mL), extracted with EtOAc (ca. 50 mL), and the organic phase was washed with H_2_O (ca. 10 mL) and brine (ca. 10 mL). The organic phase was dried, filtered, and concentrated under reduced pressure. The crude product was purified by column chromatography (0–5% MeOH in CH_2_Cl_2_) to give triazoles **7** and **8**.

#### Notum Inhibitors

##### ((4-Chlorophenyl)-1*H*-1,2,3-triazol-4-yl)methanol (**6a**)

Purchased from Key Organics, 4F-329S.

##### ((3,4-Dichlorophenyl)-1*H*-1,2,3-triazol-4-yl)methanol (**6b**)

Purchased from Key Organics (4F-359S). It was prepared by General Method 1.1 from 3,4-dichlorophenylboronic acid and isolated as an off-white solid (110 mg, 11%).

^1^H NMR (600 MHz, DMSO-*d*_6_): δ 8.79 (s, 1H), 8.27 (d, *J* = 2.5 Hz, 1H), 7.97 (dd, *J* = 8.8, 2.5 Hz, 1H), 7.87 (d, *J* = 8.8 Hz, 1H), 5.36 (t, *J* = 5.6 Hz, 1H), 4.61 (d, *J* = 5.5 Hz, 2H); ^13^C NMR (151 MHz, DMSO-*d*_6_): δ 149.5, 136.3, 132.4, 131.8, 130.8, 121.6, 121.3, 119.9, 54.9; LCMS *m*/*z*: 244.1 [M + H]^+^.

##### (1-Phenyl-1*H*-1,2,3-triazol-4-yl)methanol (**7a**)

Prepared by General Method 1.1 from phenylboronic acid and isolated as an off-white solid (98 mg, 28%).

^1^H NMR (700 MHz, DMSO-*d*_6_): δ 8.68 (s, 1H), 7.96–7.84 (m, 2H), 7.64–7.54 (m, 2H), 7.56–7.41 (m, 1H), 5.32 (t, *J* = 5.6 Hz, 1H), 4.61 (dd, *J* = 5.6, 0.6 Hz, 2H); ^13^C NMR (176 MHz, DMSO-*d*_6_): δ 149.1, 136.8, 129.9, 128.5, 121.0, 119.9, 55.0; LCMS *m*/*z*: 176.1 [M + H]^+^.

##### ((2-Fluorophenyl)-1*H*-1,2,3-triazol-4-yl)methanol (**7b**)

Prepared by General Method 1.2 from 2-fluoroaniline and propargyl alcohol and isolated as a light brown oil (48 mg, 12%).

^1^H NMR (400 MHz, methanol-*d*_4_): δ 8.31 (d, *J* = 2.4 Hz, 1H), 7.89–7.77 (m, 1H), 7.63–7.51 (m, 1H), 7.47–7.35 (m, 2H), 4.80–4.78 (m, 2H); LCMS *m*/*z*: 194.1 [M + H]^+^.

##### ((2-Chlorophenyl)-1*H*-1,2,3-triazol-4-yl)methanol (**7c**)

Prepared by General Method 1.1 from 2-chlorophenylboronic acid and isolated as an off-white solid (30 mg, 7%).

^1^H NMR (600 MHz, DMSO-*d*_6_): δ 8.41 (s, 1H), 7.77 (dd, *J* = 8.0, 1.3 Hz, 1H), 7.67–7.56 (m, 3H), 5.35 (t, *J* = 5.6 Hz, 1H), 4.62 (d, *J* = 5.6 Hz, 2H); ^13^C NMR (151 MHz, DMSO-*d*_6_): δ 148.1, 134.7, 131.6, 130.6, 128.5, 128.5, 128.4, 125.0, 54.9; LCMS *m*/*z*: 210.1 [M + H]^+^.

##### ((3-Fluorophenyl)-1*H*-1,2,3-triazol-4-yl)methanol (**7d**)

Prepared by General Method 1.2 from 3-fluoroaniline and propargyl alcohol and isolated as a colorless oil (12 mg, 3%).

^1^H NMR (700 MHz, DMSO-*d*_6_): δ 8.75 (s, 1H), 7.89–7.74 (m, 2H), 7.69–7.53 (m, 1H), 7.41–7.24 (m, 1H), 5.36 (t, *J* = 5.6 Hz, 1H), 4.61 (dd, *J* = 5.6, 0.6 Hz, 2H); ^13^C NMR (176 MHz, DMSO-*d*_6_): δ 162.4 (d, *J* = 245.0 Hz), 149.3, 138.0 (d, *J* = 10.5 Hz), 131.8 (d, *J* = 9.2 Hz), 121.2, 115.8 (d, *J* = 3.0 Hz), 115.2 (d, *J* = 21.0 Hz), 107.4 (d, *J* = 26.5 Hz), 54.9; LCMS *m*/*z*: 194.1 [M + H]^+^.

##### ((3-Methylphenyl)-1*H*-1,2,3-triazol-4-yl)methanol (**7e**)

Prepared by General Method 1.2 from 3-methylaniline and propargyl alcohol and isolated as a clear colorless oil (7 mg, 2%).

^1^H NMR (400 MHz, methanol-*d*_4_): δ 8.43 (s, 1H), 7.70–7.67 (m, 1H), 7.65–7.60 (m, 1H), 7.46 (apparent t, *J* = 7.8 Hz, 1H), 7.35–7.30 (m, 1H), 4.78–4.75 (m, 2H), 2.47 (s, 3H); LCMS *m*/*z*: 190.2 [M + H]^+^.

##### ((3-Chlorophenyl)-1*H*-1,2,3-triazol-4-yl)methanol (**7f**)

Prepared by General Method 1.1 from 3-chlorophenylboronic acid and isolated as an off-white solid (115 mg, 27%).

^1^H NMR (600 MHz, DMSO-*d*_6_): δ 8.77 (s, 1H), 8.05 (t, *J* = 2.0 Hz, 1H), 7.93 (ddd, *J* = 8.1, 2.1, 0.9 Hz, 1H), 7.62 (t, *J* = 8.1 Hz, 1H), 7.55 (ddd, *J* = 8.1, 2.0, 0.9 Hz, 1H), 5.34 (t, *J* = 5.6 Hz, 1H), 4.61 (d, *J* = 5.6 Hz, 2H); ^13^C NMR (151 MHz, DMSO-*d*_6_): δ 149.4, 137.8, 134.2, 131.7, 128.3, 121.2, 119.7, 118.5, 54.9; LCMS *m*/*z*: 210.1 [M + H]^+^.

##### ((3-Trifluoromethylphenyl)-1*H*-1,2,3-triazol-4-yl)methanol (**7g**)

Prepared by General Method 1.2 from 3-trifluoromethylaniline and propargyl alcohol and isolated as a tan solid (175 mg, 39%).

^1^H NMR (700 MHz, DMSO-*d*_6_): δ 8.88 (s, 1H), 8.33–8.16 (m, 2H), 7.87–7.76 (m, 2H), 5.37 (t, *J* = 5.6 Hz, 1H), 4.63 (dd, *J* = 5.6, 0.6 Hz, 2H); ^13^C NMR (176 MHz, DMSO-*d*_6_): δ 149.5, 137.2, 131.3, 130.6 (q, *J* = 32.5 Hz), 125.0 (q, *J* = 3.7 Hz), 123.8, 123.6 (d, *J* = 272.6 Hz), 121.3, 116.5 (q, *J* = 3.9 Hz), 54.9; LCMS *m*/*z*: 244.2 [M + H]^+^.

##### ((3-Cyanophenyl)-1*H*-1,2,3-triazol-4-yl)methanol (**7h**)

Prepared by General Method 1.1 from 3-cyanophenylboronic acid and isolated as an off-white solid (100 mg, 25%).

^1^H NMR (600 MHz, DMSO-*d*_6_): δ 8.80 (s, 1H), 8.48–8.38 (m, 1H), 8.30 (ddd, *J* = 8.3, 2.2, 0.8 Hz, 1H), 7.95 (dd, *J* = 6.7, 1.1 Hz, 1H), 7.81 (t, *J* = 8.0 Hz, 1H), 5.38 (t, *J* = 5.6 Hz, 1H), 4.63 (d, *J* = 5.5 Hz, 2H); ^13^C NMR (151 MHz, DMSO-*d*_6_): δ 149.6, 137.2, 132.1, 131.4, 124.6, 123.3, 121.3, 117.9, 112.8, 55.0; LCMS *m*/*z*: 201.1 [M + H]^+^.

##### ((3-Ethoxyphenyl)-1*H*-1,2,3-triazol-4-yl)methanol (**7i**)

Prepared by General Method 1.1 from 3-ethoxyphenylboronic acid and isolated as an off-white solid (212 mg, 48%).

^1^H NMR (600 MHz, DMSO-*d*_6_): δ 8.70 (s, 1H), 7.52–7.41 (m, 3H), 7.09–6.95 (m, 1H), 5.31 (t, *J* = 5.6 Hz, 1H), 4.60 (d, *J* = 5.5 Hz, 2H), 4.13 (q, *J* = 7.0 Hz, 2H), 1.36 (t, *J* = 7.0 Hz, 3H); ^13^C NMR (151 MHz, DMSO-*d*_6_): δ 159.5, 149.1, 137.8, 130.9, 121.1, 114.6, 111.8, 105.9, 63.6, 55.0, 14.6; LCMS *m*/*z*: 220.2 [M + H]^+^.

##### ((4-Fluorophenyl)-1*H*-1,2,3-triazol-4-yl)methanol (**7j**)

Prepared by General Method 1.2 from 4-fluoroaniline and propargyl alcohol and isolated as an off-white solid (115 mg, 32%).

^1^H NMR (700 MHz, DMSO-*d*_6_): δ 8.65 (s, 1H), 7.97–7.88 (m, 2H), 7.47–7.36 (m, 2H), 5.32 (t, *J* = 5.6 Hz, 1H), 4.60 (dd, *J* = 5.6, 0.6 Hz, 2H); ^13^C NMR (176 MHz, DMSO-*d*_6_): δ 161.5 (d, *J* = 245.5 Hz), 149.2, 133.3 (d, *J* = 2.8 Hz), 122.3 (d, *J* = 8.7 Hz), 121.2, 116.7 (d, *J* = 23.2 Hz), 55.0 (s); LCMS *m*/*z*: 194.2 [M + H]^+^.

##### ((4-Methylphenyl)-1*H*-1,2,3-triazol-4-yl)methanol (**7k**)

Prepared by General Method 1.2 from 4-methylaniline and propargyl alcohol and isolated as a white solid (120 mg, 34%).

^1^H NMR (700 MHz, DMSO-*d*_6_): δ 8.61 (s, 1H), 7.80–7.73 (m, 2H), 7.37 (dd, *J* = 8.6, 0.6 Hz, 2H), 5.29 (t, *J* = 5.6 Hz, 1H), 4.62–4.54 (m, 2H), 2.36 (s, 3H); ^13^C NMR (176 MHz, DMSO-*d*_6_): δ 149.0, 138.0, 134.5, 130.2, 120.8, 119.8, 55.0, 20.5; LCMS *m*/*z*: 190.2 [M + H]^+^.

##### ((4-Trifluoromethylphenyl)-1*H*-1,2,3-triazol-4-yl)methanol (**7l**)

Prepared by General Method 1.2 from 4-trifluoromethylaniline and propargyl alcohol and isolated as a white solid (294 mg, 65%).

^1^H NMR (700 MHz, DMSO-*d*_6_): δ 8.84 (s, 1H), 8.17 (d, *J* = 8.4 Hz, 2H), 7.97 (d, *J* = 8.5 Hz, 2H), 5.38 (t, *J* = 5.6 Hz, 1H), 4.63 (d, *J* = 5.3 Hz, 2H); ^13^C NMR (176 MHz, DMSO-*d*_6_): δ 149.6, 139.5, 128.5 (q, *J* = 32.4 Hz), 127.2 (q, *J* = 3.6 Hz), 123.8 (q, *J* = 272.2 Hz), 121.2, 120.3, 54.9; LCMS *m*/*z*: 244.2 [M + H]^+^.

##### ((4-Cyanophenyl)-1*H*-1,2,3-triazol-4-yl)methanol (**7m**)

Prepared by General Method 1.1 from 4-cyanophenylboronic acid and isolated as an off-white solid (53 mg, 13%).

^1^H NMR (600 MHz, DMSO-*d*_6_): δ 8.85 (s, 1H), 8.21–8.12 (m, 2H), 8.12–8.03 (m, 2H), 5.38 (t, *J* = 5.6 Hz, 1H), 4.63 (d, *J* = 5.5 Hz, 2H); ^13^C NMR (151 MHz, DMSO-*d*_6_): δ 149.7, 139.7, 134.4, 121.3, 120.3, 118.2, 110.9, 54.9; LCMS *m*/*z*: 201.2 [M + H]^+^.

##### ((4-Ethoxyphenyl)-1*H*-1,2,3-triazol-4-yl)methanol (**7n**)

Prepared by General Method 1.1 from 4-ethoxyphenylboronic acid and isolated as an off-white solid (224 mg, 51%).

^1^H NMR (600 MHz, DMSO-*d*_6_): δ 8.57 (s, 1H), 7.81–7.75 (m, 2H), 7.13–7.07 (m, 2H), 5.31 (t, *J* = 5.6 Hz, 1H), 4.59 (d, *J* = 5.6 Hz, 2H), 4.09 (q, *J* = 7.0 Hz, 2H), 1.35 (t, *J* = 7.0 Hz, 3H); ^13^C NMR (151 MHz, DMSO-*d*_6_): δ 158.4, 148.9, 130.1, 121.6, 121.0, 115.3, 63.6, 55.0, 14.6; LCMS *m*/*z*: 220.2 [M + H]^+^.

##### (1-(2,3-Dihydrobenzo[*b*][1,4]dioxin-6-yl)-1*H*-1,2,3-triazol-4-yl)methanol (**7o**)

Prepared by General Method 1.1 from 2,3-dihydro-1,4-benzodioxin-6-ylboronic acid and isolated as an off-white solid (124 mg, 27%).

^1^H NMR (700 MHz, DMSO-*d*_6_): δ 8.56 (s, 1H), 7.41 (d, *J* = 2.6 Hz, 1H), 7.35 (dd, *J* = 8.7, 2.6 Hz, 1H), 7.04 (d, *J* = 8.7 Hz, 1H), 5.29 (t, *J* = 5.6 Hz, 1H), 4.58 (dd, *J* = 5.6, 0.5 Hz, 2H), 4.37–4.27 (m, 4H); ^13^C NMR (176 MHz, DMSO-*d*_6_): δ 148.8, 143.8, 143.5, 130.5, 121.0, 117.9, 113.0, 109.1, 64.2, 64.1, 55.0; LCMS *m*/*z*: 234.2 [M + H]^+^.

##### (1-(Benzo[*d*][1,3]dioxol-5-yl)-1*H*-1,2,3-triazol-4-yl)methanol (**7p**)

Prepared by General Method 1.1 from 1,3-benzodioxol-5-ylboronic acid and isolated as an off-white solid (174 mg, 40%).

^1^H NMR (700 MHz, DMSO-*d*_6_): δ 8.56 (s, 1H), 7.49 (d, *J* = 2.2 Hz, 1H), 7.36 (dd, *J* = 8.4, 2.2 Hz, 1H), 7.09 (d, *J* = 8.4 Hz, 1H), 6.14 (s, 2H), 5.30 (t, *J* = 5.6 Hz, 1H), 4.59 (dd, *J* = 5.6, 0.6 Hz, 2H); ^13^C NMR (176 MHz, DMSO-*d*_6_): δ 148.9, 148.2, 147.3, 131.2, 121.1, 113.6, 108.6, 102.1, 101.9, 55.0; LCMS *m*/*z*: 220.1 [M + H]^+^.

##### ((2,3-Dichlorophenyl)-1*H*-1,2,3-triazol-4-yl)methanol (**7q**)

Prepared by General Method 1.1 from 2,3-dichlorophenylboronic acid and isolated as an off-white solid (97 mg, 20%).

^1^H NMR (700 MHz, DMSO-*d*_6_): δ 8.45–8.44 (m, 1H), 7.91 (dd, *J* = 8.1, 1.5 Hz, 1H), 7.66 (dd, *J* = 8.0, 1.5 Hz, 1H), 7.61 (t, *J* = 8.0 Hz, 1H), 5.36 (t, *J* = 5.7 Hz, 1H), 4.63 (dd, *J* = 5.6, 0.6 Hz, 2H); ^13^C NMR (176 MHz, DMSO-*d*_6_): δ 148.2, 136.4, 132.9, 131.9, 129.0, 127.7, 127.3, 125.0 54.8; LCMS *m*/*z*: 244.1 [M + H]^+^.

##### ((2,4-Dichlorophenyl)-1*H*-1,2,3-triazol-4-yl)methanol (**7r**)

Prepared by General Method 1.1 from 2,4-dichlorophenylboronic acid and isolated as an off-white solid (82 mg, 17%).

^1^H NMR (700 MHz, DMSO-*d*_6_): δ 8.41 (s, 1H), 7.99 (q, *J* = 2.3 Hz, 1H), 7.69 (ddd, *J* = 10.8, 8.5, 1.2 Hz, 2H), 5.44–5.11 (m, 1H), 4.62 (dd, *J* = 5.6, 0.6 Hz, 2H); ^13^C NMR (176 MHz, DMSO-*d*_6_): δ 148.2, 135.2, 133.8, 130.1, 129.7, 129.6, 128.6, 124.9, 54.8; LCMS *m*/*z*: 244.1 [M + H]^+^.

##### ((2,5-Dichlorophenyl)-1*H*-1,2,3-triazol-4-yl)methanol (**7s**)

Prepared by General Method 1.1 from 2,5-dichlorophenylboronic acid and isolated as an off-white solid (90 mg, 18%).

^1^H NMR (700 MHz, DMSO-*d*_6_): δ 8.43 (d, *J* = 0.5 Hz, 1H), 7.87 (d, *J* = 2.5 Hz, 1H), 7.81 (d, *J* = 8.7 Hz, 1H), 7.75–7.70 (m, 1H), 5.36 (t, *J* = 5.6 Hz, 1H), 4.63 (dd, *J* = 5.6, 0.6 Hz, 2H); ^13^C NMR (176 MHz, DMSO-*d*_6_): δ 148.2, 135.6, 132.4, 131.9, 131.3, 128.1, 127.5, 124.9, 54.8; LCMS *m*/*z*: 244.1 [M + H]^+^.

##### ((3,5-Dichlorophenyl)-1*H*-1,2,3-triazol-4-yl)methanol (**7t**)

Prepared by General Method 1.1 from 3,5-dichlorophenylboronic acid and isolated as an off-white solid (109 mg, 22%).

^1^H NMR (600 MHz, DMSO-*d*_6_): δ 8.85 (s, 1H), 8.09 (d, *J* = 1.8 Hz, 2H), 7.76 (t, *J* = 1.8 Hz, 1H), 5.41 (t, *J* = 5.5 Hz, 1H), 4.61 (d, *J* = 5.4 Hz, 2H); ^13^C NMR (151 MHz, DMSO-*d*_6_): δ 149.6, 138.4, 135.3, 133.7, 132.3, 127.8, 121.4, 118.5, 54.9; LCMS *m*/*z*: 244.1 [M + H]^+^.

##### ((3,4-Dimethylphenyl)-1*H*-1,2,3-triazol-4-yl)methanol (**7u**)

Prepared by General Method 1.1 from 3,4-dimethylphenylboronic acid and isolated as an off-white solid (131 mg, 32%).

^1^H NMR (400 MHz, DMSO-*d*_6_): δ 8.58 (s, 1H), 7.70 (d, *J* = 2.1 Hz, 1H), 7.59 (dd, *J* = 8.1, 2.4 Hz, 1H), 7.33 (d, *J* = 8.2 Hz, 1H), 5.28 (t, *J* = 5.6 Hz, 1H), 4.69–4.51 (m, 2H), 2.32 (s, 3H), 2.28 (s, 3H); ^13^C NMR (151 MHz, DMSO-*d*_6_): δ 149.0, 138.1, 136.8, 134.7, 130.6, 120.8, 120.8, 117.1, 55.0, 19.5, 19.0; LCMS *m*/*z*: 204.2 [M + H]^+^.

##### ((3,4-Bis(trifluoromethyl)phenyl)-1*H*-1,2,3-triazol-4-yl)methanol (**7v**)

Prepared by General Method 1.3 from 3,4-bis(trifluoromethyl)aniline and propargyl alcohol and isolated as a white solid (64 mg, 38%).

^1^H NMR (400 MHz, CDCl_3_): δ 8.29 (s, 1H), 8.13–8.03 (m, 3H), 4.94 (s, 2H), 2.34 (s, 1H); LCMS *m*/*z*: 312.0 [M + H]^+^.

##### ((4-Chloro-3-(trifluoromethyl)phenyl)-1*H*-1,2,3-triazol-4-yl)methanol (**7w**)

Prepared by General Method 1.4 from 4-chloro-3-trifluoromethylaniline and propargyl alcohol and isolated as a white solid (1.18 g, 83%).

^1^H NMR (700 MHz, DMSO-*d*_6_): δ 8.89 (s, 1H), 8.35 (d, *J* = 2.5 Hz, 1H), 8.26 (dd, *J* = 8.7, 2.6 Hz, 1H), 7.95 (d, *J* = 8.7 Hz, 1H), 5.37 (t, *J* = 5.6 Hz, 1H), 4.62 (d, *J* = 5.5 Hz, 2H); ^13^C NMR (176 MHz, DMSO-*d*_6_): δ 149.6, 135.7, 133.3, 130.1, 127.9 (q, *J* = 31.6 Hz), 125.1, 122.3 (q, *J* = 273.4 Hz), 121.4, 119.1 (q, *J* = 5.4 Hz), 54.9; LCMS *m*/*z*: 278.1 [M + H]^+^.

##### ((3-Chloro-4-(trifluoromethyl)phenyl)-1*H*-1,2,3-triazol-4-yl)methanol (**7x**)

Prepared by General Method 1.4 from 3-chloro-4-(trifluoromethyl)aniline and propargyl alcohol and isolated as a white solid (248 mg, 65%).

^1^H NMR (400 MHz, DMSO-*d*_6_): δ 8.92 (s, 1H), 8.38 (d, *J* = 1.9 Hz, 1H), 8.16 (dd, *J* = 8.6, 1.4 Hz, 1H), 8.08 (d, *J* = 8.7 Hz, 1H), 5.41 (t, *J* = 5.6 Hz, 1H), 4.63 (d, *J* = 5.4 Hz, 2H); ^13^C NMR (151 MHz, DMSO-*d*_6_): δ 149.8, 140.2, 132.4, 129.7 (q, *J* = 5.2 Hz), 125.9 (q, *J* = 31.4 Hz), 122.6 (q, *J* = 272.7 Hz), 122.2, 121.5, 118.4, 54.9; LCMS *m*/*z*: 278.1[M + H]^+^.

##### ((2,4-Dichloro-3-(trifluoromethyl)phenyl)-1*H*-1,2,3-triazol-4-yl)methanol (**7y**)

Prepared by General Method 1.3 from 2,4-dichloro-3-(trifluoromethyl)aniline **11a** and propargyl alcohol and isolated as a white solid (74 mg, 44%).

^1^H NMR (700 MHz, DMSO-*d*_6_): δ 8.43 (t, *J* = 0.6 Hz, 1H), 7.97 (q, *J* = 9.0 Hz, 2H), 5.38 (t, *J* = 5.6 Hz, 1H), 4.63 (dt, *J* = 6.3, 3.2 Hz, 2H); ^13^C NMR (176 MHz, DMSO-*d*_6_): δ 148.3, 136.1, 134.8, 132.8, 132.1, 130.6, 125.6 (q, *J* = 30.2 Hz), 125.4, 122.1 (q, *J* = 276.6 Hz), 54.8; LCMS *m*/*z*: 312.0 [M + H]^+^.

##### 1-(1-(2,4-Dichloro-3-(trifluoromethyl)phenyl)-1*H*-1,2,3-triazol-4-yl)ethan-1-ol (**8a**)

Prepared by General Method 1.3 from 2,4-dichloro-3-trifluoromethylaniline **11a** and 3-butyn-2-ol and isolated as a white solid (40 mg, 19%).

^1^H NMR (400 MHz, CDCl_3_): δ 7.87 (d, *J* = 0.6 Hz, 1H), 7.67–7.61 (m, 2H), 5.20 (q, *J* = 6.6 Hz, 1H), 2.51 (s, 1H), 1.68 (d, *J* = 6.6 Hz, 3H); LCMS *m*/*z*: 326.1 [M + H]^+^.

##### 2-(1-(2,4-Dichloro-3-(trifluoromethyl)phenyl)-1*H*-1,2,3-triazol-4-yl)ethan-1-ol (**8b**)

Prepared by General Method 1.4 from 2,4-dichloro-3-(trifluoromethyl)aniline **11a** and 3-butyn-1-ol and isolated as an off-white solid (55 mg, 19%).

^1^H NMR (600 MHz, DMSO-*d*_6_): δ 8.33 (d, *J* = 4.7 Hz, 1H), 8.00–7.90 (m, 2H), 4.76 (s, 1H), 3.71 (t, *J* = 6.3 Hz, 2H), 2.89 (dt, *J* = 6.9 Hz, *J* = 3.4 Hz, 2H); ^13^C NMR (151 MHz, DMSO-*d*_6_): δ 144.8, 136.1, 134.7, 132.7, 132.1, 130.8–129.7 (m), 125.7 (q, *J* = 29.8 Hz), 125.1, 122.1 (q, *J* = 276.9 Hz), 60.1, 29.0; LCMS *m*/*z*: 326.1 [M + H]^+^.

##### 3-(1-(2,4-Dichloro-3-(trifluoromethyl)phenyl)-1*H*-1,2,3-triazol-4-yl)propan-1-ol (**8c**)

Prepared by General Method 1.3 from 2,4-dichloro-3-(trifluoromethyl)aniline **11a** and 4-pentyn-1-ol and isolated as a clear, colorless oil (94 mg, 58%).

^1^H NMR (600 MHz, CDCl_3_): δ 7.71 (s, 1H), 7.63 (q, *J* = 8.7 Hz, 1H), 7.65 (d, *J* = 8.7 Hz, 1H), 7.62 (d, *J* = 8.7 Hz, 1H), 3.77 (t, *J* = 6.0 Hz, 1H), 2.95 (t, *J* = 7.4 Hz, 1H), 2.05–1.99 (m, 3H); ^13^C NMR (151 MHz, CDCl_3_): δ 147.9, 136.3, 136.0, 131.6, 131.1, 130.8, 128.0 (q, *J* = 31 Hz), 123.5, 122.1 (q, *J* = 278 Hz), 62.0, 31.9, 22.1; LCMS *m*/*z*: 340.2 [M + H]^+^.

##### 1-(1-(2,4-Dichloro-3-(trifluoromethyl)phenyl)-1*H*-1,2,3-triazol-4-yl)propan-2-ol (**8d**)

Prepared by General Method 1.4 from 2,4-dichloro-3-(trifluoromethyl)aniline **11a** and 4-pentyn-2-ol and isolated as an off-white, waxy solid (71 mg, 37%).

^1^H NMR (600 MHz, CDCl_3_): δ 8.01 (s, 1H), 7.67 (d, *J* = 8.8 Hz, 1H), 7.63 (d, *J* = 8.4 Hz, 1H), 4.42 (br s, 1H), 3.14–2.32 (m, 3H), 1.51–1.36 (m, 3H); ^13^C NMR (176 MHz, CDCl_3_): δ 136.7, 131.7 (q, *J* = 6.3 Hz), 131.5, 131.2, 131.0, 130.9, 130.8, 128.0 (q, *J* = 31.1 Hz), 122.2 (q, *J* = 277.2 Hz), 67.7, 34.1, 29.8; LCMS *m*/*z*: 340.1 [M + H]^+^.

##### 3-((1-(2,4-Dichloro-3-(trifluoromethyl)phenyl)-1*H*-1,2,3-triazol-4-yl)methyl)oxetan-3-ol (**8e**)

Prepared by General Method 1.4 from 2,4-dichloro-3-(trifluoromethyl)aniline **11a** and 3-prop-2-ynyloxetan-3-ol and isolated as a white solid (75 mg, 22%).

^1^H NMR (600 MHz, DMSO-*d*_6_): δ 8.33 (s, 1H), 7.98–7.93 (m, 2H), 4.76 (t, *J* = 5.3 Hz, 1H), 3.71 (td, *J* = 6.9, 5.3 Hz, 2H), 2.89 (dt, *J* = 6.9, 3.5 Hz, 2H), 1.25–1.22 (m, 2H); LCMS *m*/*z*: 368.1 [M + H]^+^.

##### 1-(2,4-Dichloro-3-(trifluoromethyl)phenyl)-1*H*-1,2,3-triazole-4-carboxylic Acid (**8f**)

Methyl 1-(2,4-dichloro-3-(trifluoromethyl)phenyl)-1*H*-1,2,3-triazole-4-carboxylate (**14**) was prepared by General Method 1.3 from 2,4-dichloro-3-(trifluoromethyl)aniline **11a** and methyl propiolate and isolated as a white solid (1.05 g, 71%).

^1^H NMR (700 MHz, DMSO-*d*_6_): δ 9.28 (s, 1H), 8.11 (d, *J* = 8.7 Hz, 1H), 8.03 (d, *J* = 8.7 Hz, 1H), 3.90 (s, 3H); LCMS: MS *m*/*z*: 340.0 [M + H]^+^.

Aqueous NaOH (2.65 mL, 1 M, 2.65 mmol, 2.0 equiv) was added to a solution of **14** (450 mg, 1.32 mmol, 1.0 equiv) in MeOH (30 mL). The reaction was stirred at RT for 1 h, acidified to pH 3 with aqueous HCl (2 M), extracted with EtOAc (2 × 100 mL), and washed with brine (50 mL). The organic phase was dried, filtered, and concentrated under reduced pressure to give **8f**. The product was isolated as an off-white solid (410 mg, 95%).

^1^H NMR (700 MHz, DMSO-*d*_6_): δ 13.45 (s, 1H), 9.16 (s, 1H), 8.10 (d, *J* = 8.6 Hz, 1H), 8.05–7.98 (m, 1H); ^13^C NMR (176 MHz, DMSO-*d*_6_): δ 161.3, 140.1, 135.5, 135.3, 133.1, 132.2, 131.6, 130.9, 125.7 (q, *J* = 30.3 Hz), 122.1 (q, *J* = 276.7 Hz); LCMS *m*/*z*: 325.9 [M + H]^+^.

##### 1-(2,4-Dichloro-3-(trifluoromethyl)phenyl)-1*H*-1,2,3-triazole-4-carboxamide (**8g**)

NH_3_ (1.0 mL, 2 M in MeOH, 2.0 mmol, 13.3 equiv) was added to **14** (50 mg, 0.15 mmol, 1.0 equiv) and heated to 70 °C for 3 h. The reaction was concentrated under reduced pressure, triturated with Et_2_O (ca. 2 mL), and then dried in vacuo to give **8g**. The product was isolated as a white solid (22 mg, 46%).

^1^H NMR (700 MHz, DMSO-*d*_6_): δ 8.98 (s, 1H), 8.09 (d, *J* = 8.7 Hz, 1H), 8.07 (s, 1H), 8.01 (d, *J* = 8.7 Hz, 1H), 7.65 (s, 1H); ^13^C NMR (176 MHz, DMSO-*d*_6_): δ 160.9, 143.1, 135.5, 135.4, 133.0, 132.2, 130.8, 129.4, 125.7 (q, *J* = 30.2 Hz), 122.1 (q, *J* = 276.6 Hz); LCMS *m*/*z*: 325.0 [M + H]^+^.

##### 1-(2,4-Dichloro-3-(trifluoromethyl)phenyl)-*N*-methyl-1*H*-1,2,3-triazole-4-carboxamide (**8h**)

MeNH_2_ (1.0 mL, 2 M in MeOH, 2.0 mmol, 13.3 equiv) was added to **14** (50 mg, 0.15 mmol, 1.0 equiv) and heated to 70 °C for 3 h. The reaction was concentrated under reduced pressure, triturated with Et_2_O (ca. 2 mL), and then dried in vacuo to give **8h**. The product was isolated as a white solid (32 mg, 64%).

^1^H NMR (700 MHz, DMSO-*d*_6_): δ 9.00 (s, 1H), 8.68 (q, *J* = 4.4 Hz, 1H), 8.09 (apparent dd, *J* = 8.6, 0.5 Hz, 1H), 8.01 (apparent dd, *J* = 8.7, 0.6 Hz, 1H), 2.81 (d, *J* = 4.7 Hz, 3H); ^13^C NMR (176 MHz, DMSO-*d*_6_): δ 159.6, 143.1, 135.5, 135.4, 133.0, 132.2, 130.9, 129.0, 125.7 (q, *J* = 30.2 Hz), 122.1 (q, *J* = 276.7 Hz), 25.7; LCMS *m*/*z*: 339.0 [M + H]^+^.

##### (1-(2,4-Dichloro-3-(trifluoromethyl)phenyl)-1*H*-1,2,3-triazol-4-yl)methanamine Hydrochloride (**8i**)

Steps 1 and 2: **15** was prepared by General Method 1.2 from 2,4-dichloro-3-(trifluoromethyl)aniline **11a** and *tert*-butyl prop-2-yn-1-ylcarbamate and isolated as a white solid (64 mg, 8%).

^1^H NMR (500 MHz, DMSO-*d*_6_): δ 8.34 (s, 1H), 8.01–7.90 (m, 2H), 7.42 (t, *J* = 5.5 Hz, 1H), 4.29 (d, *J* = 5.5 Hz, 2H), 1.39 (s, 9H); LCMS *m*/*z*: = 433.1 [M + Na]^+^.

Step 3: HCl (0.47 mL, 4 M in dioxane, 1.88 mmol, 11.8 equiv) was added to **15** (64 mg, 0.16 mmol, 1.0 equiv) in Et_2_O (2.0 mL) and heated to 50 °C for 16 h. The reaction was concentrated under reduced pressure to give **8i**·HCl. The product was isolated as an off-white solid (54 mg, quant).

^1^H NMR (600 MHz, DMSO-*d*_6_): δ 8.65 (s, 1H), 8.51 (s, 3H), 8.00 (d, *J* = 8.8 Hz, 1H), 7.97 (d, *J* = 8.7 Hz, 1H), 4.26 (s, 2H); ^13^C NMR (151 MHz, DMSO-*d*_6_): δ 140.6, 135.7, 135.2, 132.8, 132.3, 130.5, 127.3, 125.8 (q, *J* = 30.2 Hz), 122.1 (q, *J* = 276.7 Hz), 33.7 (s, *J* = 18.5 Hz); LCMS *m*/*z*: 310.9 [M + H]^+^.

##### 1-(2,4-Dichloro-3-(trifluoromethyl)phenyl)-4-(methoxymethyl)-1*H*-1,2,3-triazole (**8j**)

Prepared by General Method 1.3 from 2,4-dichloro-3-(trifluoromethyl)aniline **11a** and 3-methoxyprop-1-yne and isolated as a white solid (79 mg, 51%).

^1^H NMR (600 MHz, DMSO-*d*_6_): δ 8.59 (s, 1H), 8.03 (d, *J* = 8.7 Hz, 1H), 7.98 (d, *J* = 8.7 Hz, 1H), 4.57 (s, 2H), 3.33 (s, 3H); ^13^C NMR (151 MHz, DMSO-*d*_6_): δ 144.1, 136.0, 135.0, 133.0, 132.2, 130.7, 126.7, 125.7 (q, *J* = 30.2 Hz), 122.1 (q, *J* = 276.7 Hz), 64.7, 57.5; LCMS *m*/*z*: 326.0 [M + H]^+^.

##### 1-(2,4-Dichloro-3-(trifluoromethyl)phenyl)-4-methyl-1*H*-1,2,3-triazole (**8k**)

*Caution* Step 1: Sodium nitrite (180 mg, 2.61 mmol, 1.2 equiv) was added portionwise to a solution of 2,4-dichloro-3-(trifluoromethyl)aniline **11a** (500 mg, 2.17 mmol, 1.0 equiv) in TFA (5 mL, 4.35 mmol) at 0 °C over 30 min. The reaction was then allowed to warm to RT for 1.5 h, H_2_O (0.1 mL) was added, and the mixture was then recooled to 0 °C. Sodium azide (155 mg, 2.39 mmol, 1.1 equiv) was added portionwise over 30 min, and the reaction mixture was then allowed to warm to RT over 1 h. The mixture was basified to pH 8–9 by dropwise addition of saturated aqueous NaCO_3_H, then diluted with EtOAc (ca. 50 mL). The organic phase was separated, diluted with *t*-BuOH (5.0 mL), and then cautiously concentrated under reduced pressure at 25 °C to provide a solution of crude azide **12a** in *t*-BuOH, which was used without further purification.

Step 2: MeOH (5 mL), H_2_O (5 mL), sodium l-ascorbate (172 mg, 0.9 mmol, 0.4 equiv), trimethyl(propargyl)silane (244 mg, 2.2 mmol, 1.0 equiv), and copper(II) sulfate pentahydrate (109 mg, 0.43 mmol, 0.2 equiv) were added to the solution of crude azide **12a** from step one, and the reaction mixture was stirred at 50 °C for 16 h. The reaction was cooled to RT, diluted with H_2_O (ca. 10 mL), extracted with EtOAc (ca. 50 mL), and the organic phase was washed with H_2_O (ca. 10 mL) and brine (ca. 10 mL). The organic phase was dried, filtered, and concentrated under reduced pressure to give **16**, which was used immediately without further purification.

Step 3: K_2_CO_3_ (3.05 g, 21.7 mmol, 10.0 equiv) was added to crude **16** from step 2 in MeOH (15 mL) and heated to reflux for 16 h. The mixture was taken to pH 7 by addition of aqueous HCl (1 M), then diluted with EtOAc (ca. 50 mL). The organic phase was dried, filtered, and concentrated under reduced pressure. Due to incomplete desilylation, the crude material was suspended in THF (10 mL) and TBAF hydrate (1.22 g, 4.35 mmol, 2.0 equiv) in H_2_O (0.5 mL) was added. The reaction was stirred at RT for 16 h, diluted with EtOAc (ca. 50 mL), and then the organic phase was dried, filtered, and concentrated under reduced pressure. The crude product was purified by column chromatography, eluting with 0–30% EtOAc in cyclohexane to give **8k** isolated as an off-white solid (12 mg, 2%).

^1^H NMR (700 MHz, CDCl_3_): δ 7.67 (d, *J* = 0.8 Hz, 1H), 7.64 (d, *J* = 8.6 Hz, 1H), 7.61 (d, *J* = 8.7 Hz, 1H), 2.47 (d, *J* = 0.8 Hz, 3H); ^13^C NMR (176 MHz, CDCl_3_): δ 143.7, 136.2 (q, *J* = 1.2 Hz), 136.1, 131.6, 131.0, 130.7, 128.0 (q, *J* = 31.1 Hz), 123.6, 122.2 (q, *J* = 277.2 Hz), 10.9; LCMS *m*/*z*: 296.0 [M + H]^+^.

##### 1-(2,4-Dichloro-3-(trifluoromethyl)phenyl)-1*H*-1,2,3-triazole (**8l**)

*Caution* Step 1: 2,4-Dichloro-3-(trifluoromethyl)aniline **11a** (1.0 g, 4.3 mmol, 1.0 equiv) was dissolved in TFA (20 mL), cooled to 0 °C, a solution of sodium nitrite (1.4 g, 20.6 mmol, 4.7 equiv) in H_2_O (2.8 mL) was added dropwise over 10 min, and stirred for a further 30 min. The ice bath was removed, the reaction was stirred at RT for 1 h, and then recooled to 0 °C, before a solution of sodium azide (1.3g, 20.6 mmol, 4.7 equiv) in H_2_O (5.4 mL, 20.6 mmol) was added dropwise over 30 min. The reaction was allowed to warm to RT over 16 h, before recooling to 0 °C. Aqueous NaOH (52 mL, 5 M) was then cautiously added to take the mixture to pH 8–9 to provide a solution of crude azide **12a**, which was used without further purification.

Step 2: MeOH (10 mL), *t*-BuOH (10 mL), aqueous copper(II) sulfate pentahydrate (5.1 mL, 100 mg/mL, 2.1 mmol, 0.5 equiv), aqueous sodium l-ascorbate (4.1 mL, 1 M, 4.1 mmol, 0.9 equiv), and trimethylsilylacetylene (0.94 mL, 8.7 mmol, 2.0 equiv) were added to the solution of crude azide **12a** from step one at RT. The reaction was stirred for 16 h, diluted with EtOAc (ca. 500 mL), and the organic phase was cautiously concentrated under reduced pressure at 25 °C to give crude **17**.

Step 3: MeOH (15 mL) and K_2_CO_3_ (6.0 g, 43.5 mmol) were added to crude **17**, and the reaction was stirred at RT for 24 h, before H_2_O (ca. 20 mL) was then added until all the solid carbonate dissolved. MeOH was then removed under reduced pressure and the resultant aqueous mixture was diluted with EtOAc (ca. 500 mL). The organic phase was dried, filtered, and concentrated under reduced pressure. The crude product was purified by column chromatography (0–30% EtOAc in cyclohexane) to give **8l** and isolated as a white solid (0.83 g, 67%).

mp 111–112 °C; IR ν_max_ (film): 3164, 3141, 3082, 1248, 1198, 1132, 1026, 790 cm^–1^; ^1^H NMR (700 MHz, DMSO-*d*_6_): δ 8.61 (d, *J* = 1.2 Hz, 1H), 8.03 (d, *J* = 1.1 Hz, 1H), 8.01 (apparent dd, *J* = 8.6, 0.5 Hz, 1H), 7.98 (apparent dd, *J* = 8.6, 0.5 Hz, 1H); ^13^C NMR (176 MHz, DMSO-*d*_6_): δ 136.0, 134.9, 133.7, 132.9, 132.1, 130.6, 127.7, 125.7 (q, *J* = 30.2 Hz), 122.1 (q, *J* = 276.6 Hz); LCMS: MS *m*/*z*: 282.0 [M + H]^+^; HRMS C_9_H_4_Cl_2_F_3_N: calcd 281.9807 [M + H]^+^; found, 281.9807.

Spectroscopic and analytical data for **8l** are presented in the Supporting Information.

##### 1-(2,4-Dichloro-3-(trifluoromethyl)phenyl)-1*H*-1,2,3-triazole-4,5-*d*_2_ (*d*_2_-**8l**)

*Caution* Step 1: Sodium nitrite (360 mg, 5.22 mmol, 1.2 equiv) was added portionwise to a solution of 2,4-dichloro-3-(trifluoromethyl)aniline **11a** (1.0 g, 4.35 mmol, 1.0 equiv) in TFA (10 mL) at 0 °C over 30 min. The reaction mixture was then allowed to warm to RT over 90 min. H_2_O (1.0 mL) was added and then the mixture was recooled to 0 °C. Sodium azide (310 mg, 4.78 mmol, 1.1 equiv) was added portionwise over 30 min, then the reaction was allowed to warm to RT over 1 h. The mixture was basified to pH 8–9 by dropwise addition of saturated aqueous NaHCO_3_ and then extracted with CH_2_Cl_2_ (ca. 250 mL). The organic phase was separated, diluted with *t*-BuOH (10.0 mL), and then cautiously concentrated under reduced pressure at 25 °C to provide a solution of crude azide **12a** in *t*-BuOH, which was used without further purification.

Step 2: Copper(II) sulfate pentahydrate (217 mg, 0.87 mmol, 0.2 equiv), sodium l-ascorbate (345 mg, 1.74 mmol, 0.4 equiv), H_2_O (10 mL), and then trimethylsilylacetylene (0.6 mL, 4.35 mmol, 1.0 equiv) were added to the solution of crude azide **12a** from step 1. The reaction mixture was stirred at RT for 16 h, diluted with H_2_O (20 mL), extracted with EtOAc (ca. 100 mL), and the organic phase was washed with H_2_O (ca. 20 mL) and brine (ca. 20 mL). The organic phase was dried, filtered, and concentrated under reduced pressure. The crude product was purified by column chromatography (0–30% EtOAc in cyclohexane) to give **17** (390 mg, 1 .1 mmol, 25%) as an oily solid.

^1^H NMR (400 MHz, DMSO-*d*_6_): δ 8.62 (s, 1 H), 8.01 (d, *J* = 8.8 Hz, 1 H), 7.96 (d, *J* = 8.8 Hz, 1 H), 0.33 (s, 9H).

Step 3: **17** (100 mg, 0.28 mmol, 1.0 equiv) and K_2_CO_3_ (117 mg, 0.85 mmol, 3.0 equiv) were dried for 2 h under vacuum (0.5 mbar, RT). >99% methanol-*d*_4_ (5.0 mL) was added under an argon atmosphere and stirred at RT for 3 days, before addition of >99% D_2_O (5.0 mL). The vessel was quickly transferred to a rotary evaporator and the organics were removed under reduced pressure, then the vessel was quickly resealed. Alumina-treated anhydrous CH_2_Cl_2_ (ca. 10 mL) was added, stirred for 15 min, and then the organic layer was removed via a syringe and concentrated under reduced pressure.

The crude product was purified by column chromatography (0–30% EtOAc in cyclohexane) to give *d*_2_-**8l** (>90% *d*_2_-isotope) and isolated as a white solid (60 mg, 67%).

^1^H NMR (700 MHz, DMSO-*d*_6_): δ 7.98 (apparent dd, *J* = 8.7, 0.5 Hz, 1H), 7.72 (apparent dd, *J* = 8.7, 0.6 Hz, 1H); ^13^C NMR (176 MHz, DMSO-*d*_6_): δ 153.3, 134.14–133.53 (m), 133.0 (q, *J* = 1.6 Hz), 131.6, 131.3, 128.3, 127.06–126.53 (m), 122.4 (q, *J* = 275.7 Hz), 121.8 (q, *J* = 29.5 Hz); LCMS *m*/*z*: 284.2 [M + H]^+^.

##### 1-(2,3,4-Trichlorophenyl)-1*H*-1,2,3-triazole (**8m**)

Steps 1 and 2: azide **12** (R^2^ = R^3^ = R^4^ = Cl) was prepared by General Method 1.4 from 2,3,4-trichloroaniline (200 mg, 1.0 mmol, 1.0 equiv) and trimethyl(propargyl)silane and was used directly in step 3.

Step 3: Na_2_CO_3_ (5.0 g, 47.2 mmol, 47.2 equiv) was added to azide **12** in MeOH (5.0 mL) and stirred vigorously at RT for 16 h. Aqueous HCl (2 M, ca. 60 mL) was cautiously added; once gas evolution had ceased, the organics were removed under reduced pressure and the mixture was extracted with CH_2_Cl_2_ (ca. 60 mL). The organic phase was dried, filtered, and concentrated under reduced pressure. The crude product was purified by column chromatography (0–30% EtOAc in cyclohexane) to give **8m**, which was isolated as an off-white solid (181 mg, 72%).

^1^H NMR (600 MHz, DMSO-*d*_6_): δ 8.62 (d, *J* = 0.9 Hz, 1H), 8.02 (d, *J* = 1.0 Hz, 1H), 7.93 (d, *J* = 8.7 Hz, 1H), 7.75 (d, *J* = 8.7 Hz, 1H); ^13^C NMR (151 MHz, DMSO-*d*_6_): δ 135.1, 134.6, 133.7, 131.7, 129.8, 129.5, 127.6, 127.4; LCMS: MS 248.0 m/z: [M + H]^+^.

##### 4-(2,4-Dichloro-3-(trifluoromethyl)phenyl)-4*H*-1,2,4-triazole (**9a**)

Chlorotrimethylsilane (1.9 mL, 15.0 mmol, 15.0 equiv) was added dropwise to a solution of 2,4-dichloro-3-(trifluoromethyl)aniline **11a** (230 mg, 1.0 mmol, 1.0 equiv), 1,2-diformylhydrazine (264 mg, 3.0 mmol, 3.0 equiv), and NEt_3_ (1.0 mL, 7.0 mmol, 7.0 equiv) in pyridine (9.0 mL) at 0 °C. The reaction was then heated to 115 °C for 2.5 h and then to 130 °C for 20 h. The reaction was cooled to RT, H_2_O (2.0 mL) was added, and then the mixture was concentrated under reduced pressure. The crude product was purified by reversed-phase chromatography (0–100% MeCN in H_2_O, 0.1% NH_4_OH modifier) to give **9a**, which was isolated as a tan solid (140 mg, 50%).

^1^H NMR (700 MHz, DMSO-*d*_6_): δ 8.86 (s, 1H), 8.00 (d, *J* = 8.7, 1H), 7.96 (d, *J* = 8.7 Hz, 1H); ^13^C NMR (176 MHz, DMSO-*d*_6_): δ 143.3, 134.2, 133.4, 133.1, 132.0, 130.9, 125.5 (q, *J* = 30.2 Hz), 122.1 (q, *J* = 276.6 Hz); LCMS *m*/*z*: 282.1 [M + H]^+^.

##### 1-(2,4-Dichloro-3-(trifluoromethyl)phenyl)-1*H*-tetrazole (**9b**)

*Caution* Trimethyl orthoformate (0.39 mL, 3.26 mmol, 3.0 equiv) and sodium azide (219 mg, 3.37 mmol, 3.1 equiv) were added to a solution of 2,4-dichloro-3-(trifluoromethyl)aniline **11a** (250 mg, 1.09 mmol, 1.0 equiv) in AcOH (2.5 mL). The reaction was heated behind a blast shield to 100 °C for 90 min, then cautiously concentrated (ca. 1 mL solvent remaining) under reduced pressure at 25 °C. H_2_O (30 mL) was added to the resultant residue at RT and the resultant white crystalline precipitated was collected by filtration, washed with H_2_O (ca. 10 mL), and dried in vacuo to give **9b**. The product was isolated as a white crystalline solid (31 mg, 0.11 mmol, 10%).

^1^H NMR (700 MHz, DMSO-*d*_6_): δ 9.90 (s, 1H), 8.15 (apparent dd, *J* = 8.7, 0.5 Hz, 1H), 8.06 (apparent dd, *J* = 8.7, 0.5 Hz, 1H); ^13^C NMR (176 MHz, DMSO-*d*_6_): δ 145.4 (apparent d, *J* = 16.9 Hz), 136.0 (apparent d, *J* = 1.2 Hz), 133.1, 132.7, 132.4, 130.9 (apparent d, *J* = 1.1 Hz), 125.9 (q, *J* = 30.4 Hz), 122.0 (q, *J* = 276.7 Hz); LCMS *m*/*z*: 283.0 [M + H]^+^.

##### 3,5-Dichloro-2-(1*H*-1,2,3-triazol-1-yl)-4-(trifluoromethyl)pyridine (**9c**)^57^

*Caution* Step 1: Hydrazine monohydrate (60 μL, 1.2 mmol, 1.5 equiv) was added to a solution of 2,3,5-trichloro-4-trifluoromethyl pyridine **18** (200 mg, 0.80 mmol, 1.0 equiv) in EtOH (0.5 mL). The reaction was slowly warmed to 85 °C behind a blast shield over 4 h, concentrated under reduced pressure, and the resultant solid was suspended in aqueous HCl (1 M, 10 mL) at RT, then cooled to 0 °C. A solution of sodium nitrite (110 mg, 1 .6 mmol) in H_2_O (0.5 mL) was added over 30 min.

Step 2: The mixture was stirred for a further 30 min before cautious addition of aqueous NaOH (5 M) to adjust the pH to 8–9. H_2_O (2.0 mL), *t*-BuOH (2.0 mL), sodium l-ascorbate (159 mg, 0.8 mmol, 1.0 equiv), copper(II) sulfate pentahydrate (20 mg, 0.08 mmol, 0.1 equiv), and trimethylsilylacetylene (110 μL, 0.8 mmol, 1.0 equiv) were added and the reaction was heated to 40 °C for 16 h. The mixture was cooled to RT, diluted with EtOAc (ca. 50 mL), the organic phase was dried, filtered, and concentrated under reduced pressure. The crude product was purified by reversed-phase chromatography (0–100% MeCN in H_2_O, 0.1% formic acid modifier) to give **9c**, which was isolated as a white solid (5 mg, 2%).

^1^H NMR (700 MHz, CDCl_3_): δ 8.66 (d, *J* = 0.6 Hz, 1H), 8.08 (d, *J* = 1.2 Hz, 1H), 7.90 (d, *J* = 1.2 Hz, 1H); ^13^C NMR (176 MHz, CDCl_3_): δ 149.2, 147.1, 136.7 (q, *J* = 32.1 Hz), 133.8, 132.0, 126.7, 125.1, 121.2 (q, *J* = 278.6 Hz); LCMS: MS m/z: 283.0 [M + H]^+^.

## Supporting Information Available

Overlay of DSPL screening hits **6**; ADME protocols and data; PK studies; Notum OPTS and TCF/LEF screening data concentration–response curves; ABPP optimization; serine hydrolase selectivity of **1**; kinase selectivity panel; safety pharmacology studies; DSC for azide **12a**; X-ray structure determination including data collection and refinement statistics; ligand electron-density maps; and spectroscopic and analytical data for **8l** (PDF)
Identity and quantification of each serine hydrolase identified in SW620 cells by FP-biotin ABPP (XLSX)
Molecular formula strings and Notum IC_50_ data for **6–9** (CSV)

## Accession Codes

Coordinates for X-ray structures of Notum crystallized with **6b** (7B89) and **8l** (7PK3) have been deposited in the Protein Data Bank. Authors will release the atomic coordinates upon article publication.

## Abbreviations

ABPP: activity-based protein profiling
AD: Alzheimer’s disease
ADME: absorption, distribution, metabolism, and elimination
BBB: blood–brain barrier
CDK14: cyclin-dependent kinase 14
CK1: casein kinase 1
CNS: central nervous system
CYP450: cytochrome P450
DMSO: dimethylsulfoxide
DSPL: Diamond-SGC Poised Library
ER: efflux ratio
FBDD: fragment-based drug design
GSK3β: glycogen synthase kinase 3β
HLM: human liver microsomes
HTS: high-throughput screen
*K*_p_: brain to plasma ratio
*K*_p,uu_: free partition coefficient of brain
LE: ligand efficiency
LLE: lipophilic ligand efficiency
MLM: mouse liver microsomes
MPO: multiparameter optimization
MW: molecular weight
OPTS: trisodium 8-octanoyloxypyrene-1,3,6-trisulfonate
PBS: phosphate-buffered saline
PDB: Protein Data Bank
P-gp: P-glycoprotein
PD: pharmacodynamics
PK: pharmacokinetics
RT: room temperature
SAR: structure–activity relationship
SBDD: structure-based drug design
TBAF: tetra-*n*-butylammonium fluoride
TCF/LEF: T-cell factor/lymphoid enhancing factor
TDI: time-dependent inactivation
THF: tetrahydrofuran
TMS: trimethylsilyl
TPSA: topological polar surface area
