# Supplementary material for: Design of a Potent, Selective, and Brain-Penetrant Inhibitor of Wnt-Deactivating Enzyme Notum by Optimization of a Crystallographic Fragment Hit
Source: J Med Chem. 2022 May 10;65(10):7212–30. doi: 10.1021/acs.jmedchem.2c00162 (PMC9150124; doi:10.1021/acs.jmedchem.2c00162)
Supplement: Supplementary file 1 — jm2c00162_si_001.pdf [file jm2c00162_si_001.pdf]

SUPPORTING INFORMATION

Design of a potent, selective and brain penetrant inhibitor of Wnt-deactivating enzyme Notum by optimization of a crystallographic fragment hit

Nicky J. Willis,[1] William Mahy,[1] James Sipthorp,[1,2] Yuguang Zhao,[3] Hannah L. Woodward,[1] Benjamin N. Atkinson,[1] Elliott D. Bayle,[1,2] Fredrik Svensson,[1,2] Sarah Frew,[1] Fiona Jeganathan,[1] Amy Monaghan,[1] Stefano Benvegnu,[1] Sarah Jolly,[1] Luca Vecchia,[3] Reinis R. Ruza,[3] Svend Kjær,[2] Steven Howell,[2] Ambrosius P. Snidjers,[2] Magda Bictash,[1] Patricia C. Salinas,[4] Jean-Paul Vincent,[2] E. Yvonne Jones,[3] Paul Whiting,[1] Paul V. Fish.[1,2,*]

[1] Alzheimer's Research UK UCL Drug Discovery Institute, University College London, Cruciform Building, Gower Street, London, WC1E 6BT, U.K.

[2] The Francis Crick Institute, 1 Midland Road, Kings Cross, London NW1 1AT, U.K.

[3] Division of Structural Biology, Wellcome Centre for Human Genetics, University of Oxford, The Henry Wellcome Building for Genomic Medicine, Roosevelt Drive, Oxford, OX3 7BN, U.K.

[4] Department of Cell and Developmental Biology, Laboratory for Molecular and Cellular Biology, University College London, London, WC1E 6BT, U.K.

* Phone: +44 (0)20 7679 6971; E-mail: p.fish@ucl.ac.uk.

Table of contents:

Page S3	Overlay of fragment screening hits **6a** and **6b** (**Figure S1**)
Page S4	ADME protocols and data (**Tables S1-S9**)
Page S8	Pharmacokinetic studies (**Figures S2-S7 and Tables S10-S19**)
Page S18	Notum OPTS and TCF/LEF screening data concentration-response curves for **8l** (**Figures S8-S10**)
Page S20	Activity-based protein profiling optimization (**Figure S11**)
Page S21	Serine hydrolase selectivity of **1** by ABPP-TMT (**Figure S12)**
Page S22	Kinase selectivity panel (**Figure S13 and Table S20**)
Page S31	Safety pharmacology studies (**Figure S14 and Table S21**)
Page S33	DSC for azide **12a** (**Figure S15 and Table S22**)
Page S34	X-ray structure determination for **6b** and **8l**: data collection and refinement statistics; ligand electron-density maps (**Figure S16 and Table S23**)
Page S36	Spectroscopic and analytical data for 1-(2,4-dichloro-3-(trifluoromethyl)phenyl)-1*H*-1,2,3-triazole (**8l**) (**Figures S17-S22**)
Page S39	SI references

Overlay of fragment screening hits 6a and 6b.

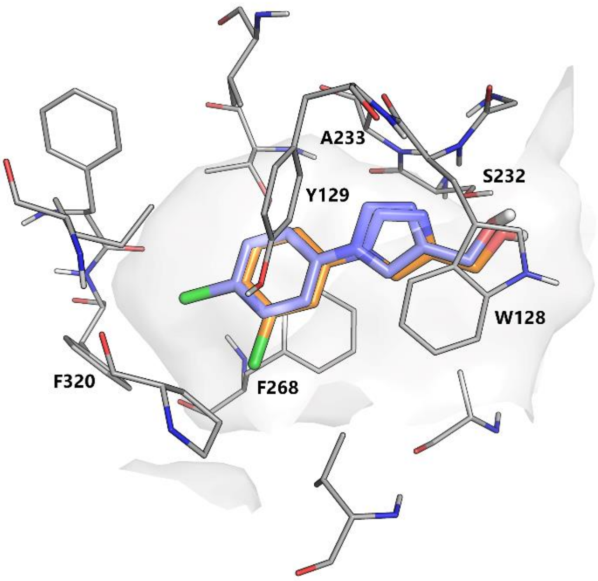

Figure S1. Overlay of **6a** (purple) and **6b** (orange) (using active site of PDB: 6ZUV). Select binding site residues shown. Water molecules have been removed for clarity. The surface of the Notum binding pocket outlined (gray).

ADME protocols and data

ADME studies reported in this work were independently performed by Cyprotex (Macclesfield, U.K.), GVK Biosciences (Hyderabad, India) or WuXi AppTec (Shanghai, China).

A. Distribution coefficient (LogD$_{7.4}$):

Table S1: Representative assay protocol

Method	Octanol/buffer partition coefficient. 500 µL of organic phase (1-octanol) was added to each well of a 2 mL deep well plate, followed by 500 µL of buffer and 15 µL of test substance was added. The plate was vortexed for 1 hr on a plate shaker at 1200 rpm. After incubation, the samples were allowed to equilibrate for 20 min and then centrifuged at 4000 rpm for 30 min for complete phase separation and analysed by LC-UV.
Buffer	10 mM phosphate buffer
pH	7.4
Test compound concentration	0.15 mM
Incubation time	1 hr
No.of replicates	Three
End Point	Fractional distribution of test compound between octanol and buffer
Analysis	LC-UV
QC Compounds	Ketoconazole, Metoprolol and Propranolol

Table S2: Results

**Compound**	**LogD @ pH 7.40**	
	**Mean**	**SD**
**6b**	2.41	-
**8l**	1.31	0.05
Ketoconazole	2.96	0.15
Metoprolol	-0.27	0.02
Propranolol	1.00	0.01

B. Aqueous solubility at pH 7.4:

Representative assay protocols have been described in detail.[1,2]

Table S3: Results

**Compound**	**Thermodynamic Solubility @ RT**	
	**Mean** (mg/mL) (n = 2)	**SD**
**6b**	0.104	0.00215
**7y**	0.213	0.012
**8l**	0.068	0.000395
**9c**	0.399	0.005
d_2-**8l**	0.044	0.005

C. Stability in liver microsomes:

Representative assay protocols have been described in detail.[1,2]

Table S4: Results

Compound	MLM Stability		
	$t_{1/2}$ (min) (n)	Cl_i (μL/min/mg protein)	
		Mean	SE
**6b**	15.8 (5)	87.9	7.37
**7y**	105.14 (2)	6.65	-
**8l**	120.00 (2)	3.19	-
**9c**	120.00 (2)	4.94	-
d$_2$-**8l**	128.81 (2)	5.40	-
**Compound**	**HLM Stability**		
	$t_{1/2}$ (min) (n)	Cl_i (μL/min/mg protein)	
		Mean	SE
**6b**	115 (2)	12.0	1.15
**7y**	552.80 (2)	1.65	-
**8l**	120.00 (2)	1.46	-
**9c**	ND	-	-
d$_2$-**8l**	ND	-	-

ND, not determined.

D. Permeability using MDR1-MDCKII cell monolayer:

Representative assay protocols have been described in detail.[1,2]

Table S5: Results

Compound	MDR1-MDCKII Mean (n=2)				
	**Apical to Basal** P_{app} (10^{-6} cm/sec)	**Basal to Apical** P_{app} (10^{-6} cm/sec)	**Efflux Ratio**	**A to B % Recovery**	**B to A % Recovery**
**6b**	56.6	58.3	1.03	80.7	87.2
**7y**	45.91	27.81	0.61 [a]	94.14	97.50
**8l**	31.41	39.16	1.25	85.12	90.51
**9c**	1.74	4.88	2.81	5.93	65.25
d$_2$-**8l**	47.82	35.37	0.74	102.50	111.06

[a] Fold shift in efflux ration when performed with Elacridar was <1.5, and so unlikely to be a substrate for P-gp.

E. Inhibition of CYP450 enzymes:

Table S6: Results

CYP/Substrate	Compound 8l IC_{50} (μM)		Ratio[a] (-)NADPH/(+)NADPH
CYP1A2/Phenacetin	(-)NADPH	0.964	1.00
	(+)NADPH	0.963	
CYP2B6/Bupropion	(-)NADPH	3.15	0.605
	(+)NADPH	5.21	
CYP2C8/Amodiaquine	(-)NADPH	>50	
	(+)NADPH	>50	
CYP2C9/Diclofenac	(-)NADPH	19.6	1.27
	(+)NADPH	15.4	
CYP2C19/S-Mephenytoin	(-)NADPH	7.27	0.901
	(+)NADPH	8.07	
CYP2D6/Dextromethorphan	(-)NADPH	>50	
	(+)NADPH	>50	
CYP3A4/Midazolam	(-)NADPH	>50	
	(+)NADPH	>50	
CYP3A4/Testosterone	(-)NADPH	>50	
	(+)NADPH	>50	

[a] IC_{50} shift ratio (-)NADPH/(+)NADPH pre-incubation ≥ 1.5 is considered to be a TDI. This assay was performed with standard inhibitors as positive controls, which all passed QC criteria.

F. Permeability using Caco-2 cell monolayer:

Assay protocol: Caco-2 cells were seeded onto polyethylene membranes (PET) in 96-well Corning Insert plates at 1 x 10^5 cells/cm^2, and refreshed medium every 4-5 days until to the 21st to 28th day for confluent cell monolayer formation. The transport buffer in the study was HBSS with 10.0 mM HEPES at pH 7.40. Test compound was tested at 2.00 μM bi-directionally in duplicate. Digoxin was tested at 10 μM bi-directionally in duplicate, while nadolol and metoprolol were tested at 2.00 μM in A to B direction in duplicate. Final DMSO concentration was adjusted to less than 1%. The plate was incubated for 2 hours in CO_2 incubator at 37±1°C, with 5% CO_2 at saturated humidity without shaking. All samples, after acetonitrile containing internal standard had been added, were centrifuged at 3200 xg for 10 min. For nadolol, 200 μL supernatant solution was diluted with 600 μL ultra-pure water for LC-MS/MS analysis. For metoprolol, 50 μL supernatant solution was diluted with 400 μL ultra-pure water for LC-MS/MS analysis. For digoxin and **8l**, 200 μL supernatant solution was diluted with 200 μL ultra-pure water for LC-MS/MS analysis. Concentrations of test and control compounds in starting solution, donor solution, and receiver solution were quantified by LC-MS/MS methodologies, using peak area ratio of analyte/internal standard. After transport assay was complete, lucifer yellow rejection assay was applied to determine the Caco-2 cell monolayer integrity.

Table S7: Results

Compound	Caco-2 Mean (n=2)				
	**Apical to Basal** P_{app} (10^{-6} cm/sec)	**Basal to Apical** P_{app} (10^{-6} cm/sec)	**Efflux Ratio**	**A to B % Recovery**	**B to A % Recovery**
**8l**	49.4	30.3	0.614	89.1	102.5

G. Additional metabolic stability in liver microsomes:

Representative assay protocols have been described in detail.[1,2]

Table S8: Results

Compound	MLM Stability		
	$t_{1/2}$ (min)	Cl_i (μL/min/mg protein)	
		Mean	SE
**8l**	118.3	11.7	-
**Compound**	**RLM Stability**		
	$t_{1/2}$ (min)	Cl_i (μL/min/mg protein)	
		Mean	SE
**8l**	>145	<9.6	-
**Compound**	**DLM Stability**		
	$t_{1/2}$ (min)	Cl_i (μL/min/mg protein)	
		Mean	SE
**8l**	128.4	10.8	-
**Compound**	**HLM Stability**		
	$t_{1/2}$ (min)	Cl_i (μL/min/mg protein)	
		Mean	SE
**8l**	>145	<9.6	-

H. Metabolic stability in hepatocytes:

Table S9: Results

Compound	Metabolic Stability in Mouse Hepatocytes		
	$t_{1/2}$ (min)	Cl_i (μL/min/mg protein)	
		Mean	SE
**7y**	35.86	48.35	-
**8l**	74.1	19.4	-
**Compound**	**Metabolic Stability in Rat Hepatocytes**		
	$t_{1/2}$ (min)	Cl_i (μL/min/mg protein)	
		Mean	SE
**8l**	166.9	8.3	-
**Compound**	**Metabolic Stability in Dog Hepatocytes**		
	$t_{1/2}$ (min)	Cl_i (μL/min/mg protein)	
		Mean	SE
**8l**	>216.8	<6.4	-
**Compound**	**Metabolic Stability in Human Hepatocytes**		
	$t_{1/2}$ (min)	Cl_i (μL/min/mg protein)	
		Mean	SE
**8l**	>216.8	<6.4	-

Pharmacokinetic studies

Mouse pharmacokinetic (PK) data was generated at Charles River Laboratories (Groningen, Netherlands) and GVK Biosciences (Hyderabad, India). Rat PK was performed by WuXi AppTec (Shanghai, China).

Animal Testing Declaration: All in life phase studies were conducted in accordance with local Ethics Review process. See, statements from each company.

Studies:

A. Table S10. Plasma and brain exposure study of 7y following oral administration in male C57/BL6 mice (10 mg/kg):

**Study Title:**	Plasma and Brain Exposure study of ARUK3001043 Following Oral administration in Male C57/BL6 Mice.					
**Study Details**						
**Study Number**	1994-18-DMPK					
**Test Article Name (PO)**	ARUK3001043					
**Formulation PO solution**	0.1% Tween-80 in Water					
**Species**	Male C57/BL6 Mice					
**Study Design**	Single dose BBB Pharmacokinetic Study					
**Matrix**	Plasma and Brain					
**Bioanalytical Details**						
**Analyte**						
**LLOQ (ng/mL) Plasma**	1.00		**ULOQ (ng/mL)**	1000.00		
**LLOQ (ng/mL) Brain**	1.00		**ULOQ (ng/mL)**	1000.00		
**Plasma concentration (ng/mL) of ARUK3001043 (PO- 10 mg/kg**						
**Time (h)**	**M1**	**M2**	**M3**	**Mean**	**Std Dev**	**% CV**
0.17	288.66	304.94	309.74	**301.11**	11.05	3.67
0.50	96.08	106.46	74.15	**92.23**	16.50	17.89
1.00	36.06	17.95	56.00	**36.67**	19.03	51.90
2.00	9.76	9.72	9.13	**9.54**	0.35	3.70
4.00	3.62	6.16	1.95	**3.91**	2.12	54.22
8.00	BLQ	BLQ	BLQ	**BLQ**	NC	NC
24.00	BLQ	BLQ	BLQ	**BLQ**	NC	NC
Dose (mg/kg)	10.00	10.00	10.00	**10.00**	-	-
Cmax(ng/mL)	-	-	-	**301.11**	-	-
Tmax (hr)	-	-	-	**0.17**	-	-
$t_{1/2}$ (h) PO	-	-	-	**0.82**	-	-
$AUC_{0\text{-}last}$ (ng·h/mL)	-	-	-	**146.74**	-	-
$AUC_{0\text{-}inf}$ (ng·h/mL)	-	-	-	**151.35**	-	-
AUC_{Extra}(%)	-	-	-	**3.05**	-	-
$MRT_{0\text{-}last}$ (h)	-	-	-	**0.73**	-	-
Rsq	-	-	-	**0.98**	-	-
**Brain concentration (ng/g) of ARUK3001043 (PO-10 mg/kg)**						
**Time (h)**	**M1**	**M2**	**M3**	**Mean**	**Std Dev**	**% CV**
0.17	641.55	496.35	466.70	**534.87**	93.57	17.49
0.50	96.75	140.10	58.05	**98.30**	41.05	41.76
1.00	37.50	17.20	33.40	**29.37**	10.73	36.55
2.00	6.25	6.70	10.60	**7.85**	2.39	30.47
4.00	BLQ	BLQ	BLQ	**BLQ**	NC	NC
8.00	BLQ	BLQ	BLQ	**BLQ**	NC	NC
24.00	BLQ	BLQ	BLQ	**BLQ**	NC	NC
Dose (mg/kg)	10.00	10.00	10.00	**10.00**	-	-
Cmax(ng/g)	-	-	-	**534.87**	-	-
Tmax (hr)	-	-	-	**0.17**	-	-
$t_{1/2}$ (h) PO	-	-	-	**0.42**	-	-
$AUC_{0\text{-}last}$ (ng·h/g)	-	-	-	**175.35**	-	-
$AUC_{0\text{-}inf}$ (ng·h/g)	-	-	-	**180.15**	-	-
AUC_{Extra}(%)	-	-	-	**2.67**	-	-
$MRT_{0\text{-}last}$ (h)	-	-	-	**0.43**	-	-
Rsq	-	-	-	**0.97**	-	-
Brain to plasma ratio				**1.19**		

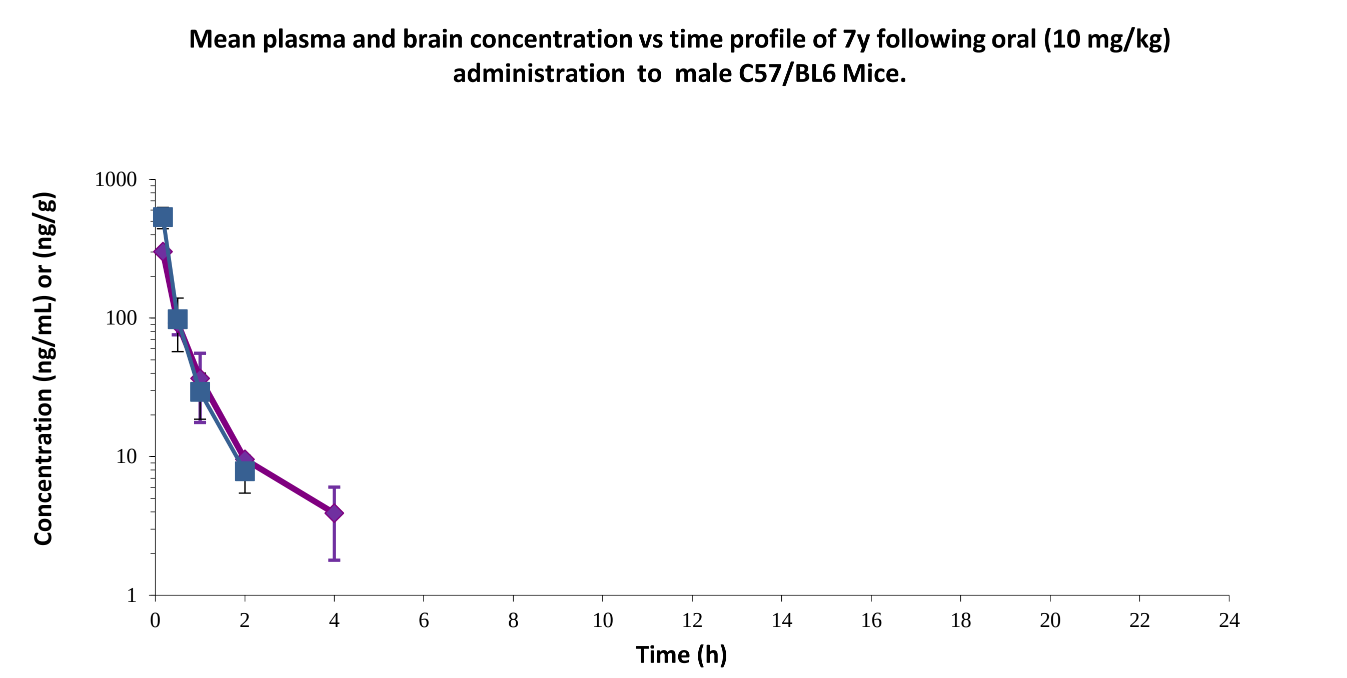

Figure S2. Mean plasma and brain concentration vs time profile of **7y** following oral (10 mg/kg) administration to male C57/BL6 mice. Plasma concentration (ng/mL) as purple diamonds, brain concentration (ng/mL) as blue squares. ARUK3001043 is the compound identifier for **7y**.

B. Table S11. Plasma and brain exposure study of 8l following intravenous administration in male C57/BL6 mice (1 mg/kg):

**Study Title:**	PLASMA AND BRAIN EXPOSURE STUDY OF ARUK3001185 FOLLOWING INTRAVENOUS ADMINISTRATION IN MALE C57BL6 MICE		
**Study Details**			
**Study Number**	0793-19-DMPK		
**Test Article Name (IV)**	ARUK3001185		
**Formulation (IV)**	DMSO (2%v/v) + 10%v/v Solutol in PBS (98%v/v)		
**Species**	C57BL/6 mice		
**Study Design**	IV Study and Brain collection		
**Molecular weight**	282.10		
**Matrix**	Plasma and Brain		
**Bioanalytical Details**			
**Analyte**	ARUK3001185		
**LLOQ (ng/mL)- Plasma**	1.03	**ULOQ (ng/mL)**	1029.60
**LLOQ (ng/mL)- Brain**	1.03	**ULOQ (ng/mL)**	1029.60

**Plasma concentration (ng/mL) of ARUK3001185 following intravenous administration in Male C57BL/6 Mice - (1.00 mg/kg)**						
**Time (h)**	**M-1**	**M-2**	**M-3**	**Mean**	**Std Dev**	**% CV**
0.17	547.86	538.93	597.21	**561.33**	31.39	5.59
0.50	439.71	477.93	448.68	**455.44**	19.99	4.39
1.00	342.41	356.87	549.82	**416.37**	115.80	27.81
2.00	217.46	209.12	209.74	**212.11**	4.65	2.19
4.00	132.12	144.29	149.45	**141.95**	8.90	6.27
8.00	33.00	39.37	46.75	**39.71**	6.88	17.33
24.00	BLQ	BLQ	BLQ	**BLQ**	NA	NA
Dose (mg/kg)	1.00	1.00	1.00	**1.00**	-	-
C0 (ng/mL)	-	-	-	**623.38**	-	-
T1/2 (h)	-	-	-	**2.43**	-	-
AUC0-last (ng·h/mL)	-	-	-	**1458.57**	-	-
AUC0-inf (ng·h/mL)	-	-	-	**1597.95**	-	-
AUCExtra(%)	-	-	-	**8.72**	-	-
Cl (ml/min/kg)	-	-	-	**10.43**	-	-
Vd (L/kg)	-	-	-	**2.20**	-	-
Vss (L/kg)	-	-	-	**1.99**	-	-
MRT0-last (h)				**2.39**		
Rsq	-	-	-	**0.9897**	-	-

Brain concentration (ng/g) of ARUK3001185 following Intravenous administration in Male C57BL/6 Mice- (1.00 mg/kg)						
**Time (h)**	**M-1**	**M-2**	**M-3**	**Mean**	**Std Dev**	**% CV**
0.17	1199.15	818.55	969.00	**995.57**	191.69	19.25
0.50	575.95	682.70	514.20	**590.95**	85.25	14.43
1.00	588.40	374.05	565.40	**509.28**	117.68	23.11
2.00	241.35	423.80	501.50	**388.88**	133.54	34.34
4.00	306.95	271.35	312.45	**296.92**	22.31	7.51
8.00	41.65	96.95	84.80	**74.47**	29.06	39.03
24.00	BLQ	BLQ	BLQ	**BLQ**	NA	NA
Dose (mg/kg)	10.00	10.00	10.00	**1.00**	-	-
C0 (ng/g)	-	-	-	**1293.21**	-	-
T1/2 (h)	-	-	-	**2.58**	-	-
AUC0-last (h*ng/g)	-	-	-	**2494.30**	-	-
AUC0-inf (h*ng/g)	-	-	-	**2771.41**	-	-
AUCExtra(%)	-	-	-	**10.00**	-	-
Cl (g/h/kg)				**360.83**		
Vd (g/kg)	-	-	-	**1342.74**	-	-
Vss (g/kg)	-	-	-	**1275.45**	-	-
MRT0-last (h)	-	-	-	**2.6253**	-	-
Rsq	-	-	-	**0.9776**	-	-

Brain/ Plasma						
**Time (h)**	**M-1**	**M-2**	**M-3**	**Mean**	**Std Dev**	**% CV**
0.17	2.19	1.52	1.62	**1.78**	0.36	20.30
0.50	1.31	1.43	1.15	**1.29**	0.14	10.95
1.00	1.72	1.05	1.03	**1.26**	0.39	31.05
2.00	1.11	2.03	2.39	**1.84**	0.66	35.83
4.00	2.32	1.88	2.09	**2.10**	0.22	10.55
8.00	1.26	2.46	1.81	**1.85**	0.60	32.55
24.00	NA	NA	NA	**NA**	NA	NA

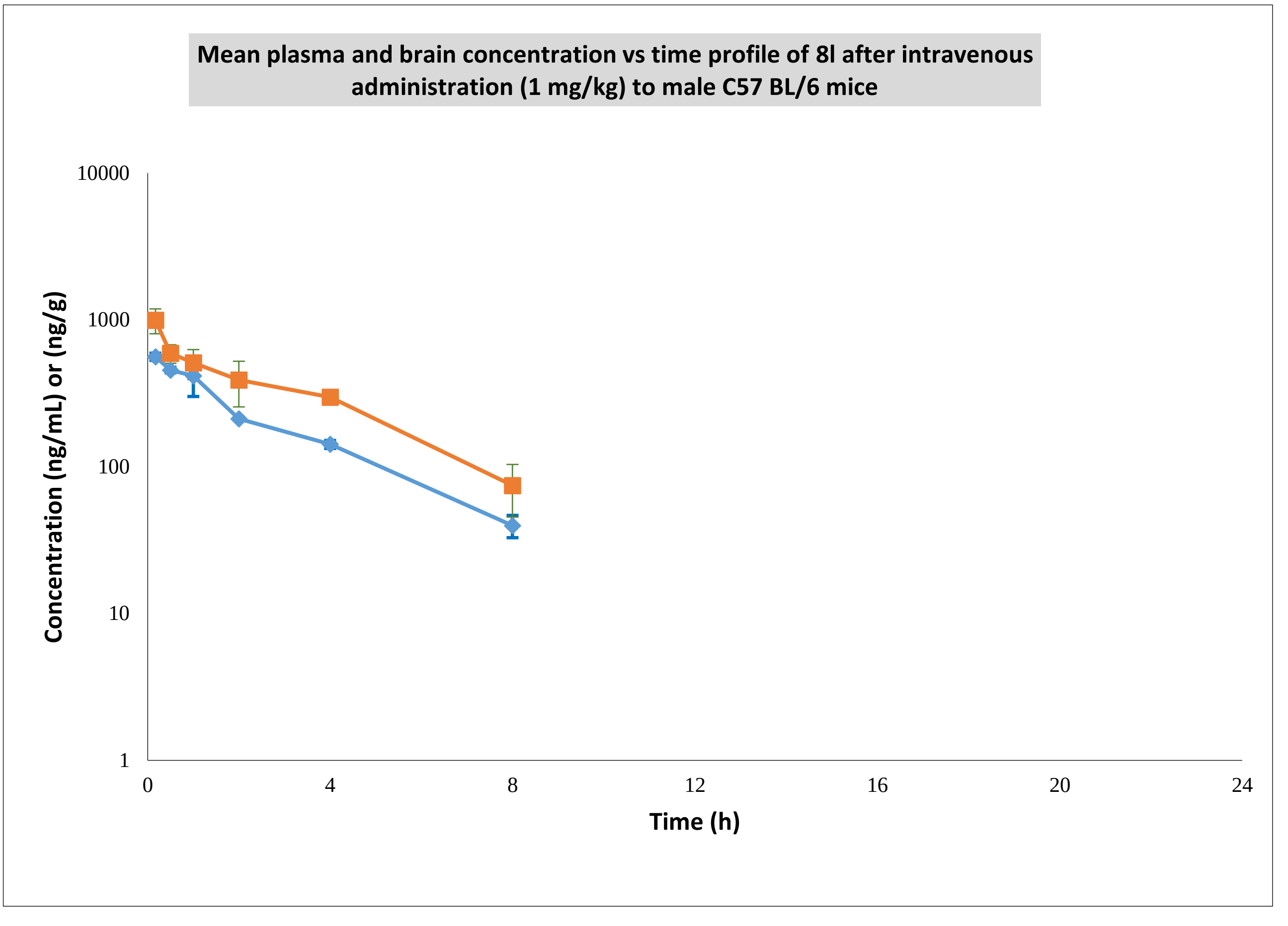

Figure S3. Mean plasma concentration vs time profile of **8l** following intravenous (1 mg/kg) administration to male C57/BL6 mice. Plasma concentration (ng/mL) as blue diamonds, brain concentration (ng/mL) as orange squares. All animals were found healthy throughout the study period. ARUK3001185 is the compound identifier for **8l**.

C. **Table S12. Plasma and brain exposure study of 8l following oral administration in male C57/BL6 mice (10 mg/kg):**

Study Title:	Plasma and Brain Exposure study of ARUK3001185 Following Oral administration in Male C57/BL6 Mice.		
**Study Details**			
**Study Number**	0029-19-DMPK		
**Test Article Name (PO)**	ARUK3001185		
**Formulation PO solution**	0.1% Tween-80 in Water		
**Species**	Male C57/BL6 Mice		
**Study Design**	Single dose BBB Pharmacokinetic Study		
**Matrix**	Plasma and Brain		
**Bioanalytical Details**			
**Analyte**	ARUK3001185		
**LLOQ (ng/mL) Plasma**	1.03	**ULOQ (ng/mL)**	1029.60
**LLOQ (ng/mL) Brain**	1.03	**ULOQ (ng/mL)**	1029.60

Plasma concentration (ng/mL) of ARUK3001185 (PO- 10 mg/kg						
**Time (h)**	**M1**	**M2**	**M3**	**Mean**	**Std Dev**	**% CV**
0.17	1295.66	1068.69	1413.67	**1259.34**	175.33	13.92
0.50	2504.18	2543.30	1881.00	**2309.49**	371.60	16.09
1.00	2427.78	2187.05	1835.06	**2149.96**	298.10	13.87
2.00	1865.73	1864.48	2366.98	**2032.40**	289.76	14.26
4.00	892.23	1062.93	1129.97	**1028.38**	122.58	11.92
8.00	387.71	457.24	326.35	**390.43**	65.49	16.77
24.00	BLQ	3.21	3.42	**3.32**	0.15	4.48
Dose (mg/kg)	10.00	10.00	10.00	**10.00**	-	-
Cmax(ng/mL)	-	-	-	**2309.49**	-	-
Tmax (hr)	-	-	-	**0.50**	-	-
$t_{1/2}$ (h) PO	-	-	-	**2.39**	-	-
$AUC_{0\text{-}last}$ (ng·h/mL)	-	-	-	**10782.23**	-	-
$AUC_{0\text{-}inf}$ (ng·h/mL)	-	-	-	**10793.64**	-	-
AUC_{Extra}(%)	-	-	-	**0.11**	-	-
$MRT_{0\text{-}last}$ (h)	-	-	-	**3.92**	-	-
Rsq	-	-	-	**0.999**	-	-

Brain concentration (ng/g) of ARUK3001185 (PO-10 mg/kg)						
**Time (h)**	**M1**	**M2**	**M3**	**Mean**	**Std Dev**	**% CV**
0.17	2721.50	1863.50	373.95	**1652.98**	1187.85	71.86
0.50	3086.60	2529.95	1949.40	**2521.98**	568.64	22.55
1.00	3411.20	2645.85	2619.50	**2892.18**	449.67	15.55
2.00	2984.95	2393.80	1421.05	**2266.60**	789.67	34.84
4.00	1064.30	1222.60	1015.10	**1100.67**	108.42	9.85
8.00	376.65	364.00	277.45	**339.37**	53.99	15.91
24.00	BLQ	BLQ	BLQ	**BLQ**	NC	NC
Dose (mg/kg)	10.00	10.00	10.00	**10.00**	-	-
Cmax(ng/g)	-	-	-	**2892.18**	-	-
Tmax (hr)	-	-	-	**1.00**	-	-
$t_{1/2}$ (h) PO	-	-	-	**2.21**	-	-
$AUC_{0\text{-}last}$ (ng·h/g)	-	-	-	**10565.87**	-	-
$AUC_{0\text{-}inf}$ (ng·h/g)	-	-	-	**11649.07**	-	-
AUC_{Extra}(%)	-	-	-	**9.30**	-	-
$MRT_{0\text{-}last}$ (h)	-	-	-	**2.74**	-	-
Rsq	-	-	-	**0.997**	-	-
**Brain to Plasma Ratio (PO)**						
**Time (h)**	**M1**	**M2**	**M3**	**Mean**	**Std Dev**	**% CV**
0.17	2.10	1.74	0.26	**1.37**	0.97	71.08
0.50	1.23	0.99	1.04	**1.09**	0.13	11.68
1.00	1.41	1.21	1.43	**1.35**	0.12	8.89
2.00	1.60	1.28	0.60	**1.16**	0.51	43.99
4.00	1.19	1.15	0.90	**1.08**	0.16	14.73
8.00	0.97	0.80	0.85	**0.87**	0.09	10.29
24.00	NC	NC	NC	**NC**	NC	NC

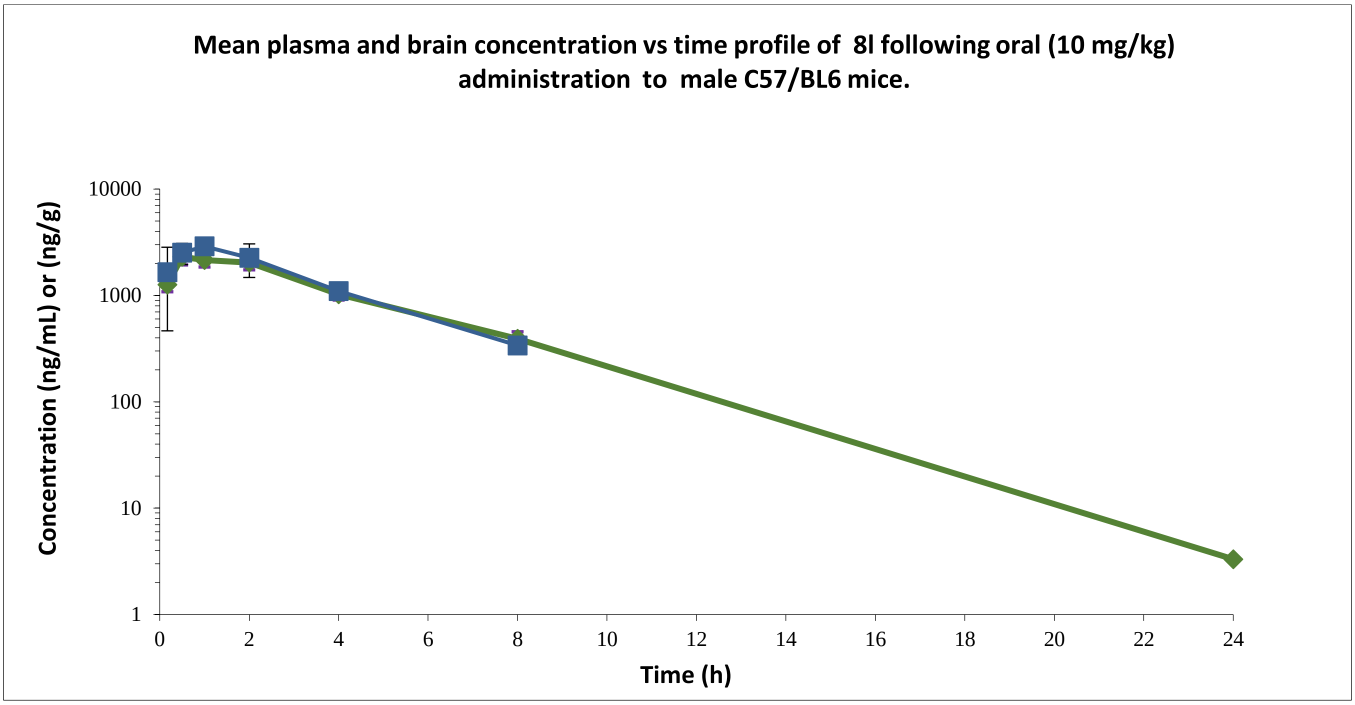

Figure S4. Mean plasma concentration vs time profile of **8l** following oral (10 mg/kg) administration to male C57/BL6 mice. Plasma concentration (ng/mL) as green diamonds, brain concentration (ng/mL) as blue squares. ARUK3001185 is the compound identifier for **8l**.

D. Table S13. Plasma and brain exposure study of 8l following oral administration in mouse (2x30 mg/kg):

**Plasma analysis (n = 3)** LLOQ: 20 ng/mL	
**Timepoint** (hr)	**Concentration 8l** (ng/mL)
48	< LLOQ
48	< LLOQ
48	< LLOQ
24	295
24	362
24	29.9
11	6851
11	6221
11	5603
10	3803
10	4590
10	6266
9.5	7569
9.5	7935
9.5	8252
9.25	5941
9.25	5797
9.25	6213
9	645
9	738
9	756
4	3179
4	4727
4	4901
2	3997
2	4816
2	3899
1	4916

1	4546
1	3715
0.5	5087
0.5	4736
0.5	5717
0.25	2890
0.25	3139
0.25	4027

**Brain analysis (n = 3)** LLOQ: 30 ng/g	
**Timepoint** (hr)	**Concentration 8l** (ng/g)
48	< LLOQ
48	< LLOQ
48	< LLOQ
24	352
24	498
24	32.2
10	7254
10	6540
10	8256

**Protocol and PK Parameters**	
Dose level (mg/kg)	30
Formulation	DMSO (2% v/v) + 10% kolliphor HS 15 in PBS (98% v/v)
Mouse	3x male
Route of administration	po by gavage
T_{last} (h)	24
T_{max} (h)	9.5
C_{max} (ng/mL)	7920
AUC_{last} (h*ng/mL)	82400
AUC_{inf} (h*ng/mL)	83400
%extrapolated (%)	1
$t_{1/2}$ (h)	2.97
r^2	0.9832

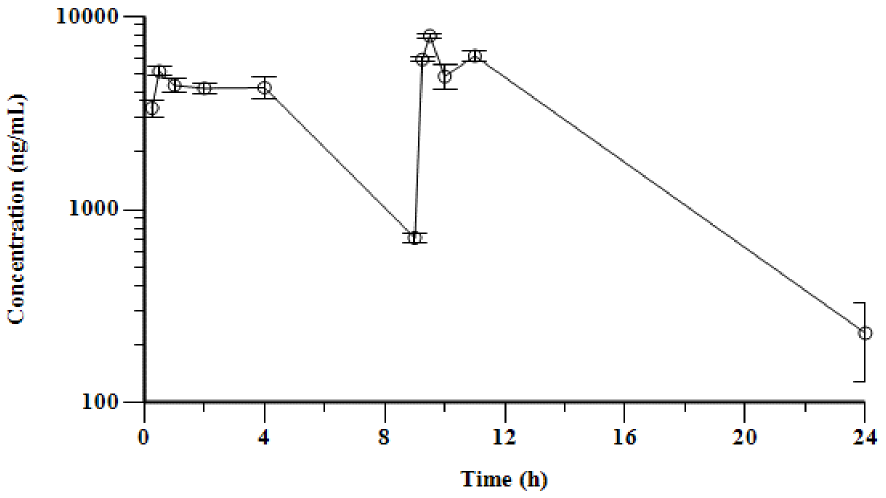

Figure S5. Mean plasma concentration vs time profile of **8l** following oral (2x30 mg/kg) administration to mouse. Plasma concentration (ng/mL) as open circles (○). Dose at T = 0 and 9 h. No clinical signs were noted during clinical observation.

E. Table S14. Plasma and brain exposure study of 8l following intravenous (1 mg/kg) and oral (10 mg/kg) administration in male SD rat:

Study Details		
**Sponsor**	409307	
**Sponsor Study No.**	NA	
**WuXi DMPK Study No.**	409307-20210916-RPK	
**Species**	Male SD Rat, fasted for PO.	
**Administered compound**	ARUK3001185	ARUK3001185
**Study Group**	IV bolus1	PO2
**Nominal dose (mg/kg)**	1.00	10.0
**Administered dose (mg/kg)**	0.845	8.57
**Formulation**	0.50 mg/mL in DMSO (2%v/v) + 10% v/v Solutol in 1X PBS (98% v/v), clear solution	1 mg/mL in DMSO (2%v/v) + 10% v/v Solutol in 1X PBS (98% v/v), Nearly clear solution

Individual and Mean Concentration of ARUK3001185							
IV bolus1							
Plasma concentration (ng/mL)							
**Time (h)**	**R01**	**R02**	**R03**	**Mean**		**SD**	**CV (%)**
**0.0830**	712	657	699	689	±	28.7	4.17
**0.250**	564	543	479	529	±	44.3	8.37
**0.500**	464	487	418	456	±	35.1	7.70
**1.00**	416	370	470	419	±	50.1	12.0
**2.00**	262	270	295	276	±	17.2	6.24
**4.00**	217	175	209	200	±	22.3	11.1
**8.00**	84.2	78.8	85.0	82.7	±	3.37	4.08
**24.0**	BQL	BQL	BQL	ND	±	ND	ND
Brain concentration (ng/g)							
**Time (h)**	**R01**	**R02**	**R03**	**Mean**		**SD**	**CV (%)**
**24.0**	BQL	BQL	BQL	ND	±	ND	ND

PK Parameters of ARUK3001185 (ng/mL)							
IV bolus1							
Plasma							
**PK Parameters**	**R01**	**R02**	**R03**	**Mean**		**SD**	**CV (%)**
**Rsq_adj**	0.967	0.999	0.992	--	±	--	--
**No. points used for $T_{1/2}$**	5.00	3.00	3.00	ND	±	--	--
**C_0 (ng/mL)**	799	722	843	788	±	61.2	7.77
**$T_{1/2}$ (h)**	3.15	3.39	3.30	3.28	±	0.121	3.70
**Vd_{ss} (L/kg)**	1.96	2.16	1.95	2.02	±	0.118	5.85
**Cl(mL/min/kg)**	7.34	7.85	7.17	7.45	±	0.354	4.75
**T_{last} (h)**	8.00	8.00	8.00	8.00	±	--	--
**AUC_{0-last} (ng.h/mL)**	1888	1736	1921	1848	±	98.7	5.34
**AUC_{0-inf} (ng.h/mL)**	2271	2122	2326	2240	±	106	4.71
**MRT_{0-last} (h)**	2.82	2.73	2.81	2.79	±	0.0493	1.77
**MRT_{0-inf} (h)**	4.46	4.58	4.54	4.53	±	0.0611	1.35
**AUC_{Extra} (%)**	16.8	18.2	17.4	17.5	±	0.702	4.02
**$AUMC_{Extra}$ (%)**	47.4	51.2	49.0	49.2	±	1.91	3.88

PO2							
Plasma concentration (ng/mL)							
**Time (h)**	**R1+3n**	**R2+3n**	**R3+3n**	**Mean**		**SD**	**CV (%)**
**0.0830**	718	622	950	763	±	169	22.1
**0.250**	2930	2440	3100	2823	±	343	12.1
**0.500**	2630	2190	3070	2630	±	440	16.7
**1.00**	1660	1680	1470	1603	±	116	7.23
**2.00**	1930	1950	1590	1823	±	202	11.1
**4.00**	2210	3820	3740	3257	±	907	27.9
**8.00**	1310	2220	2260	1930	±	537	27.8
**24.0**	247	214	73.5	178	±	92.1	51.7
Brain concentration (ng/g)							
**Time (h)**	**R1+3n**	**R2+3n**	**R3+3n**	**Mean**		**SD**	**CV (%)**
**0.500**	3810	3306	3846	3654	±	302	8.26
**1.00**	2502	2700	2268	2490	±	216	8.68
**2.00**	3444	4038	3054	3512	±	496	14.1
**24.0**	696	660	169	508	±	294	57.9
Brain/Plasma Ratio							
**Time (h)**	**R1+3n**	**R2+3n**	**R3+3n**	**Mean**		**SD**	**CV (%)**
**0.500**	1.45	1.51	1.25	1.40	±	0.134	9.56
**1.00**	1.51	1.61	1.54	1.55	±	0.0506	3.26
**2.00**	1.78	2.07	1.92	1.93	±	0.143	7.44
**24.0**	2.82	3.08	2.30	2.73	±	0.399	14.6

PK Parameters of ARUK3001185		
PK Parameters	PO2	
	Mean Brain	Mean Plasma
Rsq_adj	0.916	0.999
No. points used for $T_{1/2}$	3.00	3.00
C_{max} (ng/mL or ng/g)	3654	3257
T_{max} (h)	0.500	4.00
$T_{1/2}$ (h)	8.92	4.74
T_{last} (h)	24.0	24.0
AUC_{0-last} (ng.h/mL or ng.h/g)	39584	30768
AUC_{0-inf} (ng.h/mL or ng.h/g)	46125	31984
MRT_{0-last} (h)	8.49	7.58
MRT_{0-inf} (h)	12.5	8.47
AUC_{Extra} (%)	14.2	3.80
$AUMC_{Extra}$ (%)	41.8	13.8
Bioavailability (%)	--	143
[a]AUC Ratio	1.29	--

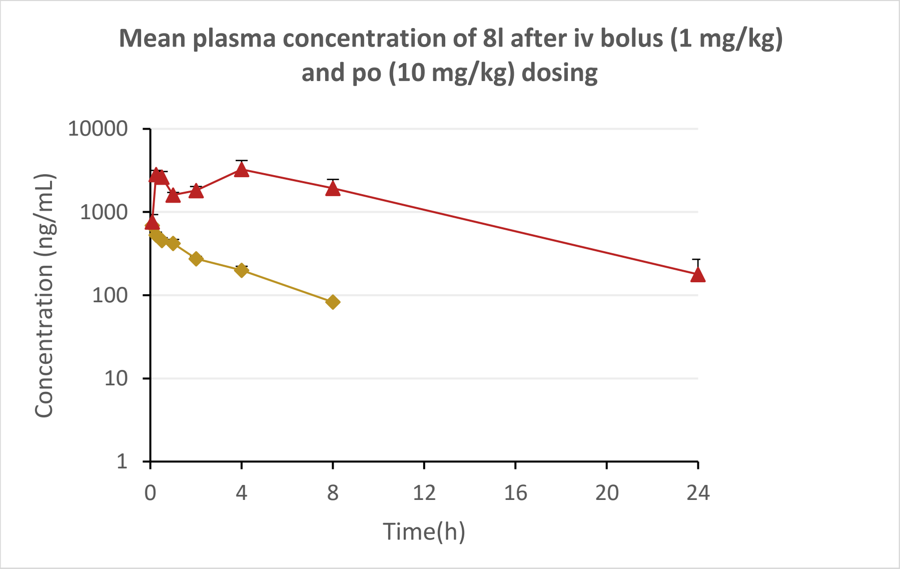

Figure S6. Mean plasma concentration vs time profile of **8l** following intravenous (1 mg/mL) and oral (10 mg/kg) administration to male SD rat. Intravenous profile as brown diamonds and oral profile as red triangles. ARUK3001185 is the compound identifier for **8l**.

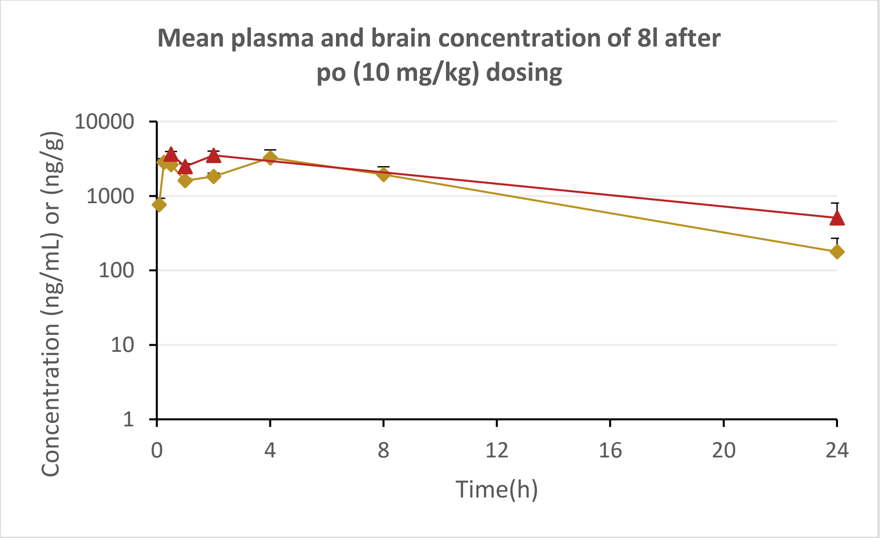

Figure S7. Mean plasma and brain concentration vs time profile of **8l** following oral (10 mg/kg) administration to male SD rat. Plasma concentration (ng/mL) as brown diamonds and brain concentration (ng/g) as red triangles. Animal clinical observation record reported all animals normal.

F. Pilot formulation studies with 8l:

Table S15. Results:

Dose Formulation 8l		Mean Concentration (mg/mL)	Observations
20% w/v HPßCD in Water	Replicate-1	1.00	Formulation was with particles; after probe sonication, formulation changed into hazy solution.
	Replicate-2		
	Replicate-3		
DMSO (2%v/v) + 10% v/v Solutol in PBS (98% v/v)	Replicate-1	0.96	Formulation was clear and transparent in appearance.
	Replicate-2		
	Replicate-3		
DMSO (2%v/v) + 10% v/v HPßCD in WFI (98% v/v/)	Replicate-1	1.02	Formulation was clear and transparent in appearance.
	Replicate-2		
	Replicate-3		

G. Plasma protein binding (PPB):

Method: rapid equilibrium dialysis.

Table S16. Protocol:

Test System	Mouse Plasma (K2EDTA)<br>Rat Plasma (SD)
Test compound concentration	10 µM (mouse) or 2 µM (rat)
Incubation Time	5 h (mouse) or 4 h (rat)
No of Replicates	Two
Buffer	Phosphate buffer saline, pH 7.4
Bioanalysis	LC-MS/MS

Table S17. Results:

Compound	Species / Matrix	% Unbound in Plasma Mean Value	SD	% Bound in Plasma	% Recovery
**8l**	Mouse plasma	4.20	0.16	95.80	89.87
Warfarin	Mouse plasma	4.29	0.47	95.71	102.69
**8l**	Rat plasma	5.52	1.0	94.48	99.27
Warfarin	Rat plasma	0.63	0.0	99.37	100.02

H. Brain tissue binding:

Method: rapid equilibrium dialysis.

Table S18. Protocol:

Test System	Mouse Brain Homogenate 10% w/v in PBS<br>Rat Brain homogenate
Test compound concentration	10 μM (mouse) or 2 μM (rat)
Incubation Time	5 h (mouse) or 4 h (rat)
No. of Replicates	Two (mouse) or three (rat)
Buffer	Phosphate buffer saline, pH 7.4
Bioanalysis	LC-MS/MS

Table S19. Results:

Compound	Species / Matrix	% Unbound in brain homogenate Mean Value	SD	% Bound in brain homogenate	% Recovery
**8l**	Mouse Brain Homogenate	2.98	0.63	97.02	106.81
Diazepam	Mouse Brain Homogenate	3.97	0.30	96.03	102.41
**8l**	SD Rat Brain Homogenate	2.9	0.2	97.1	86.8
Propranolol	SD Rat Brain Homogenate	2.6	0.1	97.4	78.2

Notum OPTS and TCF/LEF screening data concentration-response curves for 8l

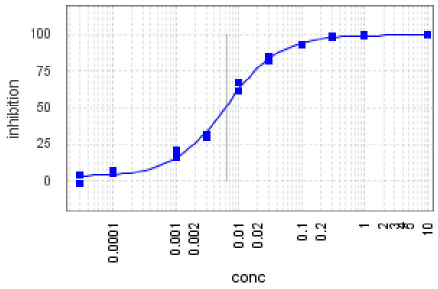

IC$_{50}$ 6.4 nM
[Ref: 150150]

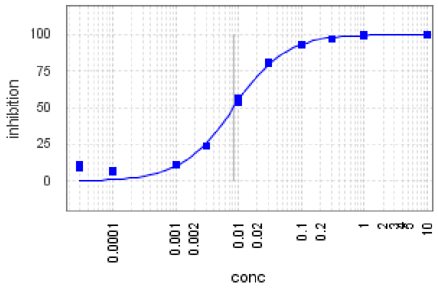

IC$_{50}$ 8.2 nM
[Ref: 148048]

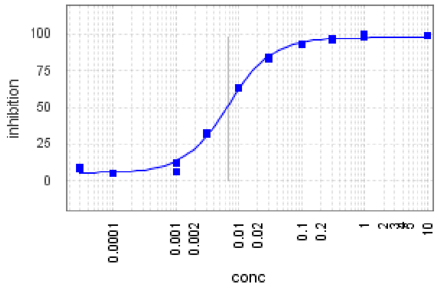

IC$_{50}$ 6.7 nM
[Ref: 145841]

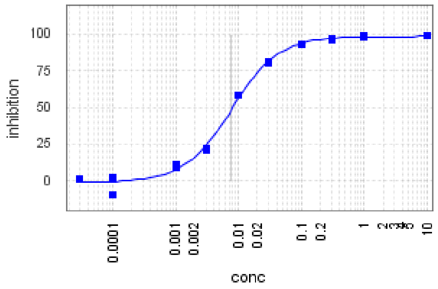

IC$_{50}$ 7.7 nM
[Ref: 145836]

Figure S8. Notum OPTS data. Inhibition-concentration curves are representative, medium examples for **8l** (4 of 22 shown) from the larger data set for compounds **6-9**. Examples are from different screening runs.

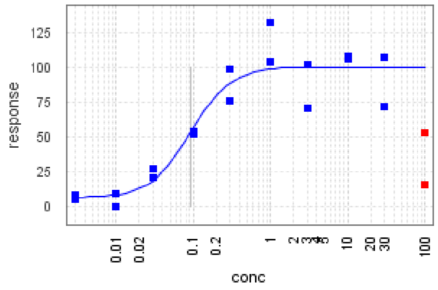

EC$_{50}$ 92.3 nM
[Ref: 146034]

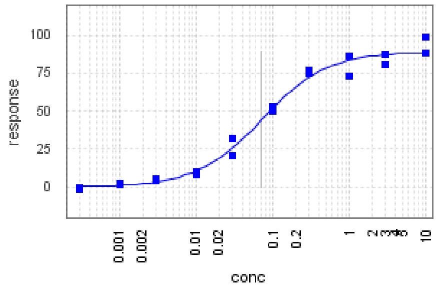

EC$_{50}$ 71.8 nM
[Ref: 146158]

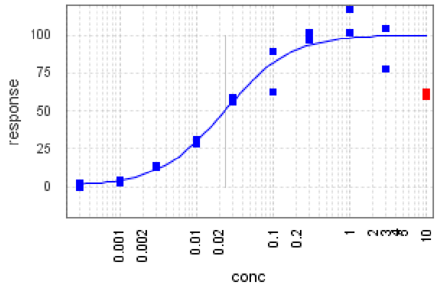

EC$_{50}$ 23.9 nM
[Ref: 146220]

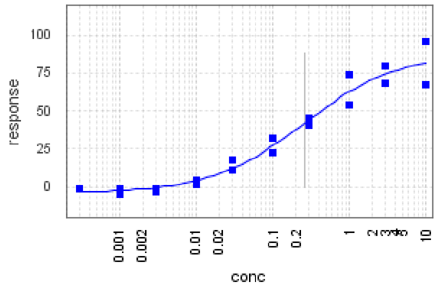

EC$_{50}$ 256 nM
[Ref: 146411]

Figure S9. Notum TCF/LEF reporter (Luciferase) data with WNT3A (100 ng/mL). Response-concentration curves are for **8l** from a larger data set for compounds **6-9**. Examples are from different screening runs.

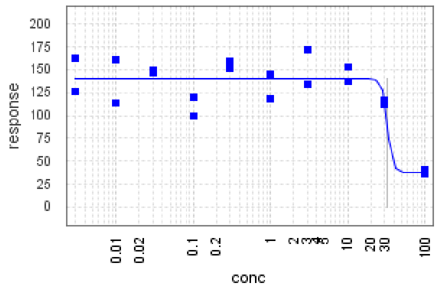

[Ref: 146037]

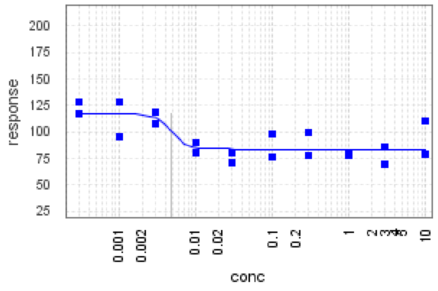

[Ref: 146153]

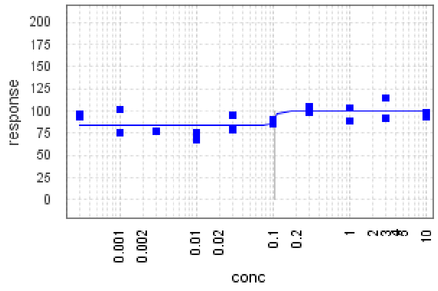

[Ref: 146159]

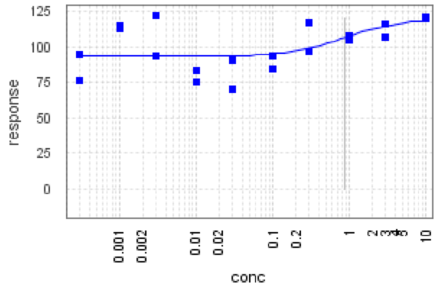

[Ref: 146415]

Figure S10. Notum TCF/LEF reporter (Luciferase) data with WNT3A (100 ng/mL) in the *absence* of Notum. Response-concentration curves are for **8l** from a larger data set for compounds **6-9**. Examples are from different screening runs.

Additional inhibition-concentration curves for compounds **6-9** will be made available upon reasonable request.

Activity-based protein profiling optimization

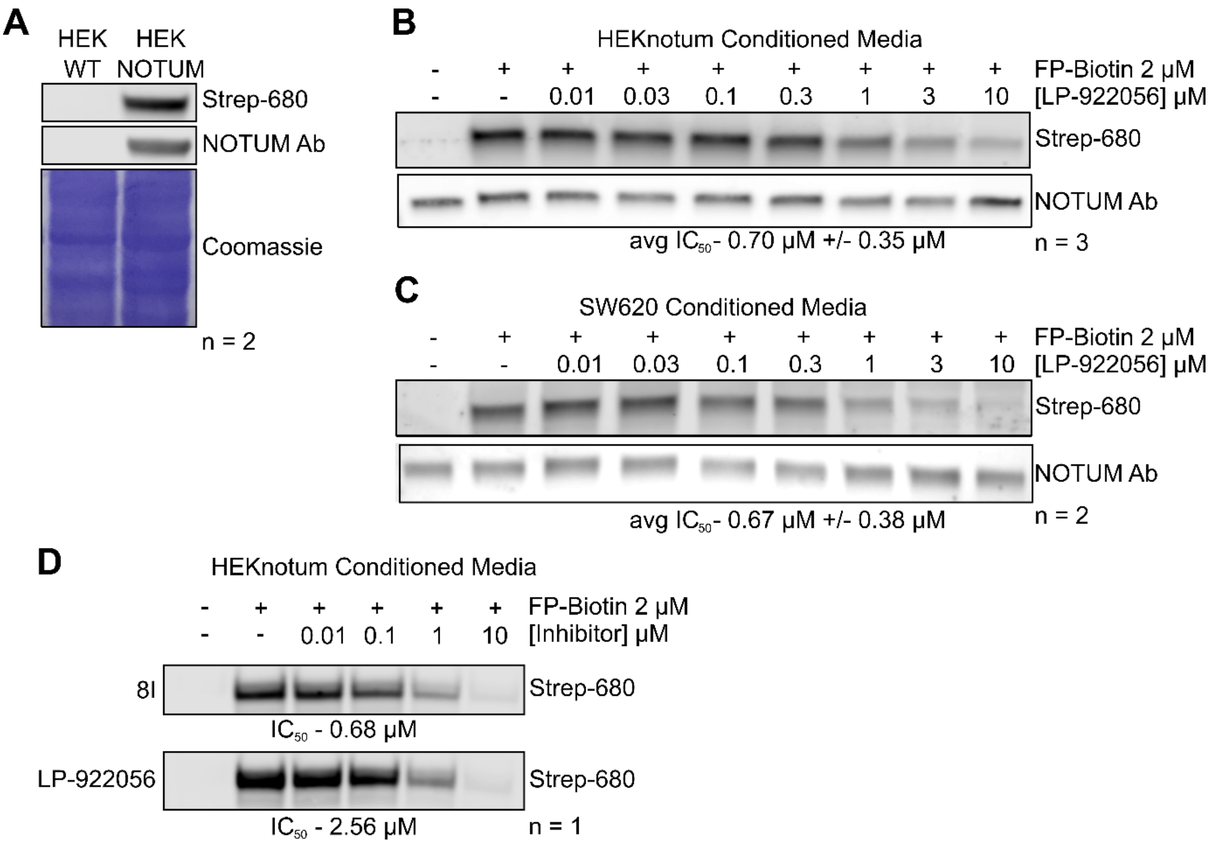

Figure S11. (A) Notum was identified using both FP-biotin and a Notum antibody in the condition media of HEK293S cells transduced with a high expression Notum transgene. (B-C) Concentration-response curves of Notum inhibitor LP-922056 (**1**) can be generated using conditioned media from this cell line, or from the colorectal cancer line SW620, by treating with FP-biotin to label active serine hydrolases. This labelling can be blocked by inhibitors and IC_{50} values calculated by determining the level of biotin labelled protein upon increasing concentrations of the inhibitor using a Streptavidin-680 conjugate (Sigma Aldrich). (D) **8l** and **1** were evaluated side-by-side in gel to compare FP-biotin labelling of Notum conditioned media using a 20-minute time point, as used within the proteomics experiment. Timing of the assay is a critical component for defining the IC_{50} values by virtue of having reversible inhibitors with a covalent labelling agent (FP-biotin). Blots imaged on Li-cor Odyssey CLx and analysed using Image Studio Lite with IC_{50} values calculated using Graphpad Prism 9.

Serine hydrolase selectivity of LP-922056 (1) by ABPP-TMT

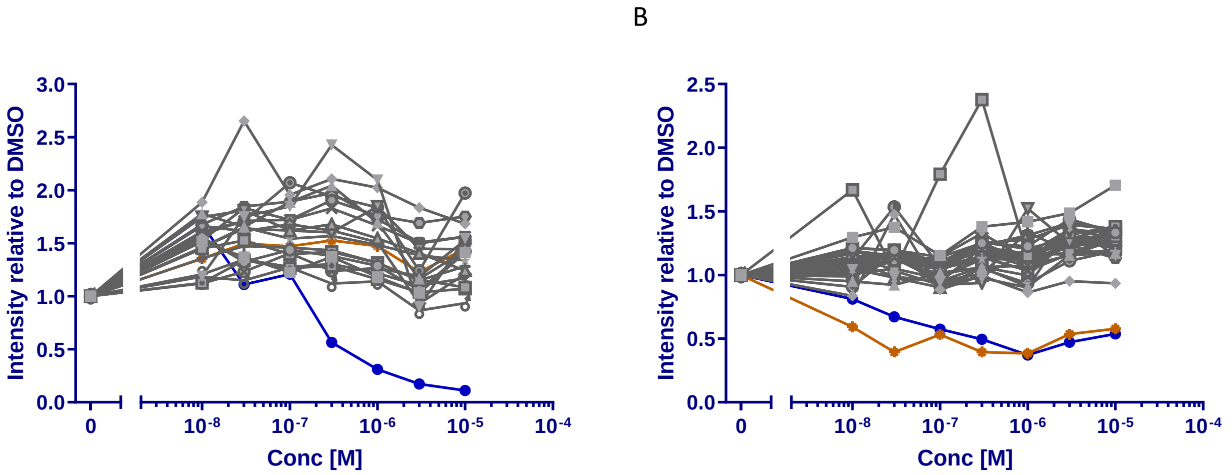

Figure S12. Concentration-response curves from proteomic analysis of LP-922056 (**1**) in SW620 cells. Notum response in dark blue circles, KLK6 as brown circles and all other serine hydrolases detected in gray. (A) Concentrated conditioned media, Notum IC_{50} 250 nM. (B) Cell lysates. Each sample set was pre-incubated with an 8-point concentration-response curve of **1** or DMSO, then labelled for 20 min with FP-Biotin (2 μM) to capture active serine hydrolases. Labelled proteins were enriched on neutravidin agarose then digested using LysC and Trypsin. The subsequent peptides were isobarically labelled with 8 channels of a TMT10plex and combined for final LC-MS/MS analysis.

The identity and quantification of each serine hydrolase identified in SW620 cells by FP-biotin ABPP for **1** (and **8l**) is provided as a Supplementary Information file (XLSX). The mass spectrometry proteomics data have been deposited to the ProteomeXchange Consortium via the PRIDE partner repository with the dataset identifier PXD031338.

Kinase selectivity panel

Kinase selectivity screening was performed by ThermoFisher Scientific (Paisley, U.K.) in their SelectScreen™ Biochemical Kinase Profiling service.

A

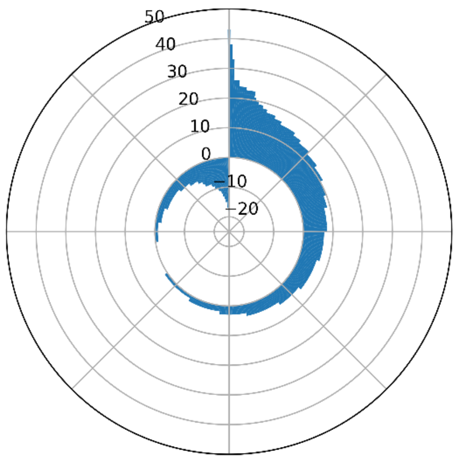

B

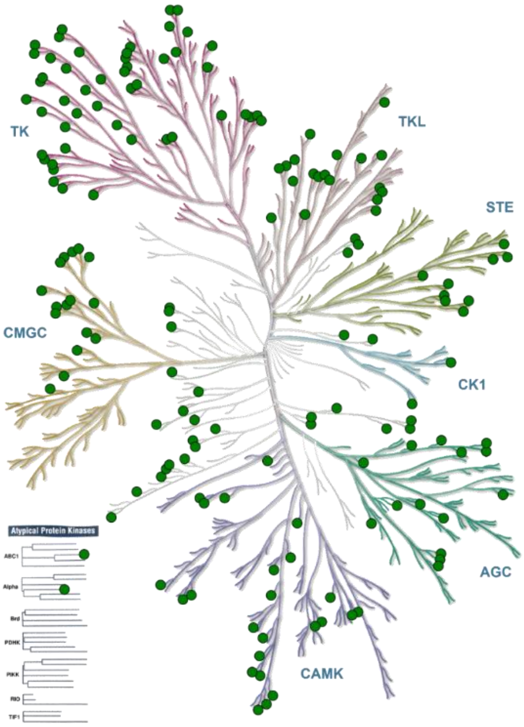

Illustration reproduced courtesy of Cell Signaling technology, Inc.

Figure S13. (A) Radial bar chart of kinase selectivity for **8l** in a panel of 485 distinct kinases showing mean % inhibition @ 10 μM (n = 2). The following kinases showed > 20% inhibition @ 10 μM: ABL1 M351T (24), AMPK (A2/B2/G2) (24), BTK (21), CDK11/cyclin C (23), CDK13/cyclin K (38), CDK14/cyclin y (43), CHEK1 (24), DAPK2 (23), DDR2 N456S (26), KIT D816V (26), LCK (33), MAP 2K1 (26), MST4 (23), PAK1 (23), PRKCZ (23), ZAP70 (24). (B) Illustration of phylogenetic tree of kinases screened.

Table S20. Summary of results.

Kinase Tested	% Inhibition		% Inhibition
	Point 1	Point 2	mean
**SSBK ZLyte SP Technology:**			
ABL1 E255K	14	12	13
ABL1 F317I	13	14	13
ABL1 F317L	17	16	16
ABL1 G250E	8	7	8
ABL1 T315I	0	1	0
ABL1 Y253F	13	14	13
ABL1	17	20	19
ABL2 (Arg)	8	13	10
ACVR1B (ALK4)	-7	-16	-12
ADRBK1 (GRK2)	5	4	4
ADRBK2 (GRK3)	-1	-2	-1
AKT1 (PKB alpha)	7	7	7

AKT2 (PKB beta)	5	8	7
AKT3 (PKB gamma)	6	-1	3
ALK	4	7	5
AMPK (A1/B2/G2)	9	2	6
AMPK (A1/B2/G3)	12	0	6
AMPK (A2/B1/G2)	-2	-2	-2
AMPK (A2/B1/G3)	1	3	2
AMPK (A2/B2/G3)	6	1	3
AMPK A1/B1/G1	25	4	14
AMPK A2/B1/G1	19	9	14
AURKA (Aurora A)	4	3	4
AURKB (Aurora B)	8	5	7
AURKC (Aurora C)	23	15	19
AXL	6	3	4
BLK	8	-4	2
BMX	5	10	7
BRAF V599E	1	9	5
BRAF	6	13	9
BRSK1 (SAD1)	3	-2	0
BTK	25	18	21
CAMK1D (CaMKI delta)	-6	8	1
CAMK1G (CAMKI gamma)	5	-1	2
CAMK2A (CaMKII alpha)	-1	-4	-2
CAMK2B (CaMKII beta)	4	4	4
CAMK2D (CaMKII delta)	4	10	7
CAMK4 (CaMKIV)	15	15	15
CDC42 BPA (MRCKA)	-5	-2	-3
CDC42 BPB (MRCKB)	-1	-1	-1
CDC42 BPG (MRCKG)	6	-2	2
CDK1/cyclin B	5	4	4
CDK17/cyclin Y	2	1	1
CDK18/cyclin Y	7	1	4
CDK2/cyclin A	4	9	6
CDK5/p25	1	1	1
CDK5/p35	10	3	7
CDKL5	-5	-5	-5
CHEK1 (CHK1)	23	25	24
CHEK2 (CHK2)	15	1	8
CLK1	-2	3	0
CLK2	15	22	18
CLK3	3	1	2
CSF1R (FMS)	-4	16	6
CSK	0	3	2
CSNK1A1 (CK1 alpha 1)	3	2	2
CSNK1A1L	-5	2	-2
CSNK1D (CK1 delta)	4	1	3
CSNK1E (CK1 epsilon) R178C	-4	-3	-3
CSNK1E (CK1 epsilon)	-3	4	1
CSNK1G1 (CK1 gamma 1)	3	5	4
CSNK1G2 (CK1 gamma 2)	-2	-1	-2
CSNK1G3 (CK1 gamma 3)	1	-1	0
CSNK2A1 (CK2 alpha 1)	10	0	5
CSNK2A2 (CK2 alpha 2)	14	21	17
DAPK3 (ZIPK)	6	9	8
DCAMKL1 (DCLK1)	4	2	3
DCAMKL2 (DCK2)	0	1	1
DNA-PK	-8	7	0
DYRK1A	-1	0	0
DYRK1B	14	18	16
DYRK3	0	2	1
DYRK4	2	1	1
EEF2K	3	4	3
EGFR (ErbB1) C797S	-11	8	-2
EGFR (ErbB1) G719C	7	3	5
EGFR (ErbB1) G719S	-2	0	-1
EGFR (ErbB1) L858R	2	3	2
EGFR (ErbB1) L861Q	-1	0	0

EGFR (ErbB1) T790M C797S L858R	-28	-7	-17
EGFR (ErbB1) T790M L858R	11	13	12
EGFR (ErbB1) T790M	5	12	9
EGFR (ErbB1)	-4	5	1
EPHA1	4	4	4
EPHA2	6	12	9
EPHA4	-1	2	1
EPHA5	-1	1	0
EPHA8	4	0	2
EPHB1	2	4	3
EPHB2	3	1	2
EPHB3	3	-19	-8
EPHB4	1	2	1
ERBB2 (HER2)	4	-3	0
ERBB4 (HER4)	0	3	1
FER	-2	6	2
FES (FPS)	3	9	6
FGFR1	-1	-5	-3
FGFR2 N549H	27	6	17
FGFR2	13	9	11
FGFR3 K650E	-3	-6	-5
FGFR3 V555M	-20	-7	-14
FGFR3	13	11	12
FGFR4	4	0	2
FGR	3	4	4
FLT1 (VEGFR1)	9	7	8
FLT3 D835Y	4	3	3
FLT3	16	14	15
FLT4 (VEGFR3)	-6	6	0
FRAP1 (mTOR)	4	3	4
FRK (PTK5)	1	1	1
FYN	-3	3	0
GRK4	-3	-3	-3
GRK5	-1	-3	-2
GRK6	1	4	2
GRK7	-1	0	0
GSK3A (GSK3 alpha)	6	9	7
GSK3B (GSK3 beta)	6	7	7
HCK	9	11	10
HIPK1 (Myak)	3	2	2
HIPK2	1	4	2
HIPK3 (YAK1)	4	2	3
HIPK4	-1	-3	-2
IGF1R	1	-9	-4
IKBKB (IKK beta)	-1	-2	-1
IKBKE (IKK epsilon)	8	13	11
INSR	0	13	6
INSRR (IRR)	5	16	11
IRAK4	8	2	5
ITK	-10	2	-4
JAK1	13	-8	2
JAK2 JH1 JH2 V617F	-9	1	-4
JAK2 JH1 JH2	-1	0	-1
JAK2	-2	8	3
JAK3	5	9	7
KDR (VEGFR2)	16	13	14
KIT T670I	7	13	10
KIT V559D V654A	-1	3	1
KIT V559D	-6	0	-3
KIT V560G	-9	15	3
KIT	-3	-12	-7
KSR2	1	4	2
LCK	33	33	33
LTK (TYK1)	3	4	4
LYN A	-4	3	-1
LYN B	6	9	7
MAP2K1 (MEK1)	-5	-5	-5

MAP2K2 (MEK2)	4	9	6
MAP2K6 (MKK6)	14	-7	3
MAP3K19 (YSK4)	-4	-5	-5
MAP3K8 (COT)	-4	4	0
MAP3K9 (MLK1)	-4	-4	-4
MAP4K2 (GCK)	-1	-5	-3
MAP4K4 (HGK)	-10	-5	-8
MAP4K5 (KHS1)	-12	-9	-10
MAPK1 (ERK2)	-4	0	-2
MAPK10 (JNK3)	1	-3	-1
MAPK11 (p38 beta)	5	7	6
MAPK12 (p38 gamma)	1	4	2
MAPK13 (p38 delta)	-2	-1	-2
MAPK14 (p38 alpha) Direct	-3	-3	-3
MAPK14 (p38 alpha)	1	-3	-1
MAPK3 (ERK1)	4	5	4
MAPK7 (ERK5)	-4	-2	-3
MAPK8 (JNK1)	9	2	6
MAPK9 (JNK2)	-5	-28	-17
MAPKAPK2	-1	3	1
MAPKAPK3	2	0	1
MAPKAPK5 (PRAK)	-4	16	6
MARK1 (MARK)	-6	4	-1
MARK2	-3	-11	-7
MARK3	0	5	3
MARK4	-2	-2	-2
MATK (HYL)	-14	-8	-11
MELK	13	11	12
MERTK (cMER)	0	10	5
MET (cMet) Y1235D	0	0	0
MET (cMet)	5	10	8
MET M1250T	-2	8	3
MINK1	15	14	14
MKNK1 (MNK1)	-4	4	0
MST1R (RON)	12	9	11
MST4	24	22	23
MUSK	-2	-5	-4
MYLK2 (skMLCK)	17	14	15
NEK1	-3	5	1
NEK2	-18	-4	-11
NEK4	2	3	3
NEK6	5	20	12
NEK9	-4	-3	-3
NIM1K	4	6	5
NTRK1 (TRKA)	0	6	3
NTRK2 (TRKB)	7	2	5
NTRK3 (TRKC)	14	20	17
PAK1	26	20	23
PAK2 (PAK65)	-3	12	5
PAK3	-4	21	8
PAK4	2	3	2
PAK6	24	5	15
PAK7 (KIAA1264)	4	12	8
PASK	-4	3	-1
PDGFRA (PDGFR alpha)	12	13	13
PDGFRA D842V	-1	1	0
PDGFRA T674I	0	7	4
PDGFRA V561D	3	-8	-3
PDGFRB (PDGFR beta)	19	21	20
PDK1 Direct	0	-4	-2
PDK1	2	6	4
PEAK1	18	23	20
PHKG1	12	5	8
PHKG2	5	11	8
PIM1	10	1	6
PIM2	6	4	5
PIM3	4	9	6

PKN1 (PRK1)	-3	1	-1
PLK1	10	17	13
PLK2	16	15	15
PLK3	2	8	5
PRKACA (PKA)	-4	3	0
PRKCA (PKC alpha)	-13	-24	-18
PRKCB1 (PKC beta I)	-9	9	0
PRKCB2 (PKC beta II)	-16	-18	-17
PRKCD (PKC delta)	-11	3	-4
PRKCE (PKC epsilon)	-13	11	-1
PRKCG (PKC gamma)	15	9	12
PRKCH (PKC eta)	-6	5	-1
PRKCI (PKC iota)	9	-1	4
PRKCN (PKD3)	7	4	6
PRKCQ (PKC theta)	-6	-17	-11
PRKCZ (PKC zeta)	26	20	23
PRKD1 (PKC mu)	7	14	11
PRKD2 (PKD2)	1	-1	0
PRKG1	15	15	15
PRKG2 (PKG2)	10	6	8
PRKX	11	0	6
PTK2 (FAK)	3	5	4
PTK2B (FAK2)	10	3	7
PTK6 (Brk)	6	5	6
RAF1 (cRAF) Y340D Y341D	6	15	10
RET A883F	6	14	10
RET S891A	5	14	10
RET V804E	0	9	4
RET V804L	5	10	8
RET Y791F	4	7	5
RET	4	10	7
ROCK1	4	11	8
ROCK2	21	11	16
ROS1	8	11	9
RPS6KA1 (RSK1)	7	8	8
RPS6KA2 (RSK3)	4	6	5
RPS6KA3 (RSK2)	-7	0	-3
RPS6KA4 (MSK2)	0	3	1
RPS6KA5 (MSK1)	13	6	9
RPS6KA6 (RSK4)	0	1	0
RPS6KB1 (p70S6K)	15	2	9
RPS6KB2 (p70S6Kb)	2	7	4
SBK1	2	3	2
SGK (SGK1)	1	9	5
SGK2	11	-3	4
SGKL (SGK3)	5	15	10
SNF1LK2	-1	5	2
SRC N1	5	3	4
SRC	14	-3	6
SRMS (Srm)	7	13	10
SRPK1	-1	6	2
SRPK2	9	5	7
STK22B (TSSK2)	1	-6	-2
STK22D (TSSK1)	-5	-5	-5
STK23 (MSSK1)	4	-4	0
STK24 (MST3)	0	10	5
STK25 (YSK1)	7	3	5
STK3 (MST2)	-9	-9	-9
STK4 (MST1)	-2	4	1
SYK	5	6	6
TAOK2 (TAO1)	-8	-7	-8
TBK1	-1	1	0
TEK (TIE2) Y897S	2	2	2
TEK (Tie2)	1	-3	-1
TNK1	2	-7	-3
TXK	0	8	4
TYK2	4	10	7

TYRO3 (RSE)	3	7	5
YES1	-2	7	3
ZAP70	24	25	24

**SSBK Adapta SP Technology:**			
CAMK1 (CaMK1)	7	15	11
CDK4/cyclin D1	5	8	6
CDK4/cyclin D3	10	-1	4
CDK6/cyclin D1	-14	8	-3
CDK7/cyclin H/MNAT1	-6	1	-3
CDK9/cyclin T1	-3	-3	-3
CHUK (IKK alpha)	10	-1	4
DAPK1	-10	-6	-8
GSG2 (Haspin)	6	13	10
IRAK1	-13	-6	-10
LRRK2	-12	-12	-12
LRRK2 FL	7	7	7
LRRK2 G2019S	-10	13	1
LRRK2 G2019S FL	19	11	15
LRRK2 I2020T	3	-1	1
LRRK2 R1441C	-5	7	1
NUAK1 (ARK5)	-7	0	-3
PI4K2A (PI4K2 alpha)	20	5	13
PI4K2B (PI4K2 beta)	18	13	16
PI4KA (PI4K alpha)	-3	-5	-4
PI4KB (PI4K beta)	-1	2	1
PIK3C2A (PI3K-C2 alpha)	-10	-10	-10
PIK3C2B (PI3K-C2 beta)	11	5	8
PIK3C2G (PI3K-C2 gamma)	7	15	11
PIK3C3 (hVPS34)	3	-13	-5
PIK3CA E542K/PIK3R1 (p110 alpha E542K/p85 alpha)	6	6	6
PIK3CA E545K/PIK3R1 (p110 alpha E545K/p85 alpha)	5	11	8
PIK3CA/PIK3R1 (p110 alpha/p85 alpha)	2	-15	-6
PIK3CA/PIK3R3 (p110 alpha/p55 gamma)	-4	4	0
PIK3CB/PIK3R1 (p110 beta/p85 alpha)	-5	7	1
PIK3CB/PIK3R2 (p110 beta/p85 beta)	3	26	15
PIK3CD/PIK3R1 (p110 delta/p85 alpha)	-31	-19	-25
PIK3CG (p110 gamma)	-18	-12	-15
PIP4K2A	8	-11	-1
PIP5K1A	0	0	0
PIP5K1B	-2	4	1
PIP5K1C	-12	8	-2
SPHK1	-7	-9	-8
SPHK2	10	-10	0

SSBK LanthaScreen Binding SP Technology:			
AAK1	0	1	1
ABL1 H396P	2	12	7
ABL1 M351T	16	31	24
ABL1 Q252H	6	13	10
ACVR1 (ALK2)	15	10	12
ACVR1 (ALK2) R206H	7	-7	0
ACVR2A	13	-5	4
ACVR2B	3	-1	1
ACVRL1 (ALK1)	12	11	12
ADCK3	7	17	12
ALK C1156Y	1	7	4
ALK F1174L	-3	5	1
ALK L1196M	-3	-2	-3
ALK R1275Q	-2	-2	-2
ALK T1151_L1152insT	7	0	3
AMPK (A1/B1/G2)	-3	-23	-13
AMPK (A1/B1/G3)	-1	-6	-4
AMPK (A1/B2/G1)	12	1	6
AMPK (A2/B2/G1)	20	5	12
AMPK (A2/B2/G2)	20	28	24
ANKK1	0	-7	-4
AXL R499C	0	10	5
BMPR1A (ALK3)	5	8	6
BMPR1B (ALK6)	7	-7	0
BMPR2	3	23	13
BRAF	12	2	7
BRAF V599E	4	-4	0
BRSK2	0	9	4
CAMK2G (CaMKII gamma)	2	1	1
CAMKK1 (CAMKKA)	-7	1	-3
CAMKK2 (CaMKK beta)	-4	3	-1
CASK	20	15	18
CDC7/DBF4	-14	-16	-15
CDK11 (Inactive)	11	12	12
CDK11/cyclin C	24	22	23
CDK13/cyclin K	37	40	38
CDK14 (PFTK1)/cyclin Y	50	36	43
CDK16 (PCTK1)/cyclin Y	4	11	8
CDK2/cyclin A1	10	-3	3
CDK2/cyclin E1	-1	-7	-4
CDK2/cyclin O	8	5	7
CDK3/cyclin E1	11	-2	4
CDK5 (Inactive)	1	0	1
CDK8/cyclin C	22	14	18
CDK9 (Inactive)	2	-11	-5
CDK9/cyclin K	-13	-7	-10
CLK4	5	1	3
DAPK2	27	20	23
DDR1	-1	1	0
DDR2	-2	-4	-3
DDR2 N456S	31	22	26
DDR2 T654M	-6	-12	-9
DMPK	-6	9	2
DYRK2	-5	4	-1
EGFR (ErbB1) d746-750	11	-6	2
EGFR (ErbB1) d747-749 A750P	-10	-3	-6
EIF2AK2 (PKR)	0	13	6
EPHA3	0	13	7
EPHA6	2	0	1
EPHA7	0	0	0
ERN1	-10	1	-4
ERN2	-1	19	9
FGFR1 V561M	15	-7	4
FGFR3 G697C	-6	3	-1
FGFR3 K650M	-1	5	2
FLT3 ITD	-11	-19	-15

FYN A	-18	2	-8
GAK	17	17	17
GRK1	3	3	3
HUNK	5	-4	1
ICK	-2	-9	-6
IRAK3	23	7	15
KIT A829P	0	-6	-3
KIT D816H	1	-4	-2
KIT D816V	20	31	26
KIT D820E	-7	-2	-4
KIT N822K	-4	-2	-3
KIT T670E	-4	6	1
KIT V559D T670I	-1	-4	-3
KIT V654A	8	20	14
KIT Y823D	8	4	6
LATS2	12	2	7
LIMK1	11	1	6
LIMK2	-2	-5	-3
MAP2K1 (MEK1)	30	21	26
MAP2K1 (MEK1) S218D S222D	-5	8	2
MAP2K2 (MEK2)	5	4	5
MAP2K4 (MEK4)	0	-4	-2
MAP2K5 (MEK5)	-10	-3	-6
MAP2K6 (MKK6)	7	-3	2
MAP2K6 (MKK6) S207E T211E	10	12	11
MAP3K10 (MLK2)	-1	3	1
MAP3K11 (MLK3)	5	2	3
MAP3K14 (NIK)	27	6	16
MAP3K2 (MEKK2)	17	5	11
MAP3K3 (MEKK3)	-8	-20	-14
MAP3K5 (ASK1)	24	11	17
MAP3K7/MAP3K7IP1 (TAK1-TAB1)	3	0	2
MAP4K1 (HPK1)	15	16	15
MAP4K3 (GLK)	7	5	6
MAPK10 (JNK3)	1	-2	-1
MAPK15 (ERK7)	5	-2	1
MAPK8 (JNK1)	-16	-1	-9
MAPK9 (JNK2)	-11	0	-5
MASTL	-4	-12	-8
MERTK (cMER) A708S	-2	-4	-3
MET D1228H	-25	-2	-13
MKNK2 (MNK2)	-1	7	3
MLCK (MLCK2)	-5	-1	-3
MLK4	3	19	11
MYLK (MLCK)	6	0	3
MYLK4	-3	-6	-5
MYO3A (MYO3 alpha)	-10	-1	-6
MYO3B (MYO3 beta)	4	4	4
NEK8	7	-2	2
NLK	8	1	5
NUAK2	17	-3	7
PKMYT1	-5	5	0
PKN2 (PRK2)	2	-10	-4
PLK4	-4	2	-1
PRKACB (PRKAC beta)	-3	4	1
PRKACG (PRKAC gamma)	8	3	6
RAF1 (cRAF) Y340D Y341D	-2	-3	-3
RET G691S	-12	-4	-8
RET M918T	20	-1	9
RET V804M	5	12	8
RIPK2	12	-1	5
RIPK3	4	17	10
SIK1	-4	-3	-3
SIK3	10	-3	4
SLK	7	1	4
STK16 (PKL12)	19	14	17
STK17A (DRAK1)	17	9	13

STK17B (DRAK2)	24	4	14
STK32B (YANK2)	0	23	12
STK32C (YANK3)	11	4	8
STK33	15	-2	6
STK38 (NDR)	-8	-8	-8
STK38L (NDR2)	25	5	15
STK39 (STLK3)	15	11	13
TAOK1	0	0	0
TAOK3 (JIK)	6	-16	-5
TEC	-3	2	0
TEK (TIE2) R849W	16	21	19
TEK (TIE2) Y1108F	-14	-5	-9
TESK1	13	1	7
TESK2	12	-1	6
TGFBR1 (ALK5)	0	-2	-1
TGFBR2	19	-5	7
TLK1	-2	16	7
TLK2	12	10	11
TNIK	-1	1	0
TNK2 (ACK)	3	3	3
TTK	9	1	5
ULK1	7	-11	-2
ULK2	7	9	8
ULK3	-7	0	-4
VRK2	-14	-9	-11
WEE1	0	18	9
WNK1	-2	7	2
WNK2	0	4	2
WNK3	-15	-4	-10
ZAK	2	4	3

Safety pharmacology studies

Screening for off-target pharmacology across multiple drug target classes was performed in the DiscoverX Safety47™ panel as the SAFETYscan E/IC50 ELECT service by eurofins (San Diego CA, U.S.A.). The assays were performed utilizing the PathHunter enzyme fragment complementation (EFC) technology, FLIPR®-based cellular screening assays, KINOME*scan* kinase binding assays, and a variety of enzymatic assays (78 assays).

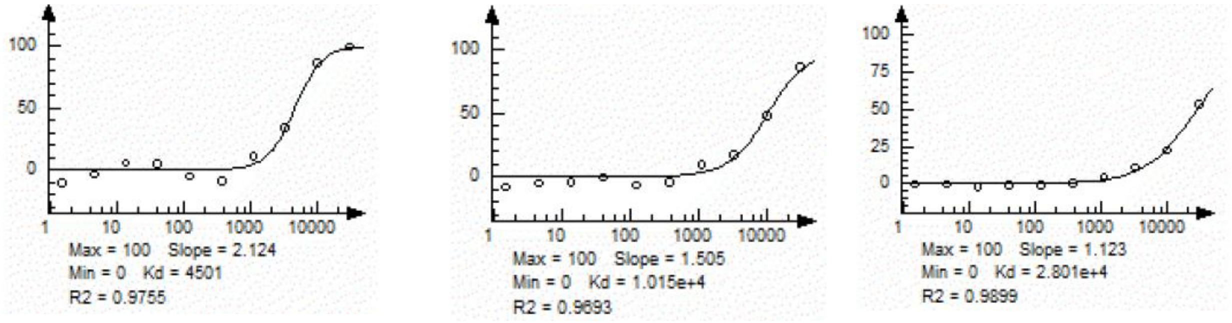

HTR3A antagonist
RC_{50} 4.50 µM

nAChR(a4/b2) antagonist
RC_{50} 10.15 µM

MAOA
RC_{50} 28.0 µM

Figure S14. Concentration-response curves for **8l** in HTR3A, nAChR(a4/b2) and MAOA

Table S21. Summary of Results.

Compound Name	Order ID	Target Class	Assay Name	Mode	Assay Target	Result Type	Value Prefix	RC50 (µM)	Hill	Curve Bottom	Curve Top	Max Response
ARUK3001185	US073-0006420-O	GPCR	Calcium Flux	Agonist	ADORA2A	EC50	>	30				0
ARUK3001185	US073-0006420-O	GPCR	Calcium Flux	Agonist	ADRA1A	EC50	>	30				0
ARUK3001185	US073-0006420-O	GPCR	Calcium Flux	Agonist	AVPR1A	EC50	>	30				0
ARUK3001185	US073-0006420-O	GPCR	Calcium Flux	Agonist	CCKAR	EC50	>	30				0
ARUK3001185	US073-0006420-O	GPCR	Calcium Flux	Agonist	CHRM1	EC50	>	30				0
ARUK3001185	US073-0006420-O	GPCR	Calcium Flux	Agonist	CHRM3	EC50	>	30				0
ARUK3001185	US073-0006420-O	GPCR	Calcium Flux	Agonist	EDNRA	EC50	>	30				0
ARUK3001185	US073-0006420-O	GPCR	Calcium Flux	Agonist	HRH1	EC50	>	30				0
ARUK3001185	US073-0006420-O	GPCR	Calcium Flux	Agonist	HTR2A	EC50	>	30				0
ARUK3001185	US073-0006420-O	GPCR	Calcium Flux	Agonist	HTR2B	EC50	>	30				0
ARUK3001185	US073-0006420-O	GPCR	Calcium Flux	Antagonist	ADORA2A	IC50	>	30				19.75
ARUK3001185	US073-0006420-O	GPCR	Calcium Flux	Antagonist	ADRA1A	IC50	>	30				11.38
ARUK3001185	US073-0006420-O	GPCR	Calcium Flux	Antagonist	AVPR1A	IC50	>	30				0.67
ARUK3001185	US073-0006420-O	GPCR	Calcium Flux	Antagonist	CCKAR	IC50	>	30				0
ARUK3001185	US073-0006420-O	GPCR	Calcium Flux	Antagonist	CHRM1	IC50	>	30				0
ARUK3001185	US073-0006420-O	GPCR	Calcium Flux	Antagonist	CHRM3	IC50	>	30				0
ARUK3001185	US073-0006420-O	GPCR	Calcium Flux	Antagonist	EDNRA	IC50	>	30				0
ARUK3001185	US073-0006420-O	GPCR	Calcium Flux	Antagonist	HRH1	IC50	>	30				5.7
ARUK3001185	US073-0006420-O	GPCR	Calcium Flux	Antagonist	HTR2A	IC50	>	30				0
ARUK3001185	US073-0006420-O	GPCR	Calcium Flux	Antagonist	HTR2B	IC50	>	30				38.36
ARUK3001185	US073-0006420-O	GPCR	cAMP	Agonist	ADRA2A	EC50	>	30				0
ARUK3001185	US073-0006420-O	GPCR	cAMP	Agonist	ADRB1	EC50	>	30				1.72
ARUK3001185	US073-0006420-O	GPCR	cAMP	Agonist	ADRB2	EC50	>	30				0
ARUK3001185	US073-0006420-O	GPCR	cAMP	Agonist	CHRM2	EC50	>	30				0
ARUK3001185	US073-0006420-O	GPCR	cAMP	Agonist	CNR1	EC50	>	30				8.82
ARUK3001185	US073-0006420-O	GPCR	cAMP	Agonist	CNR2	EC50	>	30				12.39

Compound Name	Order ID	Target Class	Assay Name	Mode	Assay Target	Result Type	Value Prefix	RC50 (µM)	Hill	Curve Bottom	Curve Top	Max Response
ARUK3001185	US073-0006420-O	GPCR	cAMP	Agonist	DRD1	EC50	>	30				1.98
ARUK3001185	US073-0006420-O	GPCR	cAMP	Agonist	DRD2S	EC50	>	30				0
ARUK3001185	US073-0006420-O	GPCR	cAMP	Agonist	HRH2	EC50	>	30				0.81
ARUK3001185	US073-0006420-O	GPCR	cAMP	Agonist	HTR1A	EC50	>	30				8.16
ARUK3001185	US073-0006420-O	GPCR	cAMP	Agonist	HTR1B	EC50	>	30				4.3
ARUK3001185	US073-0006420-O	GPCR	cAMP	Agonist	OPRD1	EC50	>	30				4.96
ARUK3001185	US073-0006420-O	GPCR	cAMP	Agonist	OPRK1	EC50	>	30				0
ARUK3001185	US073-0006420-O	GPCR	cAMP	Agonist	OPRM1	EC50	>	30				0
ARUK3001185	US073-0006420-O	GPCR	cAMP	Antagonist	ADRA2A	IC50	>	30				5.86
ARUK3001185	US073-0006420-O	GPCR	cAMP	Antagonist	ADRB1	IC50	>	30				14.11
ARUK3001185	US073-0006420-O	GPCR	cAMP	Antagonist	ADRB2	IC50	>	30				20.27
ARUK3001185	US073-0006420-O	GPCR	cAMP	Antagonist	CHRM2	IC50	>	30				3.15
ARUK3001185	US073-0006420-O	GPCR	cAMP	Antagonist	CNR1	IC50	>	30				3.15
ARUK3001185	US073-0006420-O	GPCR	cAMP	Antagonist	CNR2	IC50	>	30				0
ARUK3001185	US073-0006420-O	GPCR	cAMP	Antagonist	DRD1	IC50	>	30				11.7
ARUK3001185	US073-0006420-O	GPCR	cAMP	Antagonist	DRD2S	IC50	>	30				0.22
ARUK3001185	US073-0006420-O	GPCR	cAMP	Antagonist	HRH2	IC50	>	30				10.61
ARUK3001185	US073-0006420-O	GPCR	cAMP	Antagonist	HTR1A	IC50	>	30				1.55
ARUK3001185	US073-0006420-O	GPCR	cAMP	Antagonist	HTR1B	IC50	>	30				0
ARUK3001185	US073-0006420-O	GPCR	cAMP	Antagonist	OPRD1	IC50	>	30				0.94
ARUK3001185	US073-0006420-O	GPCR	cAMP	Antagonist	OPRK1	IC50	>	30				1.33
ARUK3001185	US073-0006420-O	GPCR	cAMP	Antagonist	OPRM1	IC50	>	30				2.25
ARUK3001185	US073-0006420-O	Ion Channel	Ion Channel	Blocker	CAV1.2	IC50	>	30				7.98
ARUK3001185	US073-0006420-O	Ion Channel	Ion Channel	Blocker	GABAA	IC50	>	30				22.4
ARUK3001185	US073-0006420-O	Ion Channel	Ion Channel	Blocker	hERG	IC50	>	30				25.84
ARUK3001185	US073-0006420-O	Ion Channel	Ion Channel	Blocker	HTR3A	IC50	=	4.50122	2.12	0	100	98.97

Compound Name	Order ID	Target Class	Assay Name	Mode	Assay Target	Result Type	Value Prefix	RC50 (µM)	Hill	Curve Bottom	Curve Top	Max Response
ARUK3001185	US073-0006420-O	Ion Channel	Ion Channel	Blocker	KvLQT1/minK	IC50	>	30				17.29
ARUK3001185	US073-0006420-O	Ion Channel	Ion Channel	Blocker	nAChR(a4/b2)	IC50	=	10.14752	1.5	0	100	85.64
ARUK3001185	US073-0006420-O	Ion Channel	Ion Channel	Blocker	NAV1.5	IC50	>	30				12.34
ARUK3001185	US073-0006420-O	Ion Channel	Ion Channel	Blocker	NMDAR (1A/2B)	IC50	>	30				0
ARUK3001185	US073-0006420-O	Ion Channel	Ion Channel	Opener	GABAA	EC50	>	30				9.12
ARUK3001185	US073-0006420-O	Ion Channel	Ion Channel	Opener	HTR3A	EC50	>	30				0.15
ARUK3001185	US073-0006420-O	Ion Channel	Ion Channel	Opener	KvLQT1/minK	EC50	>	30				0.52
ARUK3001185	US073-0006420-O	Ion Channel	Ion Channel	Opener	nAChR(a4/b2)	EC50	>	30				4.91
ARUK3001185	US073-0006420-O	Ion Channel	Ion Channel	Opener	NMDAR (1A/2B)	EC50	>	30				0
ARUK3001185	US073-0006420-O	Kinases	Binding	Inhibitor	INSR	IC50	>	30				4.34
ARUK3001185	US073-0006420-O	Kinases	Binding	Inhibitor	LCK	IC50	>	30				5.85
ARUK3001185	US073-0006420-O	Kinases	Binding	Inhibitor	ROCK1	IC50	>	30				1.22
ARUK3001185	US073-0006420-O	Kinases	Binding	Inhibitor	VEGFR2	IC50	>	30				16.39
ARUK3001185	US073-0006420-O	NHR	NHR Nuclear Translocation	Agonist	AR	EC50	>	30				0
ARUK3001185	US073-0006420-O	NHR	NHR Nuclear Translocation	Antagonist	AR	IC50	>	30				3.44
ARUK3001185	US073-0006420-O	NHR	NHR Protein Interaction	Agonist	GR	EC50	>	30				0.21
ARUK3001185	US073-0006420-O	NHR	NHR Protein Interaction	Antagonist	GR	IC50	>	30				27.12
ARUK3001185	US073-0006420-O	Non-Kinase Enzymes	Enzymatic	Inhibitor	AChE	IC50	>	30				12.57
ARUK3001185	US073-0006420-O	Non-Kinase Enzymes	Enzymatic	Inhibitor	COX1	IC50	>	30				9.75
ARUK3001185	US073-0006420-O	Non-Kinase Enzymes	Enzymatic	Inhibitor	COX2	IC50	>	30				6.86
ARUK3001185	US073-0006420-O	Non-Kinase Enzymes	Enzymatic	Inhibitor	MAOA	IC50	=	28.01496	1.12	0	100	52.63
ARUK3001185	US073-0006420-O	Non-Kinase Enzymes	Enzymatic	Inhibitor	PDE3A	IC50	>	30				43.04
ARUK3001185	US073-0006420-O	Non-Kinase Enzymes	Enzymatic	Inhibitor	PDE4D2	IC50	>	30				9.13
ARUK3001185	US073-0006420-O	Transporter	Transporter	Blocker	DAT	IC50	>	30				0
ARUK3001185	US073-0006420-O	Transporter	Transporter	Blocker	NET	IC50	>	30				11.05
ARUK3001185	US073-0006420-O	Transporter	Transporter	Blocker	SERT	IC50	>	30				7.3

Note, ARUK3001185 is the compound identifier for **8l.**

Differential Scanning Calorimetry for 1-azido-2,4-dichloro-3-(trifluoromethyl)benzene (12a)

Differential Scanning Calorimetry (DSC) was performed by DEKRA (Southampton, U.K.).

Apparatus and Method:

Apparatus:	TA Instruments Discovery DSC25
Data acquisition:	TA Instruments TRIOS software
Crucible type:	Gold-lined steel (high pressure, sealed)
Start temperature:	25 °C
Trip temperature:	400 °C
Ramp rate:	4 $K.min^{-1}$
Test atmosphere:	air
Sample mass:	4.42 mg

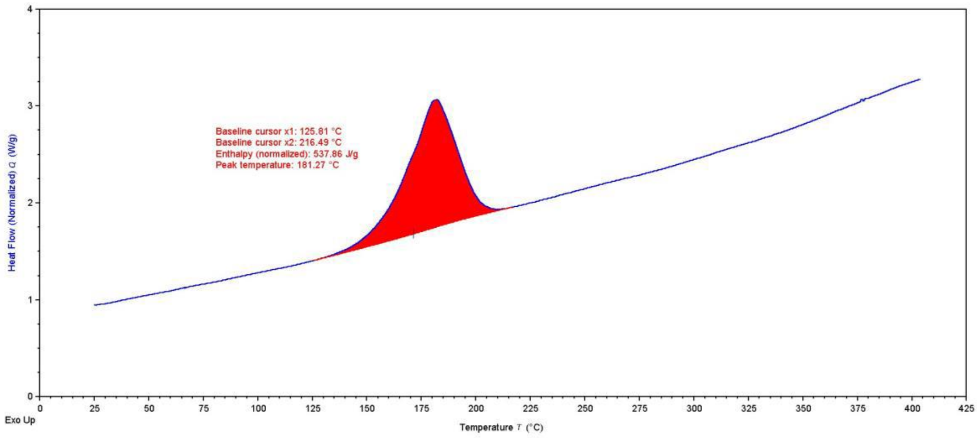

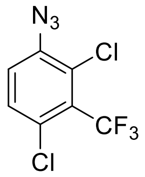

12a

TA Instruments Trios V5.1.0.46403

Figure S15. DSC showing heat flow v temperature for **12a**

Table S22. Summary of DSC test results.

Exotherm onset temperature (°C)	125.8
Heat of decomposition ($J.g^{-1}$)	-537.9
Adiabatic temperature rise (K)	269
Estimated T_{D24} (°C)	42
**Maximum safe handling temperature (°C)**	**42**

X-ray structure determination for 6b and 8l: data collection and refinement statistics, and ligand electron-density maps

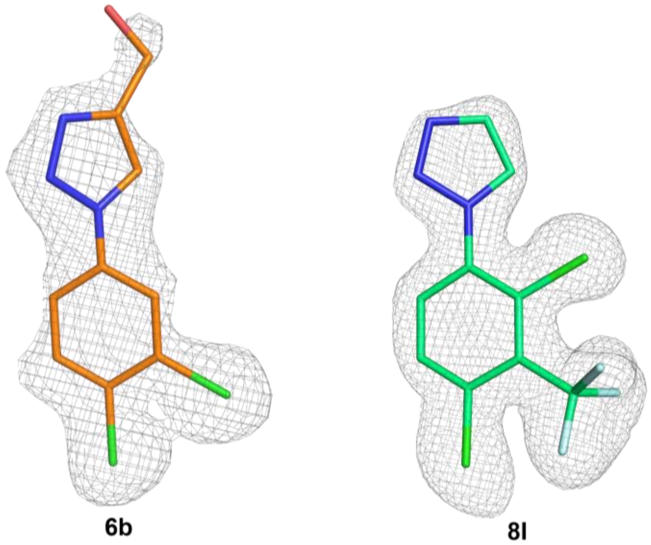

Figure S16. Electron density maps for **6b** and **8l**. The |Fo-Fc| omit maps were shown as grey mesh for **6b** (orange sticks) and **8l** (light green sticks) with countour level of 3σ.

Table S23. Data collection and refinement statistics for 6b and 8l

	Notum_**6b**	Notum_**8l**
PDB ID code	7B89	7PK3
**Data collection**		
X-ray source (Diamond)	I04-1	I04
Wavelength (Å)	0.9282	0.9750
Space group	$P2_12_12_1$	$P2_12_12_1$
Cell dimensions		
a, *b*, *c* (Å)	60.5, 72.4, 78.8	60.1, 71.5, 78.4
α, β, γ (°)	90, 90, 90	90, 90, 90
Resolution (Å)	1.84 (1.84-1.87) *	1.41 (1.41 -1.43)
R_{merge}	0.20 (---)	0.09 (---)
I / σ*I*	10.6 (1.3)	12.5 (1.0)
CC 1/2	0.97 (0.61)	0.99 (0.45)
Completeness (%)	100 (97.0)	100 (98.8)

Redundancy	6.4 (5.8)	12.7 (9.7)
**Refinement**		
Resolution (Å)	53-1.84	52.8 - 1.41
No. reflections	30571 (2947)	65706 (6488)
R_{work} / R_{free}	0.210/0.259	0.177/0.204
No. atoms		
Protein	2851	2986
Ligand/other	15/73	17/43
Water	105	127
B-factors (Å^2)		
All atoms	23	25
Ligand	35	37
R.m.s. deviations		
Bond lengths (Å)	0.008	0.005
Bond angles (°)	0.94	0.79

*Values in parentheses are for highest-resolution shell.

Spectroscopic and analytical data for 1-(2,4-dichloro-3-(trifluoromethyl)phenyl)-1*H*-1,2,3-triazole (8l)

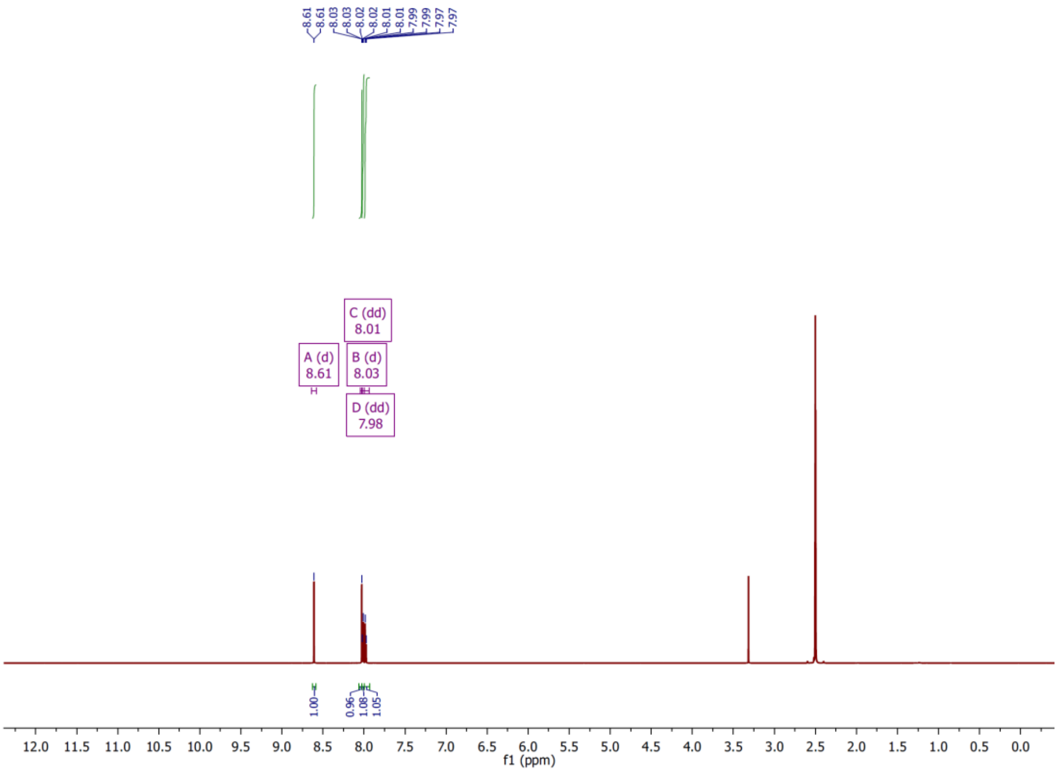

Figure S17. ^{1}H NMR (700 MHz, d_6-DMSO) for **8l**.

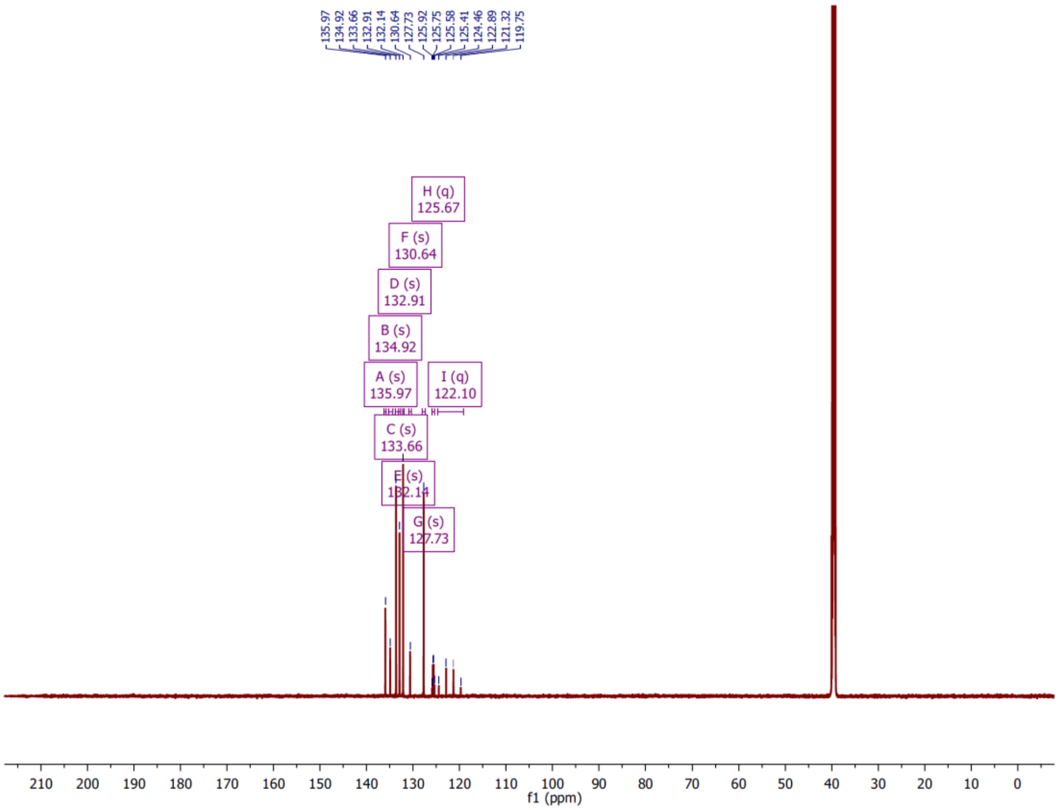

Figure S18. ^{13}C NMR (176 MHz, d_6-DMSO) for **8l**.

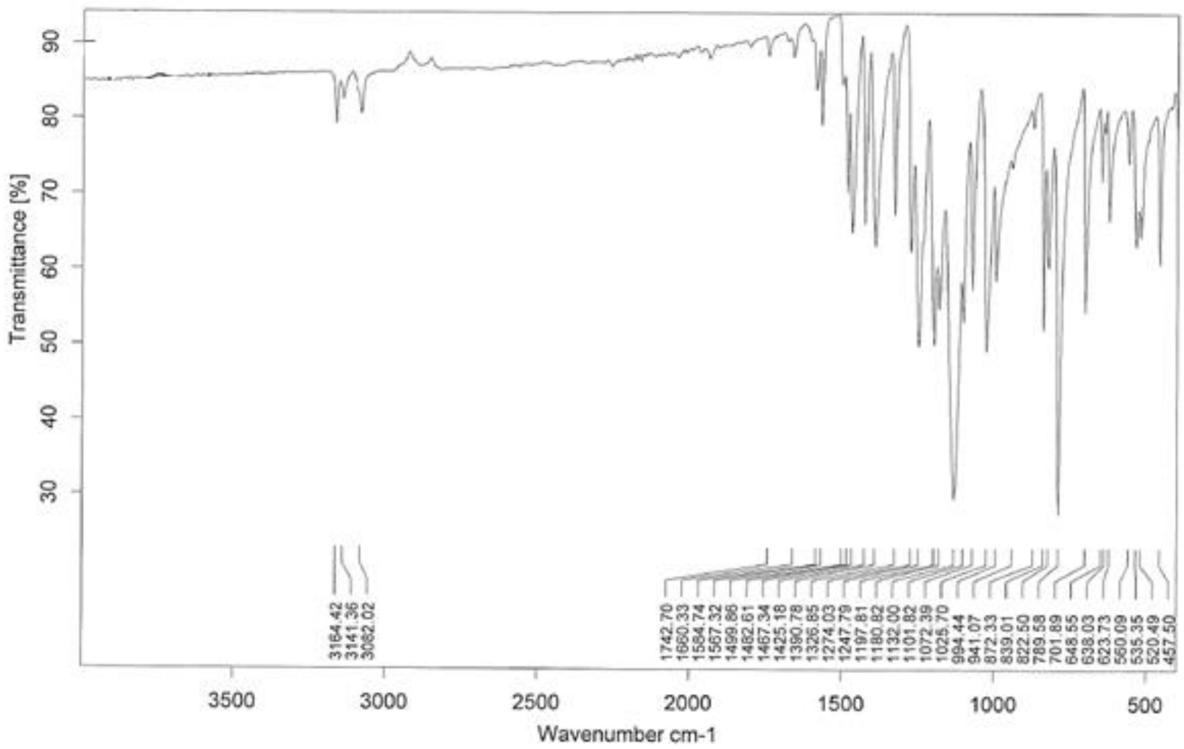

Figure S19. FTIR for **8l**.

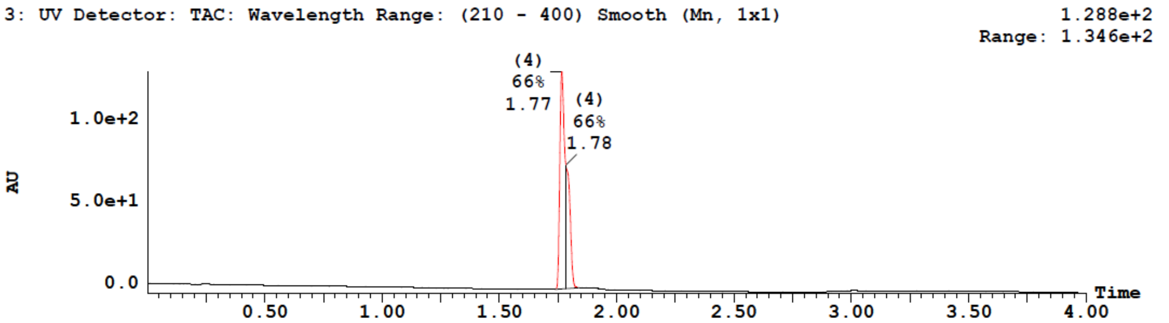

Peak Number	Compound	Time	AreaAbs	Area %Total	Width	Height	Mass Found
4		1.77	3e+006	66.02	0	1e+008	Not Found
4		1.78	2e+006	33.98	0	8e+007	Not Found

Peak ID	Compound	Time	Mass Found
4		1.78	Not Found

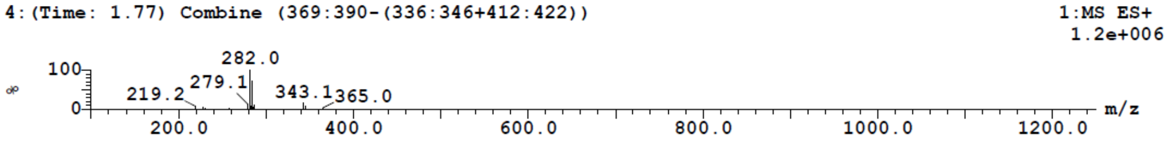

Figure S20. LCMS (basic) for **8l**.

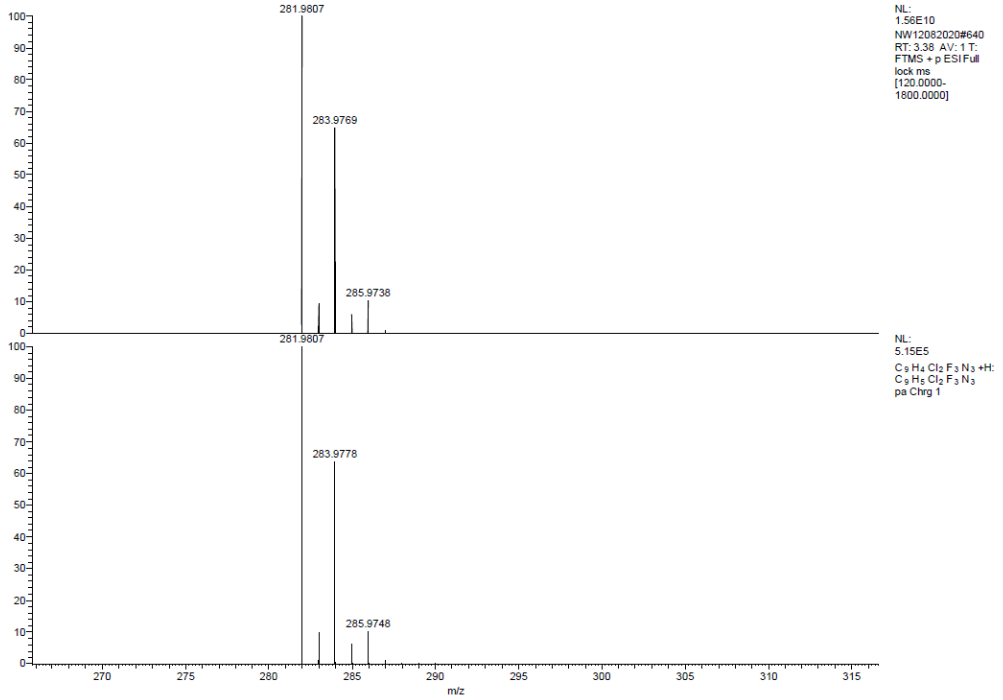

Figure S21. HRMS for **8l**.

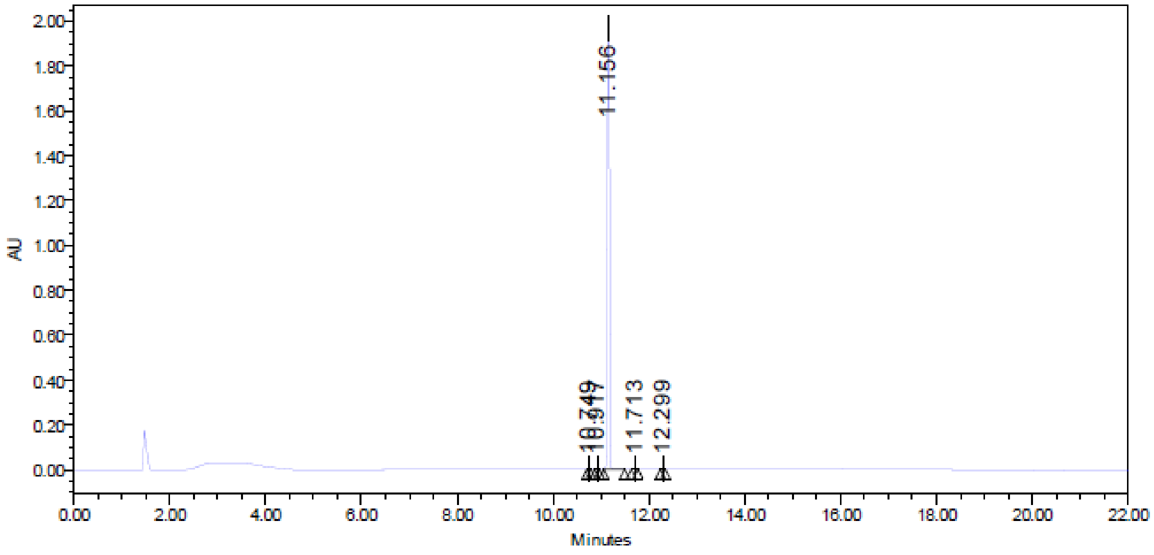

	Retention Time (min)	Area (µV*sec)	% Area	Width (sec)
1	10.75	247	0.00	5.001
2	10.92	895	0.01	6.101
3	11.16	6082021	99.97	26.106
4	11.71	330	0.01	8.352
5	12.30	166	0.00	6.151

Figure S22. UHPLC (C18 CSH; Mobile Phase A: Water (0.1% TFA v/v)); Mobile Phase B: MeCN) for **8l**.

SI References

1. Mahy, W.; Willis, N. J.; Zhao, Y.; Woodward, H. L.; Svensson, F.; Sipthorp, J.; Vecchia, L.; Ruza, R. R.; Hillier, J.; Kjær, S.; Frew, S.; Monaghan, A.; Bictash, M.; Salinas, P. C.; Whiting, P.; Vincent, J. P.; Jones, E. Y.; Fish, P. V. 5-Phenyl-1,3,4-Oxadiazol-2(3*H*)-Ones are potent inhibitors of Notum carboxylesterase activity identified by the optimization of a crystallographic fragment screening hit. *J. Med. Chem.* **2020**, *63*, 12942–12956.
2. Steadman, D.; Atkinson, B. A.; Zhao, Y.; Willis, N. J.; Frew, S.; Monaghan, A.; Patel, C.; Armstrong, E.; Costelloe, K.; Magno, L.; Bictash, M.; Jones, E. Y.; Fish, P. V.; Svensson, F. Virtual screening directly identifies new fragment-sized inhibitors of carboxylesterase Notum with nanomolar activity. *J. Med. Chem.*, **2022**, 65, 562-578.
